# Photoswitchable Molecular Motor Phospholipid: Synthesis, Characterization, and Integration into Lipid Vesicles

**DOI:** 10.1021/acs.langmuir.4c04173

**Published:** 2025-02-03

**Authors:** Ainoa Guinart, Daniel Doellerer, Yusuf Qutbuddin, Henry Zivkovic, Cristina Branca, Dominik Hrebik, Petra Schwille, Ben L. Feringa

**Affiliations:** † Stratingh Institute for Chemistry, University of Groningen, 9747AG Groningen, The Netherlands; ‡ Cellular and Molecular Biophysics, Max Planck Institute of Biochemistry, 82152 Martinsried, Germany; § Cell and Virus Structure, Max Planck Institute of Biochemistry, 82152 Martinsried, Germany

## Abstract

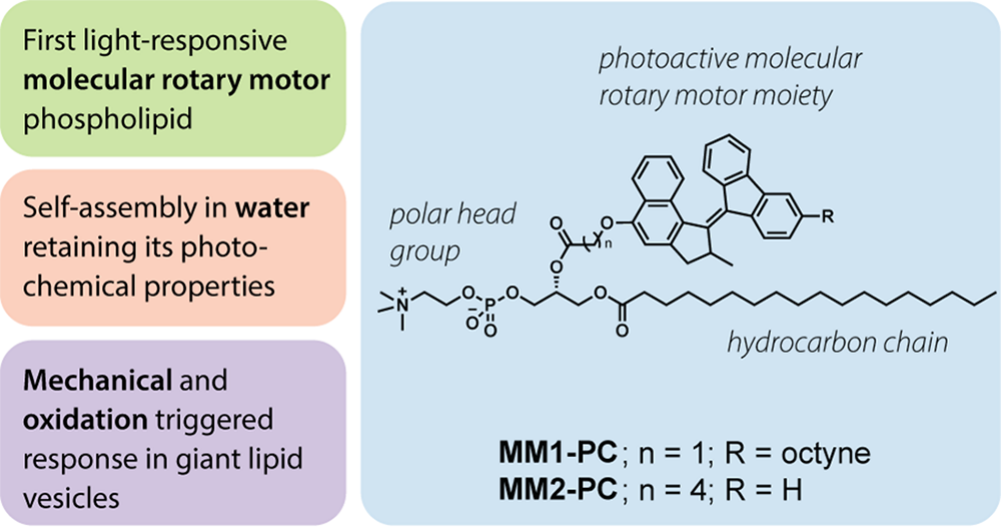

Lipid membranes are essential for cellular function, acting as barriers and platforms for major cellular and biochemical activities. The integration of photoisomerizable units into lipid structures allows for tunable membrane properties, offering insights into major membrane-related processes. In this study, we present the first molecular-motor-conjugated phospholipid system. The synthesis of two phosphatidylcholine derivatives is reported, where one acyl chain is replaced with a light-responsive molecular rotary motor moiety. We explore the photochemical and thermodynamic behaviors of these compounds in solution and as self-assembled systems, demonstrating their rotation cycles under illumination and their dynamic properties in combination with lipid molecules. Additionally, giant unilamellar vesicles with these compounds are formed to investigate the mechanisms of the photoinduced responses in synthetic lipid membranes. Our findings show that molecular motor-based lipids can operate in aqueous solution and with natural phospholipids, maintaining photoisomerization properties and enabling oxidation-driven release within giant lipid vesicles.

## Introduction

The study of lipid membranes is crucial to understanding many biological processes and their underlying mechanisms. Lipid membranes play an essential role in cellular function, serving as barriers that compartmentalize and protect cellular contents, as well as platforms to carry out most biochemical activities in our body.^1^ One of the key features of lipid membranes is the ability of their components to self-assemble into organized structures, driven by the amphipathic nature of their constituent molecules. Amphipathic molecules, such as phospholipids, readily form organized arrays by self-assembly in aqueous environments. Their cooperative behavior is strongly influenced by the length and degree of unsaturation of their acyl chains, as well as their headgroup composition.^2^ For a given headgroup and fixed chain length, *cis*-unsaturation leads to bending in the acyl chain, due to packing effects derived by bond fixation. In contrast, *trans*-compounds exhibit more cooperative phase transitions.^3^ The introduction of isomerizable units into the lipid structure offers the possibility to control membrane physical behavior, opening the door to tunable membrane characteristics such as fluidity,^4^ packing area,^5^ order,^6^ and lipid rafts^7^ among others. This regulation is highly relevant for studying the effects of bilayer physics and biochemistry in areas such as ion transport, membrane-bound enzyme activity, and membrane mechano-sensitivity. By modulating the structural properties of the membrane, we can gain insights into how different lipid compositions influence biological functions. Naturally occurring photoisomerization of phospholipids only happens at rather short wavelengths, which often involves photochemical decomposition.^8^ In recent years, several photoisomerizable phospholipid analogs have been developed, highlighting the potential for controlled manipulation of membrane properties through photoisomerizing mechanisms.^9−11^ Most of these examples are based on azobenzene photoswitches conjugated to a phospholipid backbone in one or both of its acyl chains. Studies report light-triggered changes in membrane properties^12−15^ as well as fluidity^16,17^ and lipid domain reorganization.^18,19^ One of the recent studies demonstrated the dose-dependent function of an azobenzene-derived analog of palmitoyl-oleoylphosphatidylcholine (POPC) in constructing artificial photoswitchable cell mimetics. The authors provide a quantitative link between material properties and membrane parameters such as changes in area and thickness, morphology, elastic and electrical properties, and their relation to organization and restructuring at the molecular level.^5^ Molecular photoswitches, such as azobenzenes, are compounds that interconvert between two different isomer states when irradiated at the appropriate wavelength. These systems hold promise for numerous applications, especially when a transition between two differentiated states is required. Similar to molecular photoswitches, a higher control of molecular motion can be achieved by the so-called overcrowded-alkene molecular rotary motors.^20^ These molecules based on chiroptical molecular switches were first reported in 1999 and are able to perform repetitive, photochemically driven unidirectional motion involving four different molecular states.^21^ Briefly, the rotation cycle is described by the sequential isomerization of the molecule that enforced a unidirectional motion as opposed to a randomized Brownian motion.^22^ In the case of the molecular motors used in this study, this process is characterized by two photochemical *E-Z* isomerizations, each followed by a thermal helix inversion (THI) step that brings the system back to the initial state, resulting in continuous motion as long as there is a photon supply (for more details on molecular motor rotation dynamics see ref (20)). We envision that combining such systems with a phospholipid backbone will achieve a higher degree of nanoscale control in self-assembled systems such as membranes, increasing the number of possible molecular states, together with a repetitive, out-of-equilibrium mechanical motion. Here we report the first molecular motor-phospholipid systems (**MM-PC**) to our knowledge. We discuss the synthesis of two distinct phosphatidylcholine derivatives, where one hydrophobic acyl chain has been replaced by a molecular rotary motor moiety. Investigations of the photochemical and thermodynamic behavior of the novel compounds both in solution and in self-assembled systems are presented. We also provide evidence of their rotation cycle under appropriate illumination as well as studies of the self-assembled behavior in combination with lipid molecules and their light-induced dynamic properties. Finally, we formed giant unilamellar vesicles (GUVs) with our **MM-PC** compounds and their phospholipid analog and explored the possible mechanisms of photoinduced responses in synthetic lipid membranes. In this study, we proved that molecular motor-based lipids can be operated in pure water and in vesicles when combined with natural phospholipids, with increased stability, retaining their photoisomerization properties. Such systems were integrated into giant lipid vesicles to promote cargo release via an unprecedented opening procedure.

## Experimental Section

### Synthesis and Characterization of **MM1-PC** and **MM2-PC**

#### Chemicals

All chemicals were purchased from commercial sources, by name Sigma-Aldrich, Fluorochem, TCI, and BLDpharm, and used without further purification. Dry solvents were obtained from Acros Organics and Alfa Aesar or from a solvent purification system (MBraun SPS-800).

#### Synthesis and Procedures

If not stated otherwise, all reactions were carried out in oven-dried glassware under a nitrogen atmosphere by using standard Schlenk techniques. Solids were added in a counter flow of nitrogen or before crimping the vials and cycled three times between vacuum and nitrogen before the addition of liquids. Solutions and reagents were added to nitrogen-flushed disposable syringes/needles. Analytical thin layer chromatography (TLC) was performed on silica gel 60 G/UV265 aluminum sheets from Merck (0.25 mm). Flash column chromatography was performed on a silica gel Davisil LC60A (Merck type 9385, 230–400 mesh) or a Biotage Selekt system (MPLC) using the indicated solvents. NMR spectra were recorded on a Varian Mercury-Plus 400, a Varian Unity Plus 500, or a Bruker 600 MHz NMR spectrometer at 298 K unless stated otherwise. High-resolution mass spectra (HRMS) were recorded on an LTQ Orbitrap XL spectrometer.

### In Situ Irradiation Sudies

A solution (2.5 mM) of either **MM1-PC** or **MM2-PC** was prepared in methanol-d_4_ and transferred into an NMR tube, which was subsequently fitted with a glass optic fiber for in situ irradiation studies. The sample was placed in a Varian Unity Plus 500 MHz NMR and cooled to −15 °C. ^1^H NMR spectra were recorded before irradiation while irradiating with 405 nm until reaching PSS and during the THI step until completed.

### UV–Vis Spectroscopy

UV–Vis spectroscopy was used for the determination of molecular motor photoisomerization quantum yield determination and thermodynamic studies (see Supporting Information for details). Briefly, samples containing free-standing lipid systems or molecular motor solutions were measured using an Agilent 8453 UV–vis Diode Array System, equipped with a Quantum Northwest Peltier controller. If specified, irradiations were done using a built-in setup coupled to an LED. Solutions were prepared and measured using a quartz cuvette with a 1 cm optical path.

### Fluorescence Lifetime Spectroscopy

MicroTime200 (PicoQuant GmBH, Germany), equipped with a dual SPAD detection unit and a MultiHarp 150 TCSPC unit, was used to measure the fluorescence lifetime of A655-DOPE present in the membrane. SUVs were prepared with the desired concentration of the **MM1-P**C and 0.002 mol % A655-DOPE with a final lipid concentration of 2 mM. The SUVs were deposited on a #1.5 coverslip and excited with a 641 nm laser line, and the emission was collected through a 50 μm pinhole. Single photon counting histograms were collected for 180 s for each measurement. SymphoTime64 was used to analyze and fit the decay curves and obtain the fluorescence lifetime for each case.

### Cryo-Electron Microscopy

Three μL of SUVs prepared at 2.5 mg/mL were applied on a glow-discharged Quantifoil 1.2/1.3 holey carbon grid, Cu 300 mesh (Quantifoil Micro Tools GmbH, Germany), and blotted for 3.5 s in 100% humidity at 20 °C and immediately plunge-frozen into an ethane-propane 1:1 mixture using the Leica EM GP2 (Leica, Germany). Grids were then loaded into a Titan Krios G4 transmission electron microscope operated at 300 kV, equipped with a Falcon4i direct detector camera, Selectris X energy filter, and CFEG electron source (ThermoFisher Scientific, USA).

### Small Unilamellar Vesicles (SUVs) Preparation

1-palmitoyl-2-oleoyl-*sn*-glycero-3-phosphocholine (POPC) purchased from Avanti Polar Lipids (Alabaster, USA) was used as the single phospholipid composition. For SUV preparation, the desired lipids and photoactive molecules were combined in chloroform at the specified molar ratio. The resulting mixture was transferred to a glass vial, and the solvent was removed using a stream of N_2_ gas, followed by vacuum drying in a desiccator to ensure complete removal of residual solvents. To initiate hydration, Milli-Q water was added to the lipid film, achieving a final lipid concentration of 10 mM. This was followed by vigorous vortexing to create a suspension of multilamellar vesicles. Subsequently, these suspensions were further diluted if necessary (CryoEM samples were diluted to 2.5 mM). To promote vesicle homogenization and size reduction, the samples underwent three freeze–thaw cycles following sonication for 15 min.

### Confocal Imaging of GUVs

GUVs were prepared through electroformation with Pt electrodes (see Supporting Information). Spinning disk confocal imaging was performed on a Nikon/Yokogawa CSU-W1 spinning disk confocal microscope using 405, 488, and 641 nm laser lines. The 50 μm pinhole spinning disk was used at 4000 rpm. The sample was illuminated through a Nikon Apo TIRF 60x Oil DIC N2 immersion oil objective, and the images were acquired in pco.edge sCMOS cameras (pco.edge 4.2 LT USB) at 100 ms exposure time. For z-stack imaging, the desired optical sectioning was set to 0.2 μm.

## Results and Discussion

### Compound Synthesis

**Scheme 1:**
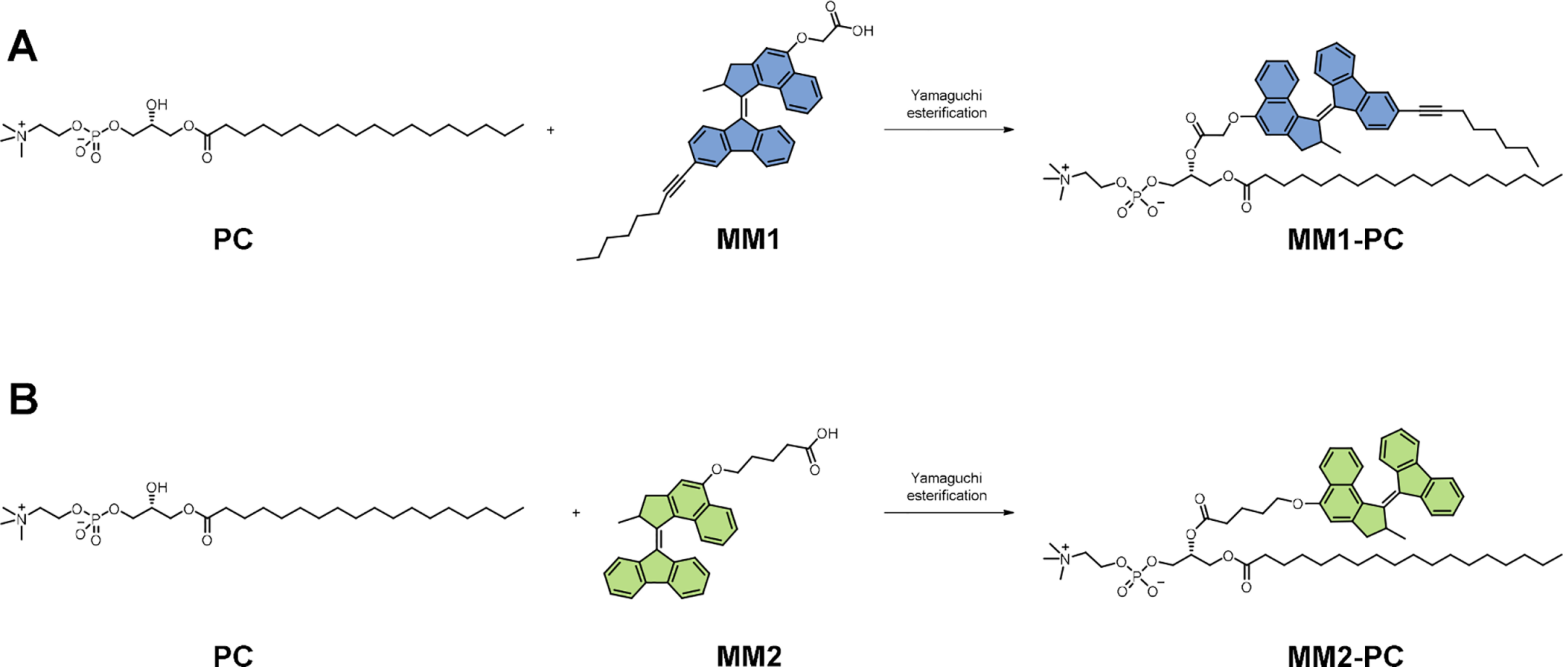
General Scheme for the Final Step Synthesis Step, a Yamaguchi Esterification between the Phospholipid Chain (PC) and the Photoactive Molecular Motor Moiety (**MM1** and **MM2**) (A) For **MM1-PC**. (B) For **MM2-PC**.

Molecular motors **MM1** and **MM2** were used as building blocks to synthesize the desired molecular motor-modified phospholipids **MM1-PC** and **MM2-PC** using modifications of established procedures (Supporting Information Scheme S1 and ESI for detailed conditions). In short, **1** was synthesized by reacting 1-methoxynaphthalene with methacrylic acid in polyphosphoric acid (PPA) following a Friedel–Crafts acylation mechanism and a Nazarov cyclization.^23^ A Williamson ether synthesis was performed on phenolic version **2**, which was obtained by deprotection of **1** with pyridine hydrochloride,^24^ followed by ether bond formation using the respective bromoalkylate and potassium carbonate as the base, yielding substituted ketones **3a** and **3b**. Next, **3a**/**3b** were transformed into the respective thioketones **4a**/**4b** using Lawesson’s reagent in toluene. To obtain the diazo compounds **6a** and **6b** for the coupling, the corresponding fluorenone derivative was transformed using hydrazine monohydrate to generate hydrazones **5a** and **5b**, and consecutively oxidized with manganese dioxide yielding **6a** and **6b**.^25^ With both, the thioketone (**4a**/**4b**) and the diazo (**6a**/**6b**) compound in hand, a Barton-Kellogg coupling reaction was performed, using hexamethylphosphorous triamide as a desulfuration agent, resulting in molecular motors **7a** and **7b**. A Sonogashira cross-coupling between motor **7a**, containing a shorter spacer at the top part than **7b**, and 1-octyne, using *N*,*N*-diisopropylethylamine (DIPEA) as a base, dimethylformamide as a solvent, and CuI and Pd(PPh_3_)Cl_2_ as catalysts, yielded the octyne-substituted motor **8** in an *E*/*Z* mixture. It should be emphasized that triethylamine as a base and tetrahydrofuran (THF) as a solvent at 60 °C did not show any sign of product formation. The last step before the formation of the photoactive phospholipids was a saponification of compounds **7b** and **8** with sodium hydroxide, generating the free acid-containing molecules **MM1** and **MM2** (Scheme 1). The phospholipid precursor, containing a hydrocarbon chain and polar headgroup, was synthesized by reacting (*R*)-2,3-dihydroxypropyl (2-(trimethylammonio)ethyl) with stearoyl chloride in the presence of dibutyltin oxide and triethylamine, catalyzing a selective reaction at the primary alcohol.^16^ The photoactive phospholipids **MM1-PC** and **MM2-PC** were formed via Yamaguchi esterification under the exclusion of light. 2,4,6-Trichlorobenzoyl chloride was employed to form a mixed anhydride with **MM1** or **MM2**, which further reacted with the secondary alcohol moiety of **PC** and 1-methylimidazole as a base to form esters **MM1-PC** and **MM2-PC** (Scheme 1A and 1B, respectively).^16^

### Observation of the Rotation Cycle

**Figure 1:**
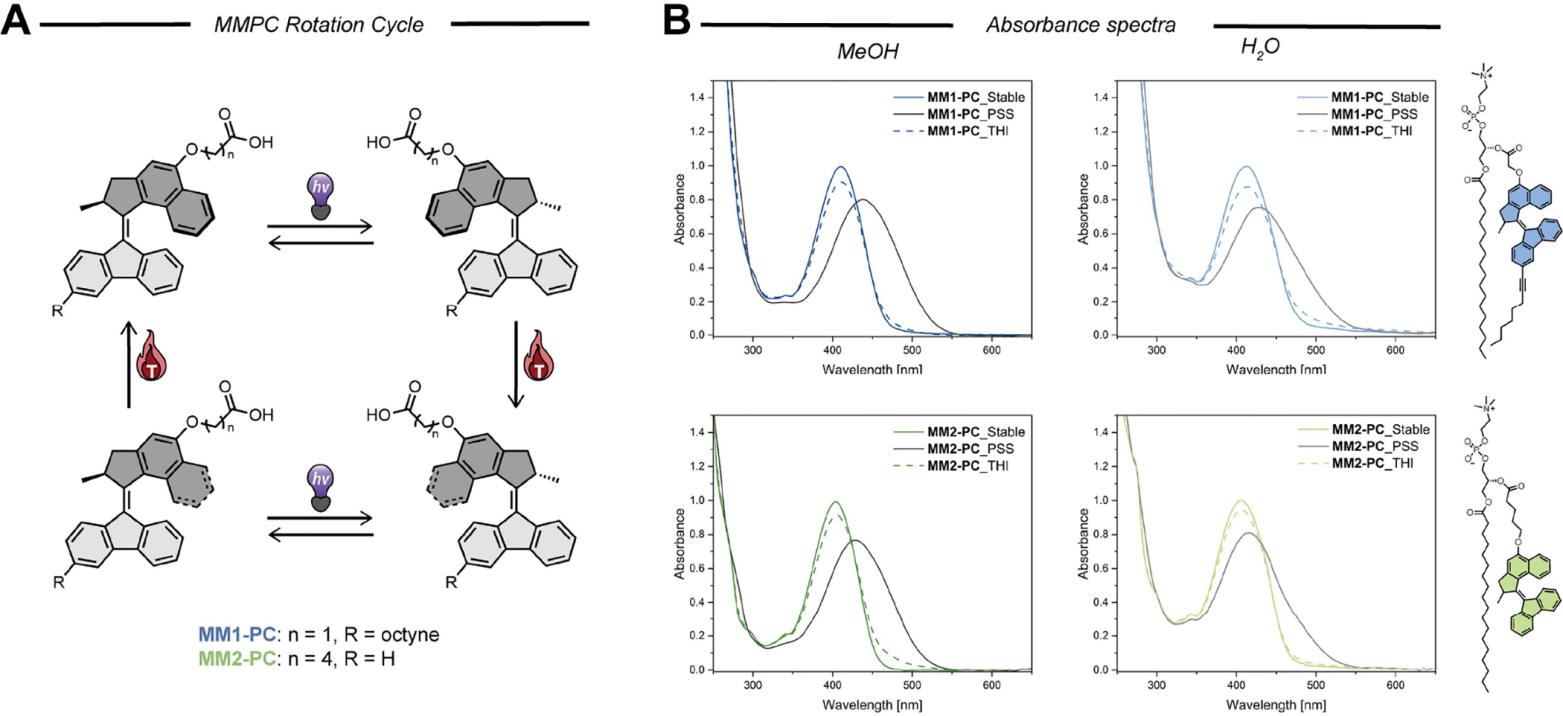
(A) **MMPC** rotation cycle. (B) Absorption spectra of pure **MM1-PC** (top, blue) and **MM2-PC** (bottom, green) in MeOH (left column) or self-assembled in aq solution (right column). Colored lines show the spectra before irradiation with 405 nm light, black lines represent the spectra for each compound after reaching PSS, and dotted colored lines indicate the recovered absorption spectra after THI under dark conditions.

A schematic representation of the rotation cycle is depicted in Figure 1A. The photochemical properties of **MM1-PC** and **MM2-PC** were investigated by using UV–vis absorption spectroscopy under different conditions. Methanol was used as a reference organic solvent for lipid solutions.^26^ Self-assembled samples in aq solution composed of pure **MM-PCs** or in combination with different fractions of POPC were also studied. Measurements were performed on the stable isomers of each compound. All spectra showed broad absorption bands with the maxima corresponding to the double bound isomerization transition (S_0_ → S_1_) centered between 404 and 418 nm, tailoring into the visible region (up to 550 nm, colored line Figure 1B). We found that **MM1-PC** is slightly red-shifted compared to **MM2-PC** which can be attributed to the 3-position of the bottom half conjugated octyne moiety to the double bound. This effect is translated into a red-shift of the maximum absorption wavelength of around 5 nm (Table 1). Interestingly, all compounds showed a slight bathochromic shift for the self-assembled systems. Changes in the absorption spectra of a molecule when going from solution to a self-assembled state typically indicate variations in its structural organization and intramolecular interactions.^27^ This effect is more pronounced for the self-assembled systems in conjugation with POPC molecules and seems to increase with the amount of POPC present in the assembly (Table 1, Figure S4). A similar effect has been previously described for azobenzene-modified phospholipids and is attributed to the formation of H/J-aggregates.^16,28^ These molecules tend to organize by forming small aggregates in the lipid bilayer, favoring face-to-face noncovalent π–π interactions.

**Table 1:** Position of Absorbance Maxima [nm] of the Double Bound Transition for the Stable Isomer of **MM1-PC** and **MM2-PC** in Different Environments

compound	MeOH	H_2_O	50 mol % inPOPC	25 mol %in POPC	10 mol % in POPC
**MM1-PC**	410	415	416	417	418
**MM2-PC**	404	409	411	412	412

Irradiation of the stable isomer of **MM1-PC** and **MM2-PC** with a 405 nm LED at 20 °C produced a change in the absorption spectra with a clear isosbestic point (dark line in Figures 1, S5, and S6). The resulting steady-state spectrum corresponds to the partial formation of the metastable isomer at the photostationary state (PSS). In accordance with previously reported second-generation molecular motors with five-membered upper and lower halves, the metastable form of **MM1-PC** and **MM2-PC** is significantly red-shifted compared to the stable isomer.^20^ This is attributed to a decrease in the HOMO–LUMO gap of the metastable form based on earlier computational calculations in similar molecular motor structures.^29,30^

**Figure 2:**
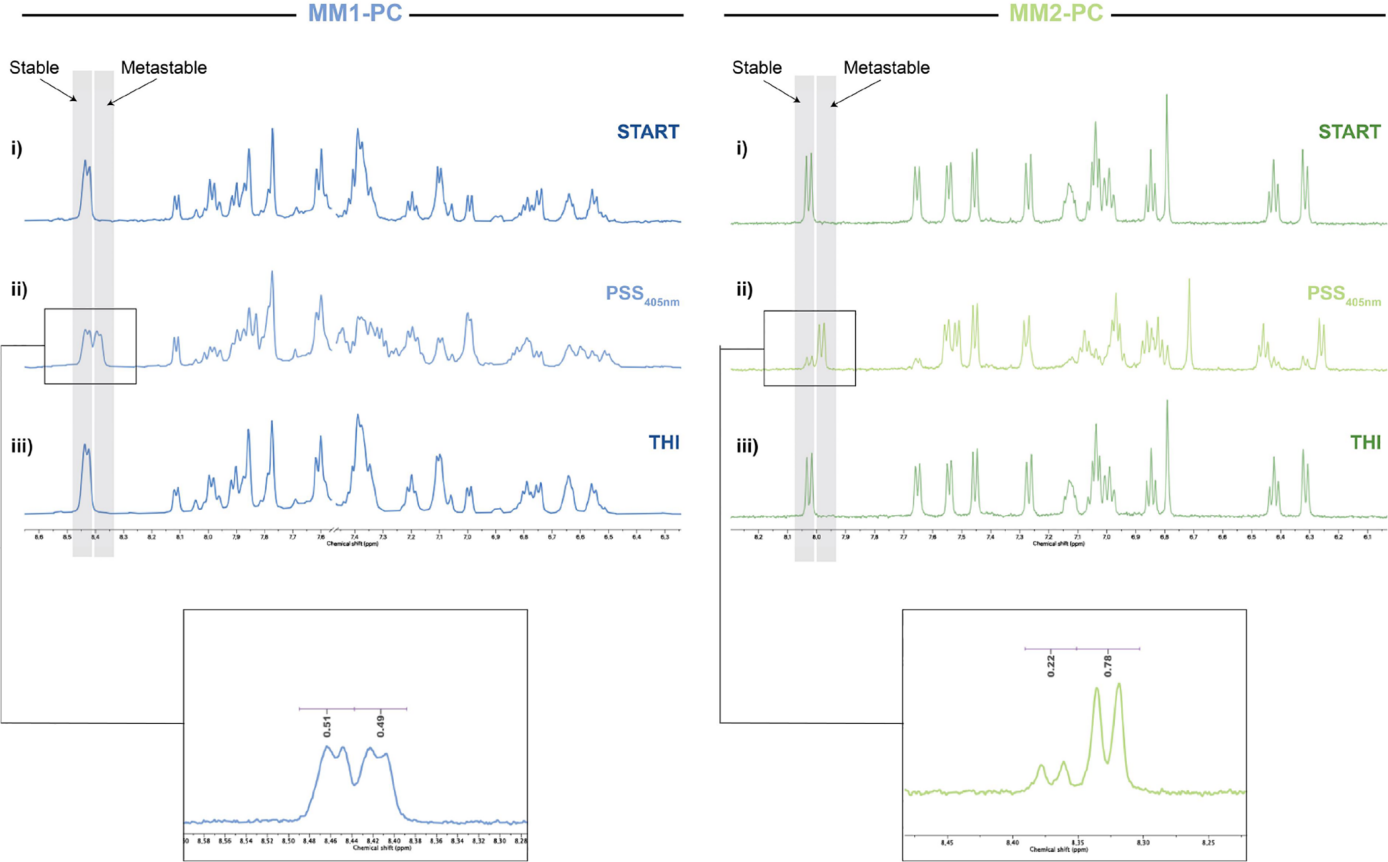
^1^H NMR irradiation studies (methanol-d_4_, *c* = 2.5 mM, 15 °C) of **MM1-PC** (left) and **MM2-PC** (right). Starting from its stable form (i), spectra change under 405 nm irradiation in situ irradiation of the stable isomer until reaching PSS (ii) and after undergoing THI under dark conditions (iii). The inset corresponds to the stable/metastable distribution at PSS.

A detailed analysis of the rotation cycle of **MM1-PC** and **MM2-PC** was performed using low-temperature ^1^H NMR spectroscopy (Figures 2 and S1–S3). Irradiations were carried out in situ directly in the NMR probe, using a glass fiber optic cable connected to a 405 nm LED at −15 °C in MeOD, for solubility reasons. For both compounds, constant irradiation of the stable isomer led to a decrease in the peak intensity of the protons corresponding to the stable state isomer, and a new set of signals appeared at the same rate, which were assigned to the metastable isomer, showing a selective isomerization profile (Figure 2). After 30 min of constant irradiation at 405 nm, a photostationary state was reached with a stable-to-metastable isomer ratio of 51:49 and 22:78 for **MM1-PC** and **MM2-PC**, respectively. Removing the light source led to a complete recovery of the stable state signals after overnight in the dark.

### Analysis of the Photoisomerization Behavior and Thermodynamic Parameters

The quantum yield of the photoisomerization was also determined in all the previously studied systems (MeOH, H_2_O, and in conjugation with POPC molecules) using the method outlined by Stranius and Börjesson^31^ which follows the evolution of both components of the photostationary distribution (PSD) when irradiated at a known concentration. This method has been extensively used for molecular motors to determine their quantum yields of photoisomerization.^32,33^ To assess the photochemically driven isomerization of **MM1-PC** and **MM2-PC**, we subjected each sample to irradiation with a 405 nm LED and monitored the changes in the absorption spectra at the irradiation wavelength until the PSS was reached. For **MM1-PC** and **MM2-PC,** this process corresponded to quantum yields of 3.2 ± 0.19 and 5.81 ± 0.12%, respectively.

When the same measurements were carried out in self-assembled systems, a drop in the quantum yield value was expected, as seen in previous studies of molecular motors embedded into crowded lipid environments, hindering the photoisomerization process.^34^ Indeed, both **MM1-PC** and **MM2-PC** showed a sequential decrease in their quantum yield values with increasing concentrations of the molecules in POPC self-assembled systems (Table S1). Interestingly, the pure system in water showed the lowest quantum yield value for **MM1-PC** but just a small decrease for **MM2-PC** (Table S1). We envision that these results might be related to different self-assembled structures for the two compounds in water.

Eyring analysis of the studied compounds was performed to determine the activation parameters of the thermal isomerization step corresponding to the metastable to the stable transition of **MM1-PC** and **MM2-PC**. The rate of the THI step was determined by following the decay in the absorbance at 450 nm. The exponential decay was followed at three different temperatures (15, 20, and 25 °C) in all MeOH, H_2_O, and lipid solutions. To calculate the activation parameters of the thermal barrier, the rate constants of the exponential decay function are obtained for each temperature and fitted using the linearized Eyring equation (Table S3, Figures S8 and S9). The calculated experimental barriers of both **MM1-** and **MM2-PC** were found to increase when going from a solution in methanol to the self-assembled structures in water (ESI Table S2). This effect was more pronounced with increasing concentrations of the motor compounds in POPC self-assemblies in water, reaching its maxima with the pure compound in water for **MM1-PC** (*t*_1/2_ of 192 and 528 s in MeOH and H_2_O, respectively). Interestingly, a similar behavior was found for increasing concentrations of **MM2-PC** in conjugation with POPC; however, the compound exhibited a sharp decrease of the activation barrier for the pure water condition (*t*_1/2_ of 162 and 58 s in MeOH and H_2_O, respectively). The differences in the half-lives of **MM1-** and **MM2-PC** between the two solvents can be attributed to the formation of more (in the case of **MM2-PC**) or less (in the case of **MM1-PC**) favorable self-assembled structures in water for the THI step.

### Fatigue Resistance in Aqueous Environment

**Figure 3:**
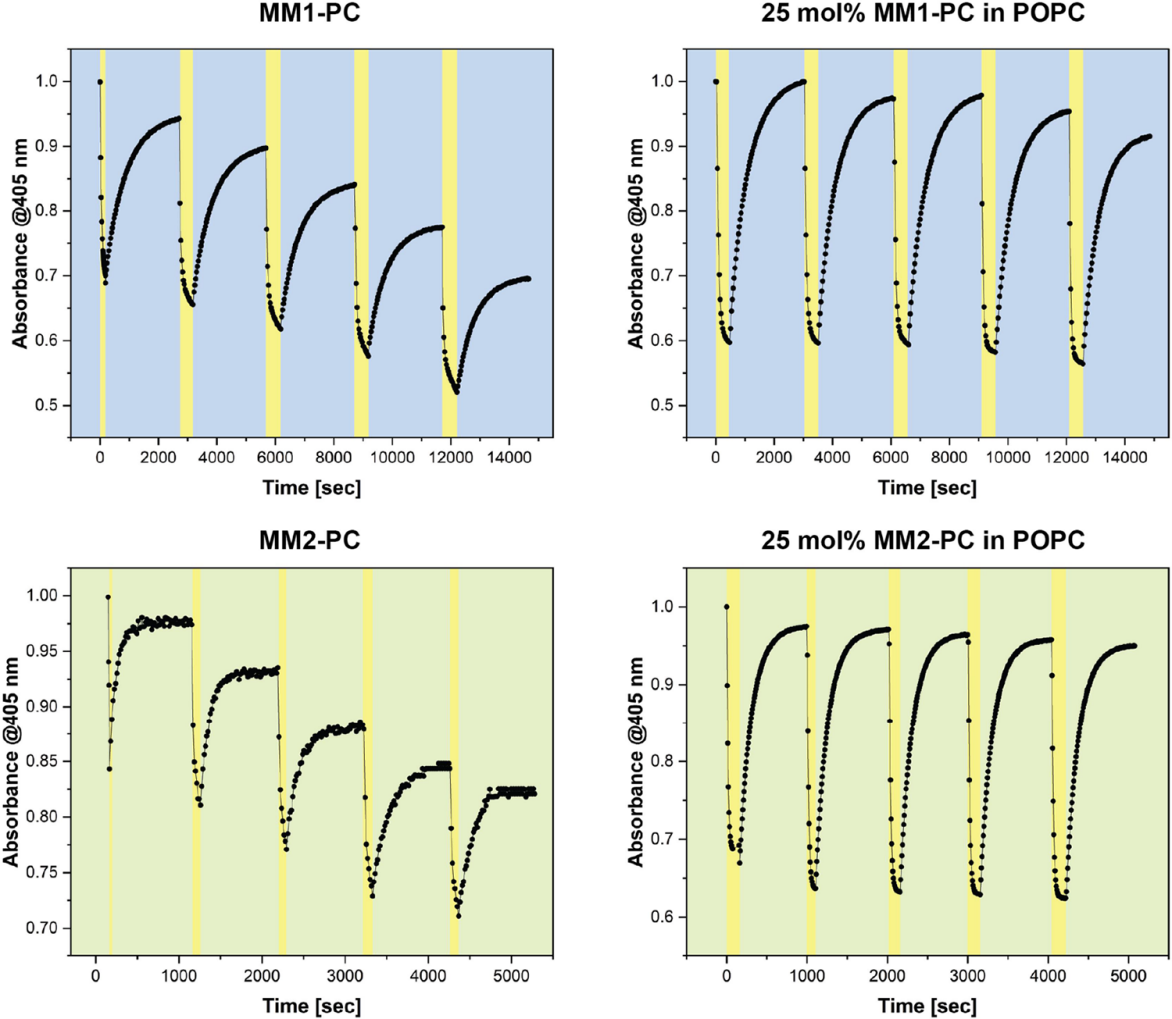
Changes in the absorbance for fatigue studies of **MM1-PC** and **MM2-PC** over the course of five successive PSS/THI processes under 405 nm irradiation.

As the ultimate application of a lipid molecular motor requires their functioning in an aqueous environment, further studies were carried out under these conditions. First, the stability of the nonirradiated compounds in water was assessed during 1 week using UV–vis spectroscopy, and no sign of degradation was observed (Figure S7). However, upon prolonged 405 nm irradiation, apparent degradation by a general decrease in absorption over the whole spectrum was observed for both **MM1-** and **MM2-PC** in pure water. Fatigue studies of sequential PSS/THI processes over 5 cycles show a decrease in the 405 nm absorbance of around 5% for **MM1-PC** and 2.5% for **MM2-PC** after each cycle (Figure 3, left). This degradation has been reported in the past for water-soluble molecular motors and is attributed to a twisted and polarized conformation of the central double bound of the photogenerated intermediate which makes it more vulnerable to water addition.^34^ Interestingly, in all cases, the unstable isomer thermally isomerizes back to the stable form in a clean process if the irradiation is halted. This phenomenon has also been reported for other compounds such as dihydroquinolines.^35^ Remarkably, we could solve the issue of reduced stability (fatigue) in water by combining the vulnerable molecules within lipid environments due to some kind of hydrophobic shielding effect.^36^ We were pleased to observe that the incorporation of 25 mol % of **MM1-PC** and **MM2-PC** in POPC systems retained the stability of the compounds under illumination (Figure 3, right), and fatigue resistance studies over 5 cycles show a major increase in the retention of the absorbance spectra at 405 nm irradiation. These results indicate the improved performance of the molecules in conjugation with phospholipid environments, and these combinations were used for the subsequent studies (vide infra).

### Self-Assembly and Incorporation into GUVs

The ability of **MM1-PC** and **MM2-PC** to self-assemble in water was investigated by determining their critical aggregation concentration (CAC) using a Nile Red fluorescence assay (see ESI for details). The results revealed CAC values of 24.4 and 0.78 μM for **MM1-PC** and **MM2-PC**, respectively, before irradiation of the samples. Interestingly, after 1 min of irradiation with a 405 nm LED, the CAC value decreased more than 3-fold for **MM1-PC** (7.56 μM) and slightly for **MM2-PC** (0.58 μM) (Table S4, Figure S12). SUVs of 50 mol % of **MM1-PC** together with POPC were prepared and imaged using cryo-electron microscopy (see Supporting Information for details). The images were classified and averaged to evaluate their membrane before and after illumination with 405 nm light (see Figure S11). No significant changes were observed in the membrane thickness.

**Figure 4:**
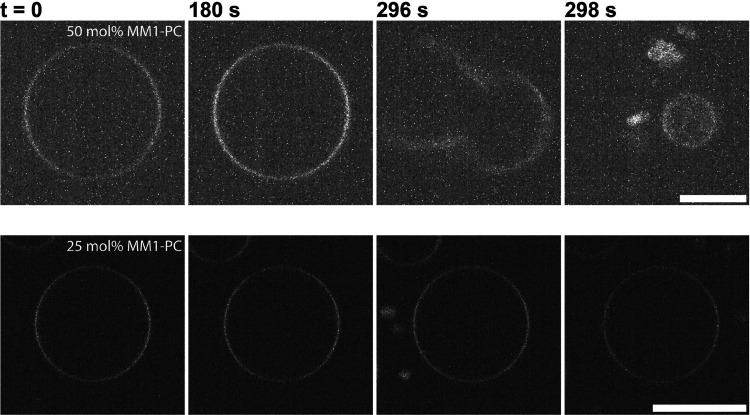
(top) Explosion of GUVs composed of POPC:**MM1-PC** (1:1) upon irradiation with 405 nm laser. (bottom) GUVs composed of POPC:**MM1-PC** (3:1) upon irradiation with 405 nm laser do not show any explosion. Scale bar: 10 μm.

Next, we examined the incorporation of **MM1-PC** and **MM2-PC** to GUVs (∼20 μm in diameter) composed of different ratios of **MM1-PC** and **MM2-PC**, POPC, and 1 mol % of Atto655-DOPE (for fluorescent imaging). To prepare the GUVs for experimentation, we employed the electroformation method (see ESI for details). Image acquisition was performed using spinning disk confocal microscopy, which allows for rapid acquisition via acquiring thin optical sections and illumination of the whole sample volume. As soon as the imaging started, we noticed a decrease in the expected fluorescent intensity (sample of only POPC with 1 mol % Atto655-DOPE). Examining the literature for similar compounds based on azobenzene-modified lipids, we found that these compounds are well-known dark quenchers for certain fluorophores.^37^ In the case of azobenzene photoswitches and in our **MMPCs,** a photochemical isomerization at the central double bound is the preferred pathway to deactivate the electronic excited state back to the ground state in a nonradiative manner. Consequently, these molecules are usually weakly fluorescent or nonfluorescent compounds. Thus, in specific molecular systems where these systems are in close proximity to molecular dyes with certain characteristics, an effective Förster resonance energy transfer (FRET) can occur.^38^ In this scenario, **MM1-PC** and **MM2-PC** can act as acceptors, dissipating energy through nonradiative pathways. As a consequence, the fluorescent donor, Atto655-DOPE in our case, is turned off as long as the FRET process occurs. In a FRET event, energy transfer from the excited donor to the acceptor takes place, leading to the quenching of the donor’s fluorescence. Consequently, the fluorescence lifetime, defined as the average time a molecule remains in its excited state before emitting a photon, would decrease. This is because the added FRET acceptor provides an additional nonradiative pathway for the excited state of the donor to relax, accelerating the overall decay process. We observed a significant reduction in the fluorescence lifetime of A655-DOPE with increasing concentrations of **MM1-PC** in lipid membranes (see Figure S10). Interestingly, when we started irradiating with 405 nm light, a slow but significant increase of the fluorescence could be observed. In the case of photoswitchable lipids based on azobenzene, it is well documented how the quenching of the fluorophores is significantly stronger for the *cis* isomer compared to the *trans* one.^39,40^ In our scenario, the molecular motor keeps a continuous unidirectional rotation between the different states, which most likely uninterruptedly remodels the phospholipid distribution inside the membrane. Based on this observation, we decided to form GUVs with **MM1-PC** and study them under optical microscopy. We observed that the fluorescence intensity of the membrane dye slowly increases upon irradiation under 405 nm. Following an increase in fluorescence intensity, vesicles do explode and fragment into smaller daughter vesicles (see Figure 4). The absence of membrane fluctuations rules out the possibility of over extension of the membrane due to area expansion. Such light-triggered vesicle explosions have been previously reported^41,42^ where the generation of ROS increased the osmotic pressure inside the vesicle resulting in membrane rupture. As reported in previous sections, we propose that the degradation of **MM1-PC** upon irradiation results in the generation of ROS (hydroxyl radicals), which could interact with sucrose present inside the vesicles. It has been shown that sucrose can act as a hydroxyl radical scavenger,^43^ where the radical attacks at the glucose moiety creating a scission at the glycosidic bond resulting in a fructose and a glucose radical. This would effectively increase the osmolarity in the lumen of the membrane. Degradation of the **MM1-PC** can be slowed down with a lower concentration of **MM1-PC** incorporated in the membrane, as has been shown by fatigue resistance studies (see Figure 3). However, the light-triggered vesicle explosions observed in our study present a unique mechanism distinct from those of previously reported processes involving ROS generation. While ROS production, such as the generation of hydroxyl radicals during the partial degradation of **MM1-PC,** plays a role, our findings suggest that the explosion mechanism involves a combination of ROS and mechanical effects. Lipid vesicle explosions driven by commonly reported photosensitizers such as chlorin e6 or methylene blue occur in a notably different sequence of morphological changes.^44,45^ Photosensitizer-induced photodynamic damage typically involves distinct phases, including violent membrane fluctuations caused by lipid oxidation, surface area expansion, and repetitive swell-burst cycles.^46,47^ In contrast, the gradual process in our study lacks such a behavior.

Given the extensive knowledge acquired over the past decades regarding the control of molecular motor rotation dynamics and parameters, we suggest that unprecedented control over the release from lipid systems can be achieved using similar molecules. This would offer a unique ability to design GUVs composed of homogeneous lipid systems, which could be triggered on demand to explode and release their contents without changing the headgroup composition of the membrane and without the need for light-sensitive molecules to be present inside the lumen. At lower ratios of **MM1-PC** in the membrane (see Figure 4 (bottom)), we do not observe vesicle explosion, which indicates a combination effect of ROS production and the mechanical effect of the motors. Studies with Langmuir monolayers would be essential to accurately estimate the mechanical effect of **MM1-PC** in the future.^48^

It is important to clarify that the conformational change observed after switching off the irradiation source is primarily attributed to a THI rather than an *E-Z* thermal back isomerization. While both processes are theoretically possible, the THI is the most likely dominant mechanism in our case. This is due to the significantly lower energy barrier for helix inversion compared to that required for *E-Z* isomerization, which is typically considered a nonreversible process. These energies and preferred isomerization pathways unequivocally demonstrate the rotatory process that has been extensively studied previously in the parent motors **MM1** and **MM2**, and substituted analogs prove the unidirectionality of the rotation cycle. This suggests that their underlying mechanism remains unaffected after the incorporation of an acyl chain. In contrast, if the molecules were to undergo back isomerization instead of the expected four-step rotation cycle, the resulting molecules would reassemble photoswitchable lipids, which are known to induce dramatic changes in the conformation of GUVs. However, our experiments show a distinct behavior for GUVs in the presence of **MM1-PC** and **MM2-PC**, similar to previously reported unidirectional molecular motors in lipid membranes,^34^ which we attribute to the continuous four-step rotation cycle of the motors, producing gradual conformational changes across all states, rather than switching between two well-defined states. Thus, while we cannot provide direct proof of the THI, the available evidence and ample precedence in related substituted second-generation motors strongly support its role as the predominant process in these systems.

## Conclusions

In this study, we report the first two photoswitchable lipids based on molecular rotary motors. The synthesis and characterization of two molecular motor-modified phospholipids (**MM1-PC** and **MM2-PC**) are described, and the analysis of their light-driven rotation cycle in solution and aqueous self-assembled environments is provided by means of UV–vis and low-temperature NMR spectroscopy. We observe a decrease in their photoisomerization quantum yield in self-assembled systems, which we attribute to the constraints of a more restricted environment. The thermodynamic parameters and activation energy profiles of **MM1-PC** and **MM2-PC** suggest distinct structural arrangements in aqueous solutions. We further explored their self-assembly behavior, measuring the CAC, which notably decreased upon irradiation. Fatigue resistance studies under aqueous conditions revealed the susceptibility of both molecules to degradation under prolonged irradiation, which is a common limitation for water-soluble molecular motors. However, embedding these motors within lipid environments significantly enhanced their stability and fatigue resistance, underscoring the protective role of the phospholipid assemblies. These findings point to **MMPCs** combined with phospholipid vesicles as promising candidates for biological applications. A key distinction observed in this study lies in the behavior of GUVs in the presence of these molecules, which contrasts with the behavior of traditional photoswitchable lipids. Specifically, the incorporation of **MM1-PC** triggered a light-induced increase in membrane fluorescence, followed by vesicle fragmentation. This phenomenon is attributed to the unique four-step rotary cycle of **MM1-PC** under illumination, resulting in sustained conformational changes, as opposed to a binary switching mechanism, combined with the generation of reactive oxygen species. This interplay between molecular motor activity and membrane integrity highlights the importance of the active compound’s concentration in dictating system behavior.

In summary, this proof-of-concept study introduces the first photoswitchable lipids integrating molecular rotary motors and demonstrates their functionality within lipid systems. These findings open avenues for diverse applications in biomedical, biochemical, and synthetic cell research, paving the way for future advancements in light-responsive lipid systems.

## Supporting Information Available

Additional information on the synthetic procedures, materials, methods, and supporting data (PDF)
