# Supplementary material for: Photoswitchable Molecular Motor Phospholipid: Synthesis, Characterization, and Integration into Lipid Vesicles
Source: Langmuir. 2025 Feb 3;41(6):3961–70. doi: 10.1021/acs.langmuir.4c04173 (PMC11841041; doi:10.1021/acs.langmuir.4c04173)
Supplement: Supplementary file 1 — la4c04173_si_001.pdf [file la4c04173_si_001.pdf]

Supporting information

Photoswitchable molecular motor phospholipid: synthesis, characterization and integration into lipid vesicles.

*Ainoa Guinart‡, Daniel Doellerer‡, Yusuf Qutbuddin, Henry Zivkovic, Cristina Branca, Dominik Hrebik, John Briggs, Petra Schwille *, and Ben L. Feringa **

‡ These authors contributed equally to this work

* Corresponding author. E-mail: b.l.feringa@rug.nl

Table of contents

1. GENERAL INFORMATION
2. SYNTHESIS
3. NMR IRRADIATION STUDIES
4. UV-VIS SPECTROSCOPY
    a. QUANTUM YIELD DETERMINATION
    b. EYRING ANALYSIS
5. FLUORESENCE SPECTROSCOPY
6. CRYO- ELECTRON MICROSCOPY
7. CRITICAL AGGREGATION CONCENTRATION (CAC)
8. GIANT UNILAMELAR VESICLES (GUVs)
    a. GUVs IMAGING – SPINNING DISK CONFOCAL MICROSCOPY
9. REFERENCES
10. APPENDIX

1. General Information

Chemicals were purchased from commercial sources, by name Sigma-Aldrich, Fluorochem, TCI, BLDpharm and used without further purification. Dry solvents were obtained from Acros Organics, Alfa Aesar or from a solvent purification system (MBraun SPS-800). If not stated otherwise, all reactions were carried out in oven-dried glassware under a nitrogen atmosphere using standard Schlenk techniques. Solids were added in a counter flow of nitrogen or before crimping the vials and cycled three times between vacuum and nitrogen before addition of liquids. Solutions and reagents were added with nitrogen-flushed disposable syringes/needles.

Analytical thin layer chromatography (TLC) was performed on silica gel 60 G/UV265 aluminum sheets from Merck (0.25 mm). Flash column chromatography was performed on silica gel Davisil LC60A (Merck type 9385, 230–400 mesh) or a Biotage Selekt system (MPLC) using the indicated solvents. NMR spectra were recorded on a Varian Mercury-Plus 400, a Varian Unity Plus 500 or a Bruker 600 MHz NMR spectrometer at 298 K unless stated otherwise. Chemical shifts are reported in parts per million (ppm) and referenced to the residual solvent signal ($CDCl_3$: δ = 7.26 for ^{1}H and 77.2 for $^{13}C\{^1H\}$; $(CD_3)_2SO$: δ = 2.50 for ^{1}H and 39.5 for $^{13}C\{^1H\}$; CD_3OD: δ = 3.31 for ^{1}H and 49.0 for $^{13}C\{^1H\}$; CD_2Cl_2: δ = 5.32 for ^{1}H and 53.8 for $^{13}C\{^1H\}$) and thereby relatively stated to TMS. The resonance multiplicity is indicated as s = singlet, d = doublet, t = triplet, q = quartet, p = pentet, m = multiplet, dd (doublet of doublets), dt (doublet of triplets), td(triplet of doublets), ddd (doublet of doublets of doublets) and the coupling constant values (*J*) are given in hertz (Hz). High resolution mass spectra (HRMS) were recorded on a LTQ Orbitrap XL spectrometer.

For ease of display, the structure of **MM1-PC** is shown in its *E* configuration, although the compound was isolated as an *E*/*Z* mixture.

All spectra of the synthesized compounds can be found in the appendix of the Supporting Information.

2. Synthesis

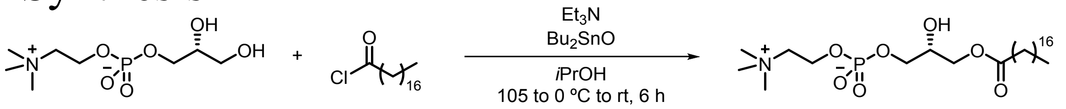

PC

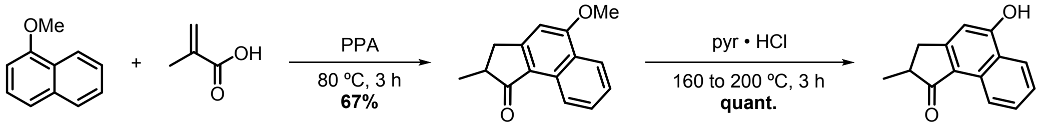

1 **2**

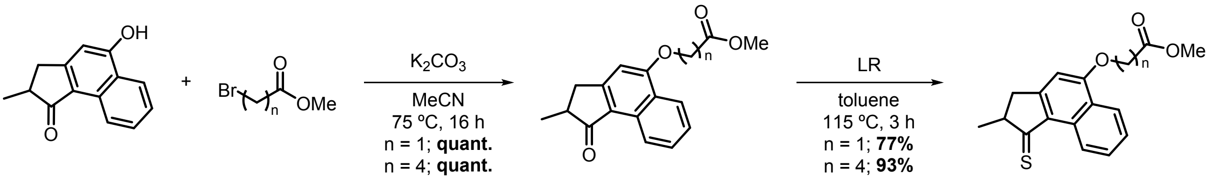

2

3a; n = 1
3b; n = 4

4a; n = 1
4b; n = 4

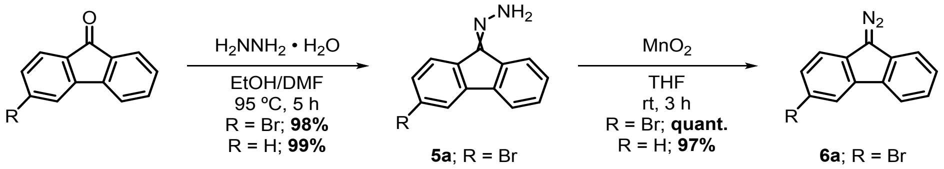

5a; R = Br
5b; R = H

6a; R = Br
6b; R = H

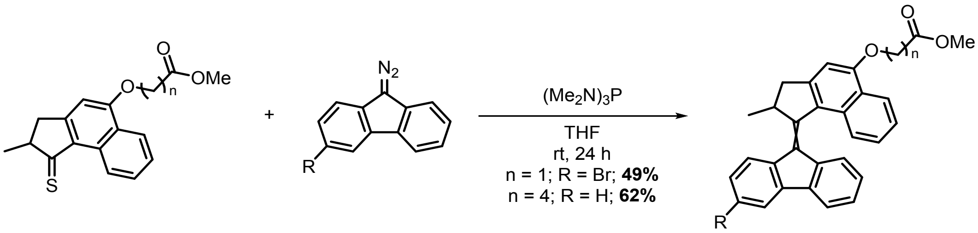

4a; n = 1
4b; n = 4

6a; R = Br
6b; R = H

7a; n = 1; R = Br
7b; n = 4; R = H

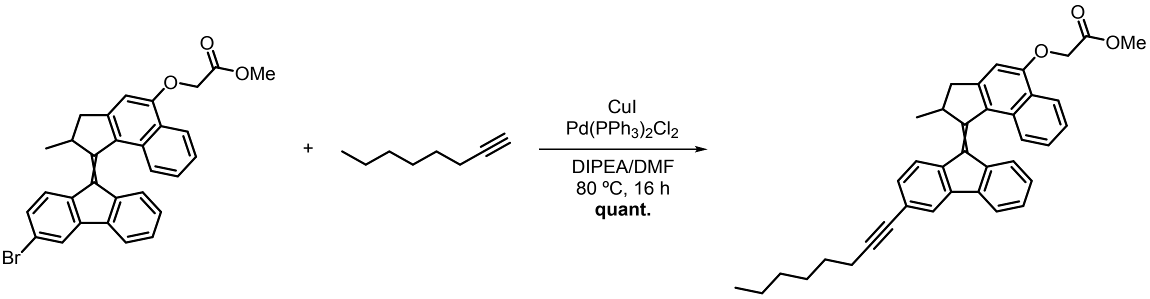

7a **8**

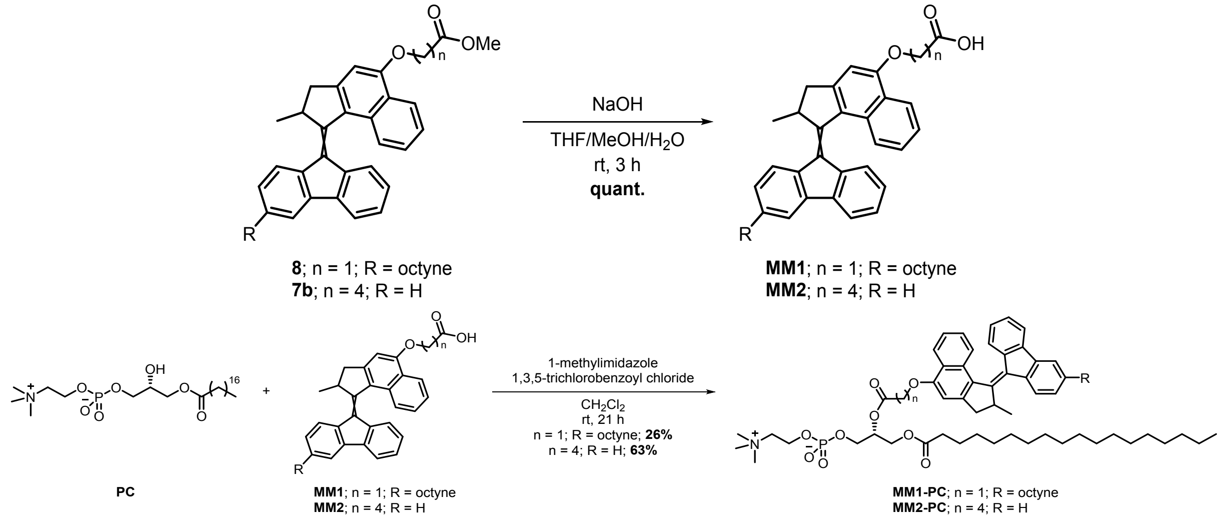

Scheme S1: Overview of schemes for the synthesis of **MM1-PC** and **MM2-PC**.

(*R*)-2-hydroxy-3-(stearoyloxy)propyl (2-(trimethylammonio)ethyl) phosphate (PC)[1]

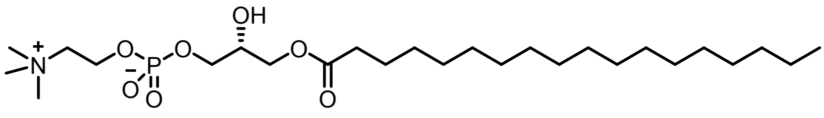

PC
$C_{26}H_{54}NO_7P$
Mw = 523.69 g/mol

A flame dried round bottom three-neck flask equipped with a stirring egg and a reflux condenser was charged with (*R*)-2,3-dihydroxy-propyl (2-(trimethylammonio)ethyl) phosphate (489 mg, 1.90 mmol, 1.0 eq.), Bu_2SnO (525 mg, 2.11 mmol, 1.1 eq.) and *i*PrOH (25 mL). The reaction mixture was stirred for 6 h at 105 °C before cooling it to 0 °C (ice/water) and adding Et_3N (0.3 mL, 218 mg, 2.16 mmol, 1.1 eq.) and stearoyl chloride (0.7 mL, 628 mg, 2.07 mmol, 1.1 eq.). The reaction was allowed to warm up to room temperature and the mixture stirred for 16 h, the solvent removed under reduced pressure and the crude purified *via* flash column chromatography (with a gradient of CH_2Cl_2:MeOH:H_2O = 9:1:0 to 10:4:0.5 to 10:8:2), yielding **PC** as a colorless, sticky gum (572 mg, 1.09 mmol, 58%).

^{1}H NMR (400 MHz, CD_3OD): δ = 4.34 – 4.27 (m, 2H), 4.18 (dd, *J* = 11.3, 4.5 Hz, 1H), 4.11 (dd, *J* = 11.3, 6.1 Hz, 1H), 4.03 – 3.94 (m, 1H), 3.94 – 3.86 (m, 2H), 3.68 – 3.63 (m, 2H), 3.24 (s, 9H), 2.35 (t, *J* = 7.5 Hz, 2H), 1.62 (p, *J* = 7.2 Hz, 2H), 1.29 (s, 28H), 0.90 (t, *J* = 6.8 Hz, 3H).
^{13}C{^{1}H} NMR (101 MHz, CD_3OD): δ = 175.3, 69.8 (d), 67.8 (d), 66.2, 60.4 (d), 54.7 (t), 34.9, 33.1, 30.8, 30.8, 30.6, 30.5, 30.6, 30.3, 26.0, 23.7, 14.5.
^{31}P NMR (162 MHz, CD_3OD): δ = -0.38.
HRMS-ESI (ESI+): calculated for $C_{26}H_{54}NO_7PH^+$ $[M+H]^+$ 524.3711, found 524.3703.

5-methoxy-2-methyl-2,3-dihydro-1*H*-cyclopenta[*a*]naphthalen-1-one (1)[2]

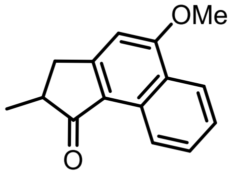

1

$C_{15}H_{14}O_2$

Mw = 226.28 g/mol

A round bottom flask was charged with PPA 115% (~40 mL) and heated to 80 °C. 1-Methoxynaphthalene (6.5 mL, 7.12 g, 45.0 mmol, 1.0 eq.) was added and the mixture mechanically stirred with an overhead stirrer. After mixing occurred, methacrylic acid (6.5 mL, 6.63 g, 77.0 mmol, 1.7 eq.) was added and the mixture stirred at 80 °C for 3 h. The mixture was quenched on ice/water, left stirring for 16 h and extracted with EtOAc. The organic layer was washed with H_2O and aqueous $NaHCO_3$, dried over $MgSO_4$ and the solvent removed under reduced pressure, yielding **1** as yellow/gold solid (6.79 g, 30.0 mmol, 67%).

^{1}**H NMR** (600 MHz, $CDCl_3$): δ = 9.13 (d, *J* = 8.3 Hz, 1H), 8.25 (d, *J* = 8.4 Hz, 1H), 7.66 (ddd, *J* = 8.3, 6.8, 1.3 Hz, 1H), 7.52 (ddd, *J* = 8.3, 6.9, 1.3 Hz, 1H), 6.78 (s, 1H), 4.08 (s, 3H), 3.42 (dd, *J* = 18.0, 8.0 Hz, 1H), 2.81 – 2.73 (m, 2H), 1.36 (d, *J* = 7.3 Hz, 3H).

^{13}**C{^{1}H} NMR** (151 MHz, $CDCl_3$): δ = 208.5, 161.8, 159.3, 130.8, 129.4, 126.1, 125.3, 124.0, 123.6, 122.6, 101.6, 56.1, 42.3, 36.0, 17.1.

HRMS-ESI (APCI+): calculated for $C_{15}H_{14}O_2H^+$ $[M+H]^+$ 227.1067, found 227.1067.

5-hydroxy-2-methyl-2,3-dihydro-1*H*-cyclopenta[*a*]naphthalen-1-one (2)[3]

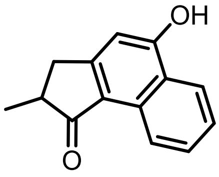

2

$C_{14}H_{12}O_2$

Mw = 212.25 g/mol

A round bottom flask equipped with a stirring egg was charged with pyridine HCl (53.5 g, 463 mmol, 29.9 eq.) and heated to 180 °C. Compound **1** (3.50 g, 15.5 mmol, 1.0 eq.) was added and the resulting mixture heated to 210 °C for 3 h. The reaction mixture was poured onto ice/water, extracted with EtOAc, washed with H_2O (5x) and the solvent removed under reduced pressure, yielding **2** as a pale brown solid (3.28 g, 15.5 mmol, quant.).

^{1}**H NMR** (600 MHz, $(CD_3)_2SO$): δ = 11.41 (s, 1H), 8.96 (d, *J* = 8.3 Hz, 1H), 8.20 (d, *J* = 8.3 Hz, 1H), 7.67 (ddd, *J* = 8.3, 6.9, 1.3 Hz, 1H), 7.53 (ddd, *J* = 8.3, 6.9, 1.3 Hz, 1H), 6.92 (s, 1H), 3.37 (dd, *J* = 17.6, 7.6 Hz, 1H), 2.73 – 2.65 (m, 2H), 1.21 (d, *J* = 7.4 Hz, 3H).

^{13}**C{^{1}H} NMR** (151 MHz, $(CD_3)_2SO$): δ = 207.0, 160.4, 159.8, 130.5, 129.2, 125.4, 124.1, 122.9, 122.8, 121.1, 105.7, 41.4, 34.9, 16.6.

HRMS-ESI (ESI-): calculated for $C_{14}H_{11}O_2^-$ $[M-H]^-$ 211.0765, found 211.0764.

Methyl 2-((2-methyl-1-oxo-2,3-dihydro-1*H*-cyclopenta[*a*]naphthalen-5-yl)oxy)acetate (3a)

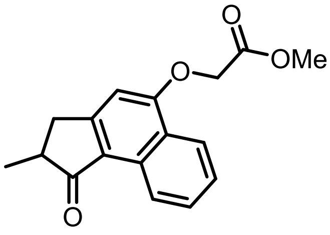

3a
$C_{17}H_{16}O_4$
Mw = 284.31 g/mol

An oven dried crimp top vial equipped with a stirring bar was charged with **2** (1.04 g, 4.90 mmol, 1.0 eq.) and K_2CO_3 (0.86 g, 6.24 mmol, 1.25 eq). The vial was crimped, flushed with nitrogen and methyl 2-bromoacetate (0.88 mL, 1.09 g, 7.11 mmol, 1.45 eq.) and dry MeCN (25 mL) were added. The reaction mixture was heated to 75 ºC for 16 h, followed by heating to 85 ºC for 1 h. The reaction mixture was allowed to cool down to room temperature, H_2O added and extracted with CH_2Cl_2 (3x). The combined organic layers were dried over Na_2SO_4, the solvent removed under reduced pressure and the crude compound purified *via* flash column chromatography (gradient *n*-pentane to EtOAc), yielding **3a** as an off-white solid (1.39 g, 4.89 mmol, quant.).

^{1}H NMR (400 MHz, $CDCl_3$): δ = 9.13 (d, *J* = 8.3 Hz, 1H), 8.35 (d, *J* = 8.3 Hz, 1H), 7.69 (ddd, *J* = 8.3, 6.9, 1.3 Hz, 1H), 7.56 (ddd, J = 8.3, 6.9, 1.3 Hz, 1H), 6.65 (s, 1H), 4.90 (s, 2H), 3.86 (s, 3H), 3.40 (dd, *J* = 17.7, 7.7 Hz, 1H), 2.84 – 2.71 (m, 2H), 1.35 (d, *J* = 7.3 Hz, 3H).
^{13}C{^{1}H} NMR (101 MHz, $CDCl_3$): δ = 208.5, 168.6, 159.7, 158.6, 130.8, 129.7, 126.4, 125.2, 124.6, 124.0, 122.7, 102.4, 65.6, 52.7, 42.4, 35.9, 16.9.
HRMS-ESI (ESI+): calculated for $C_{17}H_{16}O_4H^+$ $[M+H]^+$ 285.1121, found 285.1118.

Methyl 5-((2-methyl-1-oxo-2,3-dihydro-1*H*-cyclopenta[*a*]naphthalen-5-yl)oxy) penta-noate (3b)

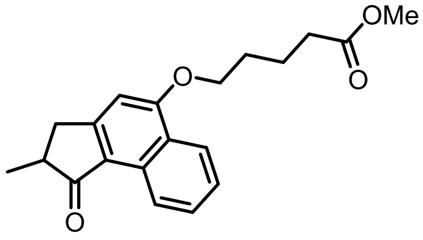

3b
$C_{20}H_{22}O_4$
Mw = 326.39 g/mol

An oven dried crimp top vial equipped with a stirring bar was charged with **2** (996 mg, 4.69 mmol, 1.0 eq.) and K_2CO_3 (849 mg, 6.14 mmol, 1.3 eq.). The vial was crimped, flushed with nitrogen and methyl 5-bromopentanoate (1.00 mL, 1.37 g, 7.04 mmol, 1.5 eq.) and dry MeCN (23 mL) was added. The reaction mixture was heated to 75 ºC for 16 h, followed by heating to 85 ºC for 1 h. The reaction mixture was allowed to cool down to room temperature, H_2O added and extracted with CH_2Cl_2 (3x). The combined organic layers were dried over Na_2SO_4, the solvent removed under reduced pressure and the crude compound purified *via* flash column chromatography (gradient *n*-pentane to EtOAc), yielding **3b** as a brown solid (1.53 g, 4.69 mmol, quant.).

^{1}H NMR (400 MHz, $CDCl_3$): δ = 9.12 (d, *J* = 8.4 Hz, 1H), 8.26 (d, *J* = 8.3 Hz, 1H), 7.66 (ddd, *J* = 8.3, 6.9, 1.3 Hz, 1H), 7.52 (ddd, *J* = 8.3, 6.9, 1.3 Hz, 1H), 6.76 (s, 1H), 4.24 (t, *J* = 5.9 Hz,

2H), 3.69 (s, 3H), 3.42 (dd, *J* = 18.0, 8.0 Hz, 1H), 2.81 – 2.72 (m, 2H), 2.48 (t, *J* = 7.1 Hz, 2H), 2.07 – 1.89 (m, 4H), 1.35 (d, *J* = 7.3 Hz, 3H).

^{13}C{^{1}H} NMR (151 MHz, $CDCl_3$): δ = 208.5, 173.9, 161.0, 159.3, 130.8, 129.4, 126.1, 125.3, 124.0, 123.5, 122.6, 102.2, 68.3, 51.8, 42.3, 36.0, 33.8, 28.6, 21.9, 17.1.

HRMS-ESI (ESI+): calculated for $C_{20}H_{22}O_4H^+$ $[M+H]^+$ 327.1591, found 327.1990.

Methyl 2-((2-methyl-1-thioxo-2,3-dihydro-1*H*-cyclopenta[*a*]naphthalen-5-yl)oxy)acetate (4a)

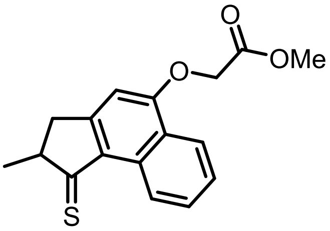

4a
$C_{17}H_{16}O_3S$
Mw = 300.37 g/mol

An oven dried crimp top vial equipped with a stirring bar was charged with **3a** (812 mg, 2.86 mmol, 1.0 eq.) and Lawesson's reagent (1.33 g, 3.28 mmol, 1.15 eq.). The vial was crimped, flushed with nitrogen and dry toluene (28 mL) was added and the reaction mixture was heated to 115 °C for 3 h. The reaction mixture was allowed to cool down to room temperature, concentrated and the crude purified *via* flash column chromatography (inert atmosphere, gradient *n*-pentane to CH_2Cl_2), yielding **4a** as a purple/red solid (658 mg, 2.19 mmol, 77%), which was immediately further reacted.

^{1}H NMR (600 MHz, $CDCl_3$): δ = 10.17 (d, *J* = 8.6 Hz, 1H), 8.42 (d, *J* = 8.4 Hz, 1H), 7.76 (ddd, *J* = 8.4, 6.9, 1.4 Hz, 1H), 7.59 (ddd, *J* = 8.3, 6.9, 1.3 Hz, 1H), 6.71 (s, 1H), 4.93 (s, 2H), 3.87 (s, 3H), 3.47 (dd, *J* = 17.9, 6.6 Hz, 1H), 3.14 (pd, *J* = 7.3, 2.4 Hz, 1H), 2.88 (dd, *J* = 17.9, 2.4 Hz, 1H), 1.49 (d, *J* = 7.3 Hz, 3H).

^{13}C{^{1}H} NMR (151 MHz, $CDCl_3$): δ = 168.4, 161.3, 159.9, 134.5, 131.7, 131.1, 126.7, 125.5, 124.4, 123.0, 102.1, 65.6, 55.2, 52.7, 40.5, 22.1.

HRMS-ESI (ESI+): calculated for $C_{17}H_{16}O_3SH^+$ $[M+H]^+$ 301.0893, found 301.0895.

Methyl 5-((2-methyl-1-thioxo-2,3-dihydro-1*H*-cyclopenta[*a*]naphthalen-5-yl)oxy) penta-noate (4b)

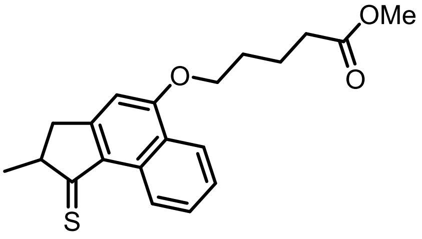

4b
$C_{20}H_{22}O_3S$
Mw = 342.45 g/mol

An oven dried crimp top vial equipped with a stirring bar was charged with **3b** (851 mg, 2.61 mmol, 1.0 eq.) and Lawesson's reagent (1.18 g, 2.92 mmol, 1.1 eq.). The vial was crimped, flushed with nitrogen and dry toluene (25 mL) was added and the reaction mixture was heated to 115 °C for 3 h. The reaction mixture was allowed to cool down to room temperature, concentrated and the crude purified *via* flash column chromatography (inert atmosphere, gradient

n-pentane to CH_2Cl_2), yielding **4b** as a purple/red oil (830 mg, 2.42 mmol, 93%), which was immediately further reacted.

^{1}H NMR (400 MHz, $CDCl_3$): δ = 10.19 (d, *J* = 8.4 Hz, 1H), 8.32 (d, *J* = 8.5 Hz, 1H), 7.74 (ddd, *J* = 8.4, 6.9, 1.4 Hz, 1H), 7.55 (ddd, *J* = 8.2, 6.9, 1.2 Hz, 1H), 6.81 (s, 1H), 4.28 (t, *J* = 6.0 Hz, 2H), 3.69 (s, 3H), 3.47 (dd, *J* = 18.1, 6.6 Hz, 1H), 3.15 (pd, *J* = 7.1, 2.2 Hz, 1H), 2.88 (dd, *J* = 18.2, 2.3 Hz, 1H), 2.48 (t, *J* = 7.1 Hz, 2H), 2.08 – 1.90 (m, 4H), 1.49 (d, *J* = 7.2 Hz, 3H).
^{13}C{^{1}H} NMR (151 MHz, $CDCl_3$): δ = 173.9, 162.2, 161.5, 133.8, 131.7, 130.8, 126.5, 125.6, 124.4, 122.8, 101.9, 68.5, 55.0, 51.8, 40.5, 33.7, 28.6, 22.2, 21.9.
HRMS-ESI (ESI+): calculated for $C_{20}H_{22}O_3SH^+$ $[M+H]^+$ 343.1362, found 343.1359.

(3-bromo-9*H*-fluoren-9-ylidene)hydrazine (5a)

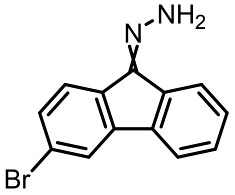

5a
$C_{13}H_9BrN_2$
Mw = 273.13 g/mol

A round bottom flask equipped with a stirring egg and a reflux condenser was charged with 3-bromo-9*H*-fluoren-9-one (0.97 g, 3.74 mmol, 1.0 eq.), dry EtOH (60 mL), DMF (0.1 mL) and hydrazine monohydrate (50 – 60%, 3.3 mL, 3.41 g, 37.5 mmol, 10 eq.). The resulting reaction mixture was heated to 95 °C and stirred for 5 h. Subsequently, the reaction mixture was allowed to cool down to room temperature and the solvents removed under reduced pressure, yielding **5a** as an off-white solid (*E*/*Z* mixture; 1.00 g, 3.67 mmol, 98%).

^{1}H NMR (400 MHz, $CDCl_3$): δ = 7.92 – 7.84 (m, 1H), 7.79 – 7.68 (m, 2H), 7.63 – 7.55 (m, 1H), 7.49 – 7.30 (m, 3H), 6.46 (s, 1H), 6.38 (s, 1H).
^{13}C{^{1}H} NMR (101 MHz, $CDCl_3$): δ = 144.6, 144.3, 143.4, 140.3, 140.0, 138.2, 137.4, 136.6, 130.8, 130.4, 130.4, 129.9, 128.9, 128.8, 128.7, 128.5, 126.5, 125.5, 124.1, 124.0, 122.9, 122.5, 122.3, 121.0, 120.9, 119.9.
HRMS-ESI (ESI+): calculated for $C_{13}H_9BrN_2H^+$ $[M+H]^+$ 273.0022, found 273.0019.

(9*H*-fluoren-9-ylidene)hydrazine (5b)[4]

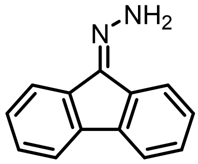

5b
$C_{13}H_{10}N_2$
Mw = 194.24 g/mol

A round bottom flask equipped with a stirring egg and a reflux condenser was charged with 9*H*-fluoren-9-one (1.04 g, 5.74 mmol, 1.0 eq.), dry EtOH (65 mL), DMF (0.1 mL) and hydrazine monohydrate (50 – 60%, 3.4 mL, 3.51 g, 38.6 mmol, 6.5 eq.). The resulting reaction mixture was heated to 95 ºC and stirred for 5 h. Subsequently, the reaction mixture was allowed to cool down to room temperature and the solvents removed under reduced pressure, yielding **5b** as a pale yellow solid (1.10 g, 5.66 mmol, 99%).

^{1}H NMR (400 MHz, $CDCl_3$): δ = 7.90 (d, *J* = 7.6 Hz, 1H), 7.75 (t, *J* = 7.3 Hz, 2H), 7.65 (d, *J* = 6.9 Hz, 1H), 7.44 (t, *J* = 7.5 Hz, 1H), 7.39 – 7.28 (m, 3H), 6.41 (s, 2H).
^{13}C{^{1}H} NMR (151 MHz, $CDCl_3$): δ = 145.7, 141.4, 138.7, 137.9, 130.4, 129.8, 128.6, 128.1, 127.8, 125.6, 120.9, 120.6, 119.7.
HRMS-ESI (ESI+): calculated for $C_{13}H_{10}N_2H^+$ $[M+H]^+$ 195.0917, found 195.0915.

3-bromo-9-diazo-9*H*-fluorene (6a)

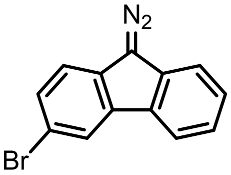

6a
$C_{13}H_7BrN_2$
Mw = 271.12 g/mol

Under light exclusion, a flame dried Schlenk tube equipped with a stirring egg was charged **5a** (556 mg, 2.04 mmol, 1.0 eq.) and dry THF (20 mL). The reaction mixture was cooled down to 0 ºC (ice/water), MnO_2 (activated, 810 mg, 4.5 eq.) added and the reaction mixture stirred for 5 min at 0 ºC and then 3 h at room temperature. The reaction mixture was filtered over celite with CH_2Cl_2 and the solvents removed under reduced pressure to yield **6a** as a red oil/solid (551 mg, 2.03 mmol, quant.), which was immediately further reacted.

^{1}H NMR (600 MHz, $CDCl_3$): δ = 8.06 (d, *J* = 1.8 Hz, 1H), 7.91 (dt, *J* = 7.7, 0.9 Hz, 1H), 7.51 (dt, *J* = 7.7, 0.9 Hz, 1H), 7.49 (dd, *J* = 8.2, 1.8 Hz, 1H), 7.42 (td, *J* = 7.6, 1.1 Hz, 1H), 7.38 (d, *J* = 8.3 Hz, 1H), 7.34 (td, *J* = 7.5, 1.1 Hz, 1H).
^{13}C{^{1}H} NMR (151 MHz, $CDCl_3$): δ = 133.4, 133.2, 131.7, 130.4, 129.2, 127.2, 124.9, 124.3, 121.4, 120.6, 119.5, 118.2.

9-diazo-9*H*-fluorene (6b)[4]

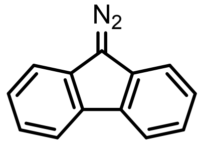

6b
$C_{13}H_8N_2$
Mw = 192.22 g/mol

Under light exclusion, a flame dried Schlenk tube equipped with a stirring egg was charged **5b** (219 mg, 1.13 mmol, 1.0 eq.) and dry THF (11 mL). The reaction mixture was cooled down to 0 °C (ice/water), MnO_2 (activated, 422 mg, 4.86 mmol, 4.25 eq.) added and the reaction mixture stirred for 5 min at 0 °C and then 3 h at room temperature. The reaction mixture was filtered over celite with CH_2Cl_2 and the solvents removed under reduced pressure to yield **6b** as a red solid (210 mg, 1.09 mmol, 97%), which was immediately further reacted.

^{1}**H NMR** (400 MHz, $CDCl_3$): δ = 7.96 (d, *J* = 7.6 Hz, 2H), 7.52 (d, *J* = 7.6 Hz, 2H), 7.40 (td, *J* = 7.5, 1.2 Hz, 2H), 7.33 (td, *J* = 7.5, 1.2 Hz, 2H).
^{13}**C{^{1}H} NMR** (101 MHz, $CDCl_3$): δ = 133.1, 131.6, 126.5, 124.7, 121.1, 119.5.

Methyl 2-((1-(3-bromo-9*H*-fluoren-9-ylidene)-2-methyl-2,3-dihydro-1*H*-cyclopenta[*a*]naphthalen-5-yl)oxy)acetate (7a)

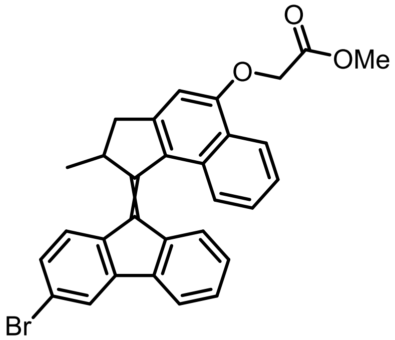

7a
$C_{30}H_{23}BrO_3$
Mw = 511.42 g/mol

An oven-dried crimp top vial equipped with a stirring bar was charged with **4a** (658 mg, 2.19 mmol, 1.1 eq.) and **6a** (551 mg, 2.03 mmol, 1.0 eq.) dissolved in dry THF (15 mL) and stirred for 24 h at room temperature under a nitrogen atmosphere. Subsequently, hexamethylphosphorous triamide (1.5 mL, 1.33 g, 8.13 mmol, 4.0 eq.) was added and the reaction mixture stirred for another 24 h at room temperature. The solvents were removed under reduced pressure, and the crude product purified *via* flash column chromatography (gradient *n*-pentane to CH_2Cl_2), yielding **7b** as a yellow solid (*E*/*Z* mixture, 511 mg, 1.00 mmol, 49%).

^{1}**H NMR** (600 MHz, $CDCl_3$): δ = 8.46 (d, *J* = 8.5 Hz, 1H), 7.97 (d, *J* = 7.7 Hz, 1H), 7.88 (s, 0.5H), 7.81 (d, *J* = 7.6 Hz, 1H), 7.72 (d, *J* = 7.5 Hz, 0.5H), 7.66 (dd, *J* = 14.7, 8.4 Hz, 1H), 7.52 – 7.46 (m, 1.5H), 7.45 – 7.33 (m, 2H), 7.21 (t, *J* = 7.4 Hz, 0.5H), 6.91 (d, *J* = 8.5 Hz, 0.5H), 6.87 – 6.82 (m, 1.5H), 6.71 (d, *J* = 8.0 Hz, 0.5H), 6.55 (d, J = 8.6 Hz, 0.5H), 4.93 (s, 2H), 4.30 (p, *J* = 6.5 Hz, 0.5H), 4.24 (p, *J* = 6.6 Hz, 0.5H), 3.89 (s, 3H), 3.55 (dd, *J* = 15.0, 5.4 Hz, 1H), 2.72 (d, *J* = 15.2 Hz, 1H), 1.38 (t, *J* = 6.7 Hz, 3H).
^{13}**C{^{1}H} NMR** (151 MHz, $CDCl_3$): δ = 169.1, 156.4, 152.3, 152.2, 149.1, 149.0, 141.8, 141.3, 140.2, 138.7, 138.6, 138.1, 137.5, 136.0, 130.7, 130.6, 129.7, 129.6, 129.5, 128.6, 128.0,

127.9, 127.8, 127.7, 127.6, 127.4, 127.2, 127.1, 126.8, 126.8, 126.6, 125.9, 125.4, 125.3, 125.2, 124.8, 124.0, 123.2, 123.1, 122.9, 122.1, 120.6, 120.5, 120.0, 119.3, 103.6, 103.6, 65.8, 52.6, 45.3, 45.3, 42.6, 19.7, 19.6.

HRMS-ESI (ESI-): calculated for $C_{30}H_{22}BrO_3^-$ $[M]^-$ 509.0758, found 509.0761.

Methyl 5-((1-(9*H*-fluoren-9-ylidene)-2-methyl-2,3-dihydro-1*H*-cyclopenta[*a*]naphthalen-5-yl)oxy)pentanoate (7b)

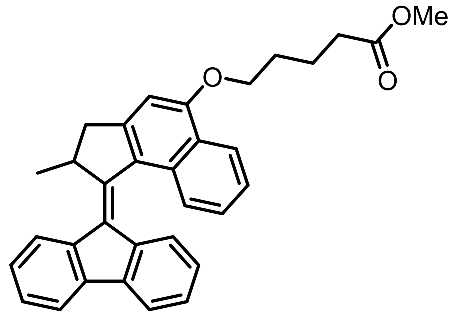

7b
$C_{33}H_{30}O_3$
Mw = 474.60 g/mol

An oven-dried crimp top vial equipped with a stirring bar was charged with **4b** (370 mg, 1.08 mmol, 1.0 eq.) and **6b** (260 mg, 1.35 mmol, 1.25 eq.) dissolved in dry THF (8 mL) and stirred for 24 h at room temperature under a nitrogen atmosphere. Subsequently, hexamethylphosphorous triamide (1.2 mL, 1.09 g, 6.70 mmol, 6.25 eq.) was added and the reaction mixture stirred for another 24 h at room temperature. The solvents were removed under reduced pressure, and the crude product purified *via* flash column chromatography (gradient *n*-pentane to CH_2Cl_2), yielding **7b** as a yellow solid (319 mg, 0.67 mmol, 62%).

^{1}H NMR (400 MHz, $CDCl_3$): δ = 8.37 (d, *J* = 8.3 Hz, 1H), 8.01 – 7.96 (m, 1H), 7.89 – 7.84 (m, 1H), 7.78 (d, *J* = 7.5 Hz, 1H), 7.71 (d, *J* = 8.4 Hz, 1H), 7.49 – 7.43 (m, 1H), 7.41 – 7.37 (m, 2H), 7.37 – 7.31 (m, 1H), 7.20 (td, *J* = 7.4, 1.1 Hz, 1H), 6.93 (s, 1H), 6.84 – 6.78 (m, 1H), 6.74 (d, *J* = 7.9 Hz, 1H), 4.36 – 4.22 (m, 3H), 3.71 (s, 3H), 3.55 (dd, *J* = 15.1, 5.7 Hz, 1H), 2.72 (d, *J* = 15.1 Hz, 1H), 2.51 (t, *J* = 7.0 Hz, 2H), 2.09 – 1.97 (m, 4H), 1.40 (d, *J* = 6.7 Hz, 3H).

$^{13}C\{^1H\}$ **NMR** (101 MHz, $CDCl_3$): δ = 174.0, 157.4, 151.8, 149.3, 140.1, 139.9, 139.3, 137.4, 130.7, 128.7, 128.3, 127.5, 127.3, 126.9, 126.5, 126.4, 125.9, 125.8, 124.8, 124.8, 123.9, 122.9, 119.8, 119.0, 103.3, 68.0, 51.7, 45.3, 42.7, 33.9, 28.9, 22.0, 19.8.

HRMS-ESI (APCI+): calculated for $C_{33}H_{30}O_3H^+$ $[M+H]^+$ 475.2268, found 475.2263.

Methyl 2-((2-methyl-1-(3-(oct-1-yn-1-yl)-9*H*-fluoren-9-ylidene)-2,3-dihydro-1*H*-cyclopenta[*a*]naphthalen-5-yl)oxy)acetate (8)

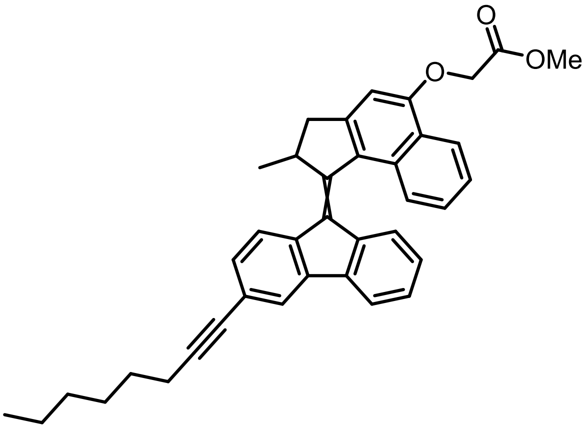

8
$C_{38}H_{36}O_3$
Mw = 540.70 g/mol

A crimp top vial equipped with a stirring bar was charged with **7a** (52.5 mg, 0.10 mmol, 1.0 eq.), $Pd(PPh_3)_2Cl_2$ (8.70 mg, 10 mol%) and CuI (3.80 mg, 0.02 mmol, 20 mol%). The vial was crimped, flushed with nitrogen and a mixture of DMF/DIPEA (1:1, freeze-pump-thawed, 3 mL) and 1-octyne (0.05 mL, 37.4 mg, 0.34 mmol, 3.25 eq.) were added. The resulting reaction mixture was heated to 80 °C and stirred for 16 h. Afterwards, the reaction mixture was allowed to cool down to room temperature, the solvents were removed under reduced pressure, and the crude product purified *via* flash column chromatography (gradient *n*-pentane to CH_2Cl_2), yielding **8** as an orange solid (*E*/*Z* mixture, 53.0 mg, 0.09 mmol, 96%).

^1H **NMR** (600 MHz, $CDCl_3$): δ = 8.50 – 8.46 (m, 1H), 7.99 (t, *J* = 7.1 Hz, 0.5H), 7.92 – 7.88 (m, 1H), 7.87 – 7.82 (m, 1H), 7.77 (d, *J* = 7.7 Hz, 0.5H), 7.76 – 7.67 (m, 1H), 7.53 – 7.49 (m, 1.5H), 7.43 – 7.37 (m, 2H), 7.23 (t, *J* = 7.4 Hz, 0,5H), 6.89 – 6.83 (m, 2H), 6.75 – 6.72 (m, 0.5H), 6.65 – 6.57 (m, 0.5H), 4.96 (s, 2H), 4.36 – 4.26 (m, 1H), 3.92 (s, 3H), 3.57 (dt, *J* = 15.0, 5.3 Hz, 1H), 2.77 – 2.71 (m, 1H), 2.53 – 2.37 (m, 2H), 1.73 – 1.59 (m, 2H), 1.58 – 1.44 (m, 3H), 1.43 – 1.38 (m, 4H), 1.38 – 1.34 (m, 2H), 0.98 – 0.89 (m, 3H).

$^{13}C\{^1H\}$ **NMR** (101 MHz, $CDCl_3$): δ = 169.2, 156.3, 151.9, 151.8, 148.9, 148.9, 140.2, 139.9, 139.4, 139.3, 139.1, 138.8, 137.6, 136.5, 130.8, 130.7, 130.2, 129.9, 129.9, 129.3, 128.6, 128.5, 127.7, 127.6, 127.5, 127.4, 127.2, 126.7, 126.7, 126.2, 125.8, 125.6, 125.3, 124.7, 123.9, 123.7, 123.1, 122.8, 122.1, 122.0, 121.8, 119.9, 119.1, 103.7 – 103.6 (m), 91.0, 90.7, 81.5, 81.45, 65.9, 65.8, 52.6, 45.3, 45.2, 42.5, 32.0 – 31.8 (m), 31.6, 31.5, 29.0, 29.0, 28.9, 28.8, 22.9 – 22.6 (m), 19.8, 19.7, 14.3 – 14.1 (m).

HRMS-ESI (ESI+): calculated for $C_{38}H_{36}O_3H^+$ $[M+H]^+$ 541.2737, found 541.2744.

2-((2-methyl-1-(3-(oct-1-yn-1-yl)-9*H*-fluoren-9-ylidene)-2,3-dihydro-1*H*-cyclopenta[*a*] naphthalen-5-yl)oxy)acetic acid (MM1)

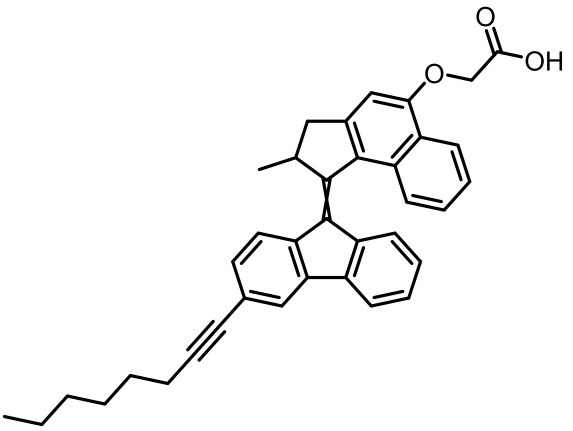

MM1
$C_{37}H_{34}O_3$
Mw = 526.68 g/mol

A crimp top vial equipped with a stirring bar was charged with **8** (53.0 mg, 0.10 mmol, 1.0 eq.). The vial was crimped, flushed with nitrogen and THF (1.8 mL), MeOH (1.4 mL) and $NaOH_{aq}$ (0.1M, 19.6 mg in 0.8 mL, 5.0 eq.) were added and the reaction mixture stirred for 3 h at room temperature. The pH of the reaction mixture was adjusted to 3, extracted with CH_2Cl_2 (2x), dried over Na_2SO_4 and the solvent removed under reduced pressure, yielding **MM1** as an orange solid (*E*/*Z* mixture, 51.5 mg, 0.10 mmol, quant.).

^{1}H NMR (400 MHz, CD_2Cl_2): δ = 8.43 (d, *J* = 8.4 Hz, 1H), 8.00 – 7.95 (m, 0.5H), 7.91 – 7.87 (m, 0.5H), 7.86 – 7.82 (m, 0.5H), 7.81 – 7.65 (m, 2.5H), 7.54 – 7.46 (m, 1.5H), 7.42 – 7.34 (m, 2H), 7.21 (t, *J* = 7.4 Hz, 0.5H), 6.93 (s, 1H), 6.84 – 6.78 (m, 1H), 6.70 (d, *J* = 7.8 Hz, 0.5H), 6.61 (d, *J* = 8.2 Hz, 0.5H), 5.00 (s, 2H), 4.38 – 4.22 (m, 1H), 3.60 – 3.50 (m, 1H), 2.74 (d, *J* = 15.3 Hz, 1H), 2.52 – 2.38 (m, 2H), 1.71 – 1.57 (m, 2H), 1.41 – 1.31 (m, 9H), 0.96 – 0.90 (m, 3H).
^{13}C{^{1}H} NMR (101 MHz, CD_2Cl_2): δ = 172.7, 156.3, 152.5, 152.5, 149.6, 149.6, 140.5, 140.0, 139.6, 139.4, 139.0, 137.9, 136.8, 132.9, 132.9, 132.6, 132.5, 131.0, 131.0, 130.4, 130.1, 129.8, 129.3, 129.2, 129.1, 128.7, 128.7, 127.9, 127.8, 127.7, 127.6, 127.6, 127.0, 127.0, 126.5, 126.0, 125.8, 125.6, 124.9, 124.3, 124.1, 123.1, 122.9, 122.4, 122.3, 122.2, 120.1, 119.4, 104.3 – 104.2 (m), 91.4, 91.2, 81.4, 65.7, 45.7, 45.6, 42.8, 32.2, 31.9, 31.8, 30.1, 29.3, 29.3, 29.1, 29.1, 23.33 – 22.83 (m), 19.9, 19.8, 19.8, 19.7, 14.3, 14.3.
HRMS-ESI (ESI-): calculated for $C_{37}H_{33}O_3^-$ [M-H]$^-$ 525.2435, found 525.2433.

5-((1-(9*H*-fluoren-9-ylidene)-2-methyl-2,3-dihydro-1*H*-cyclopenta[*a*]naphthalen-5-yl)oxy) pentanoic acid (MM2)

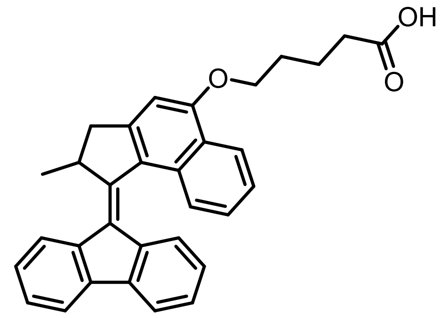

MM2
$C_{32}H_{28}O_3$
Mw = 460.57 g/mol

A crimp top vial equipped with a stirring bar was charged with **7b** (50.7 mg, 0.11 mmol, 1.0 eq.). The vial was crimped, flushed with nitrogen and THF (3.6 mL), MeOH (2.6 mL) and $NaOH_{aq}$ (0.1M, 21.4 mg in 0.85 mL, 5.0 eq.) were added and the reaction mixture stirred for 3 h at room temperature. The pH of the reaction mixture was adjusted to 3 (1M HCl_{aq}), extracted with CH_2Cl_2 (2x), dried over Na_2SO_4 and the solvent removed under reduced pressure, yielding **MM2** as a yellow to orange solid (48.5 mg, 0.11 mmol, quant.).

^{1}H NMR (400 MHz, CD_2Cl_2): δ = 8.38 (d, *J* = 8.5 Hz, 1H), 8.02 – 7.97 (m, 1H), 7.89 – 7.85 (m, 1H), 7.78 (d, *J* = 7.2 Hz, 1H), 7.67 (d, *J* = 8.4 Hz, 1H), 7.49 – 7.42 (m, 1H), 7.42 – 7.36 (m, 2H), 7.35 – 7.30 (m, 1H), 7.20 (td, *J* = 7.4, 1.1 Hz, 1H), 6.99 (s, 1H), 6.84 – 6.77 (m, 1H), 6.71 (d, *J* = 7.9 Hz, 1H), 4.33 – 4.25 (m, 3H), 3.56 (dd, *J* = 15.2, 5.6 Hz, 1H), 2.74 (d, *J* = 15.2 Hz, 1H), 2.56 (t, *J* = 7.1 Hz, 2H), 2.12 – 1.95 (m, 4H), 1.39 (d, *J* = 6.6 Hz, 3H).
^{13}C{^{1}H} NMR (101 MHz, CD_2Cl_2): δ = 157.8, 152.5, 150.0, 140.3, 139.9, 139.5, 137.7, 131.0, 128.7, 128.4, 127.6, 127.4, 127.2, 126.7, 126.6, 126.1, 125.9, 125.1, 125.0, 124.2, 123.2, 119.9, 119.2, 103.8, 68.4, 45.6, 42.8, 29.0, 22.1, 19.8.
HRMS-ESI (APCI+): calculated for $C_{32}H_{28}O_3H^+$ $[M+H]^+$ 461.2111, found 461.2112.

(2*R*)-2-(2-((2-methyl-1-((*E*)-3-(oct-1-yn-1-yl)-9*H*-fluoren-9-ylidene)-2,3-dihydro-1*H*-cyclopenta[a]naphthalen-5-yl)oxy)acetoxy)-3-(stearoyloxy)propyl (2-(trimethylammonio) ethyl) phosphate (MM1-PC)

Under light exclusion, an oven-dried crimp top vial equipped with a stirring bar was charged with **MM1** (21.3 mg, 40 µmol, 1.0 eq.) and **PC** (23.6 mg, 45 µmol, 1.1 eq.). The vial was crimped, flushed with nitrogen and CH_2Cl_2 (2 mL), 2,4,6-trichlorobenzoyl chloride (120 µL, 189 mg, 0.8 mmol, 19.0 eq.) and 1-methylimidazole (40 µL, 40.4 mg, 0.5 mmol, 13.0 eq.) were added and the reaction mixture stirred for 21 h at room temperature. The solvent was removed under reduced pressure, and the crude product purified *via* flash column chromatography (normal phase, gradient CH_2Cl_2 to CH_2Cl_2/MeOH (9:1) to CH_2Cl_2/MeOH/H_2O (5:4:1)) and

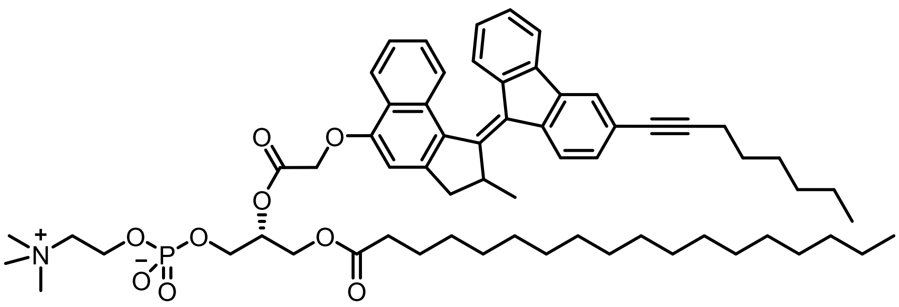

MM1-PC
$C_{63}H_{86}NO_9P$
Mw = 1032.35 g/mol

(CH_2Cl_2/MeOH/NH_{3aq} (4:1:0.1)) yielding **MM1-PC** as a yellow solid (*E*/*Z* mixture, 10.8 mg, 10 µmol, 26%).

¹H NMR (400 MHz, CD_3OD): δ = 8.44 (d, *J* = 8.5 Hz, 1H), 8.03 – 7.84 (m, 2H), 7.81 – 7.75 (m, 1H), 7.60 (t, *J* = 7.6 Hz, 1H), 7.54 – 7.47 (m, 1H), 7.45 – 7.32 (m, 2.5H), 7.20 (q, *J* = 7.1 Hz, 0.5H), 7.14 – 7.05 (m, 1H), 6.91 – 6.72 (m, 1H), 6.69 – 6.62 (m, 0.5H), 6.59 – 6.50 (m, 0.5H), 5.53 – 5.27 (m, 1H), 5.20 – 5.03 (m, 2H), 4.58 – 4.48 (m, 1H), 4.34 – 4.19 (m, 4H), 4.14 – 3.97 (m, 2H), 3.64 – 3.53 (m, 3H), 3.25 – 3.14 (m, 9H), 2.81 (d, *J* = 15.6 Hz, 1H), 2.51 – 2.32 (m, 2H), 2.26 – 2.14 (m, 2H), 1.70 – 1.43 (m, 6H), 1.38 (dd, *J* = 7.3, 3.1 Hz, 3H), 1.30 – 1.05 (m, 31H), 1.00 – 0.86 (m, 6H).
$^{13}C\{^1H\}$ NMR: Due to the amphiphilicity of the molecule no satisfactory carbon spectrum could be obtain displaying the aromatic protons beside the aliphatic ones.
^{31}P NMR (162 MHz, CD_3OD): δ = -0.13.
HRMS-ESI (ESI+): calculated for $C_{63}H_{86}NO_9PNa^+$ $[M+Na]^+$ 1054.5932, found 1054.5955.

(2*R*)-2-((5-((1-(9*H*-fluoren-9-ylidene)-2-methyl-2,3-dihydro-1*H*-cyclopenta[*a*]naphthalen-5-yl)oxy)pentanoyl)oxy)-3-(stearoyloxy)propyl (2-(trimethylammonio)ethyl) phosphate (MM2-PC)

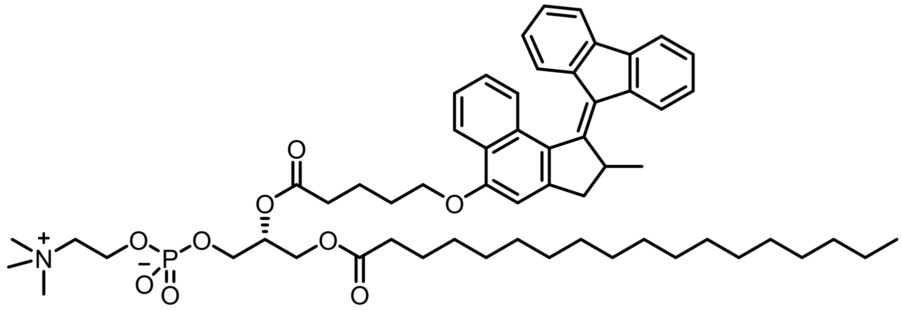

MM2-PC
$C_{58}H_{80}NO_9P$
Mw = 966.25 g/mol

Under light exclusion, an oven-dried crimp top vial equipped with a stirring bar was charged with **MM2** (6.2 mg, 13 µmol, 1.0 eq.) and **PC** (7.4 mg, 14 µmol, 1.0 eq.). The vial was crimped, flushed with nitrogen and CH_2Cl_2 (0.7 mL), 2,4,6-trichlorobenzoyl chloride (80 µL, 126 mg, 0.5 mmol, 12.5 eq.) and 1-methylimidazole (40 µL, 40.4 mg, 0.5 mmol, 13.0 eq.) were added and the reaction mixture stirred for 21 h at room temperature. The solvent was removed under reduced pressure, and the crude product purified *via* flash column chromatography (normal phase, gradient CH_2Cl_2 to CH_2Cl_2/MeOH (9:1) to CH_2Cl_2/MeOH/H_2O (5:4:1)) and (CH_2Cl_2/MeOH/NH_{3aq} (4:1:0.1)) yielding **MM2-PC** as a yellow solid (8.2 mg, 8.5 µmol, 63%).

^{1}H NMR (600 MHz, CD_3OD): δ = 8.36 (d, *J* = 8.5 Hz, 1H), 7.99 (d, *J* = 6.9 Hz, 1H), 7.89 – 7.84 (m, 1H), 7.78 (d, *J* = 7.5 Hz, 1H), 7.62 (d, *J* = 8.4 Hz, 1H), 7.47 – 7.43 (m, 1H), 7.39 – 7.34 (m, 2H), 7.33 – 7.29 (m, 1H), 7.17 (td, *J* = 7.4, 1.0 Hz, 1H), 7.11 (s, 1H), 6.75 (t, *J* = 7.7 Hz, 1H), 6.66 (d, *J* = 8.0 Hz, 1H), 5.35 – 5.26 (m, 1H), 4.48 (ddd, *J* = 12.1, 3.1, 1.2 Hz, 1H), 4.35 – 4.25 (m, 5H), 4.20 (dt, *J* = 12.0, 7.1 Hz, 1H), 4.07 – 4.01 (m, 2H), 3.64 – 3.61 (m, 2H), 3.55 (dd, *J* = 15.1, 5.6 Hz, 1H), 3.21 (s, 9H), 2.78 (d, *J* = 15.1 Hz, 1H), 2.61 – 2.51 (m, 2H), 2.29 – 2.23 (m, 2H), 2.10 – 1.95 (m, 4H), 1.55 – 1.47 (m, 2H), 1.38 (d, *J* = 6.7 Hz, 3H), 1.32 – 1.09 (m, 28H), 0.87 (t, *J* = 7.2 Hz, 3H).
^{13}C{^{1}H} NMR (101 MHz, CD_3OD): δ = 175.0, 174.7, 174.4, 158.8, 152.7, 150.8, 141.1, 141.1, 140.7, 138.5, 131.9, 129.5, 128.4, 128.1, 127.9, 127.5, 127.4, 126.8, 126.6, 126.1, 125.7, 124.8, 124.0, 120.6, 119.8, 106.4, 104.5, 88.8, 72.0, 69.3, 65.0, 63.7, 60.5, 55.5 – 53.5 (m), 46.3, 43.4, 36.6 – 34.3 (m), 33.1, 31.2 – 30.0 (m), 29.7, 26.0, 26.0, 23.7, 23.2, 23.1, 20.0, 14.5.
^{31}P NMR (162 MHz, CD_3OD): δ = -0.66.
HRMS-ESI (ESI+): calculated for $C_{58}H_{80}NO_9PNa^+$ $[M+Na]^+$ 988.5463, found 988.5473.

3. NMR Irradiation Studies

A solution (2.5 mM) of either **MM1-PC** or **MM2-PC** was prepared in methanol-d_4 and transferred into a NMR tube which subsequently fitted with a glass optic fiber for *in situ* irradiation studies. The sample was place in a Varian Unity Plus 500 MHz NMR and cooled to -15 °C. ^{1}H NMR spectra were recorded before irradiation, while irradiating with 405 nm until reaching PSS, and during the THI step until completed.

Note: In the case of symmetrically lower-half molecular motors, it is important to distinguish between two possible processes. A photochemical back-reaction from the metastable isomer to the stable isomer, which does not lead to unidirectional rotation of the motor but rather a back and forth type switching, and the thermal helix inversion leading to unidirectional rotation. In our case, a photochemical back-reaction is very unlikely as the UV-vis spectra of stable and metastable isomers don't show a significant band separation and significantly higher molar absorption coefficient. To achieve unidirectional rotation the initial photochemical step has to be followed by a thermal step (thermal helix inversion), which for the present motor leads to a compound indistinguishable from the original stable isomer. The following photochemical step which initiates the second 180° rotation can therefore be performed using light of the same wavelength as for the first step. This explanation is applicable for **MM2-PC** as it bears a symmetrically lower-half. However, a distinct situation was envisioned for **MM1-PC** as the bottom-half alkyl chain desymmetrizes the lower half of the molecule. Due to the high amount of proton peaks of the mentioned compound it was not possible to determine the four distinct forms (*E/Z* of both metastable and stable isomers) and the compound will be referred with stable and metastable forms.

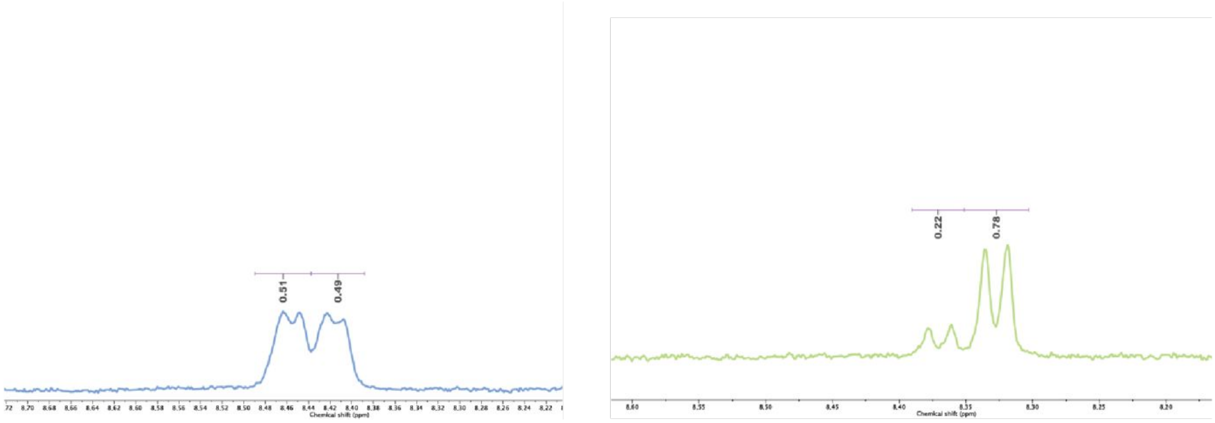

Figure S1: Partial ^{1}H-NMR spectra showing PSS ratio of stable and metastable isomers of **MM1-PC** (left, blue) and **MM2-PC** (green, right) in methanol-d_4 at – 15 °C.

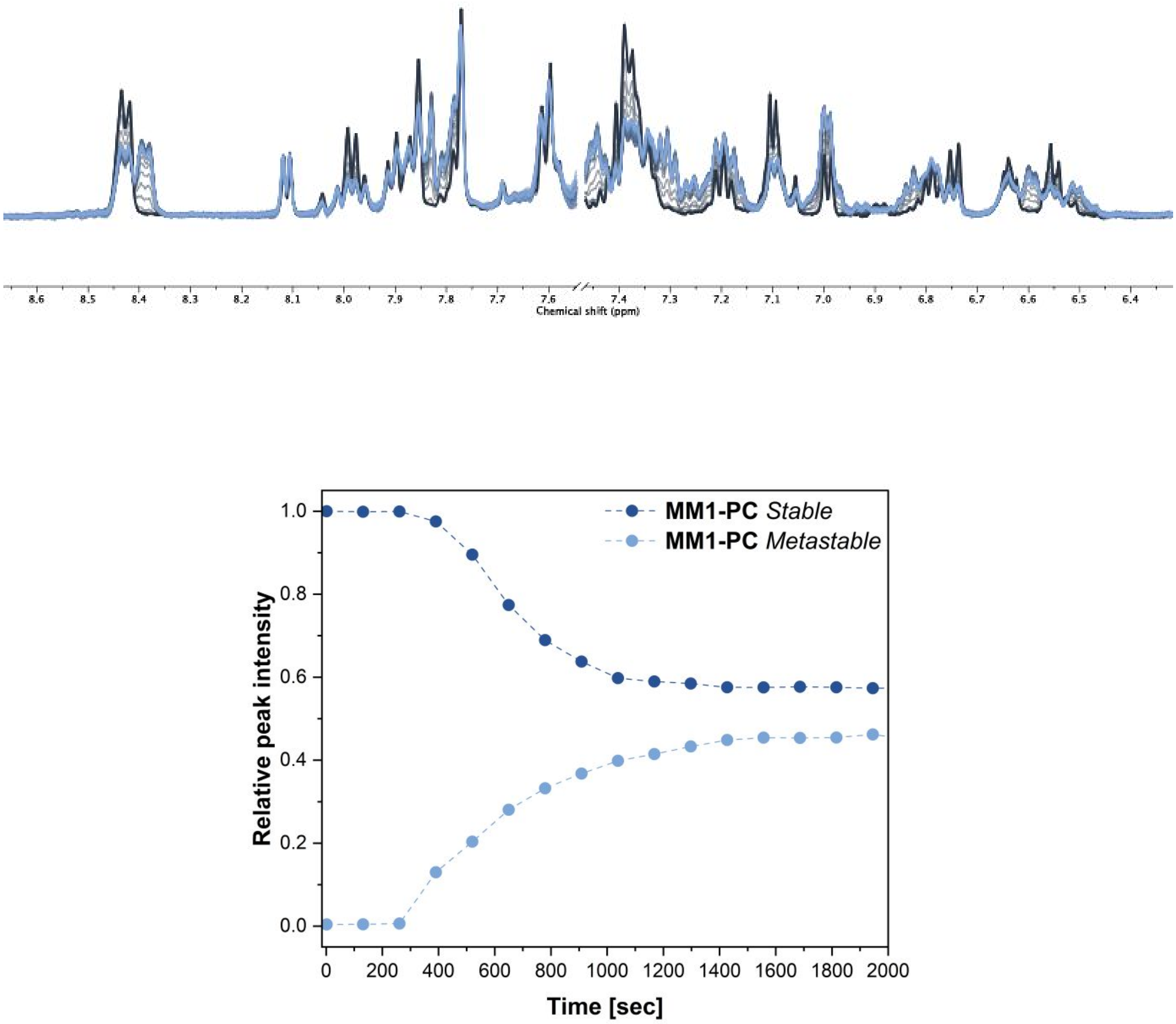

Figure S2: ^{1}H NMR irradiation studies of **MM1-PC** in methanol-d_4 (c = 2.5 mM, 15 °C), showing the spectra changes under 405 nm *in situ* irradiation of the stable isomer until reaching PSS (Top, from black to colour). Kinetics of the different formed species of **MM1-PC** (Bottom). Irradiation started at 280 sec.

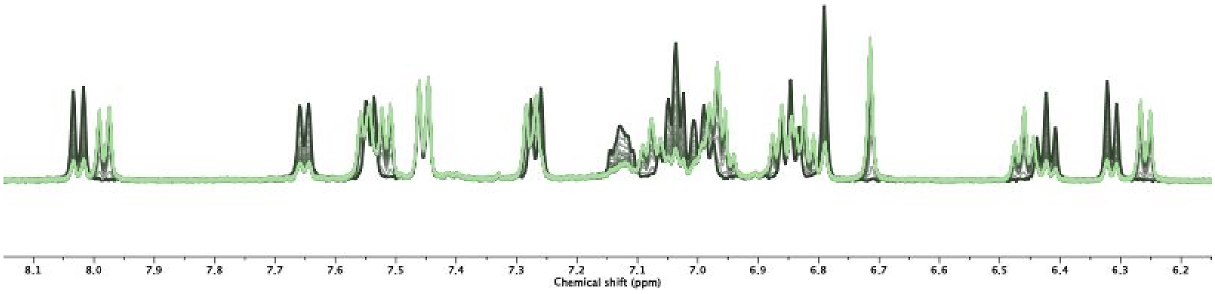

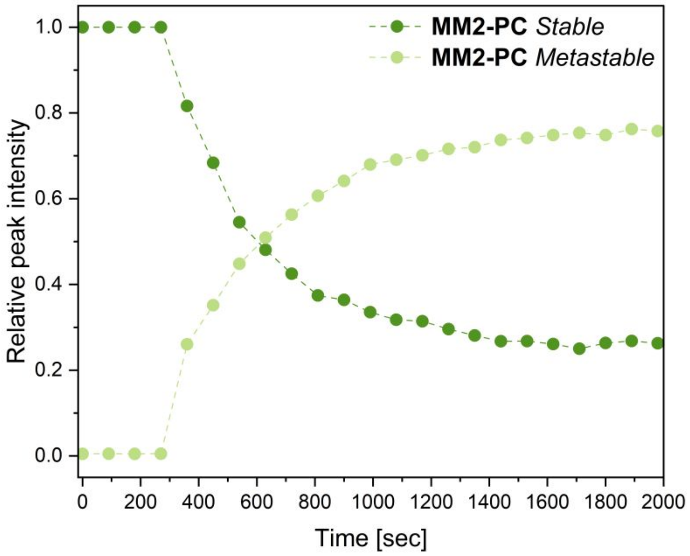

Figure S3: ^{1}H NMR irradiation studies of **MM2-PC** in methanol-d_4 (c = 2.5 mM, 15 °C), showing the spectra changes under 405 nm *in situ* irradiation of the stable isomer until reaching PSS (Top, from black to colour). Kinetics of the different formed species of **MM2-PC** (Bottom). Irradiation started at 280 sec.

4. UV-Vis Spectroscopy

UV–Vis spectroscopy was used for determination of molecular motor photoisomerization, quantum yield determination and thermodynamic studies. Briefly, samples containing free-standing lipid systems or molecular motor solutions were measured using an Agilent 8453 UV–Vis Diode Array System, equipped with a Quantum Northwest Peltier controller. If specified, irradiations were done using a built-in setup coupled to an LED. Solutions were prepared and measured using a quartz cuvette with 1 cm optical path.

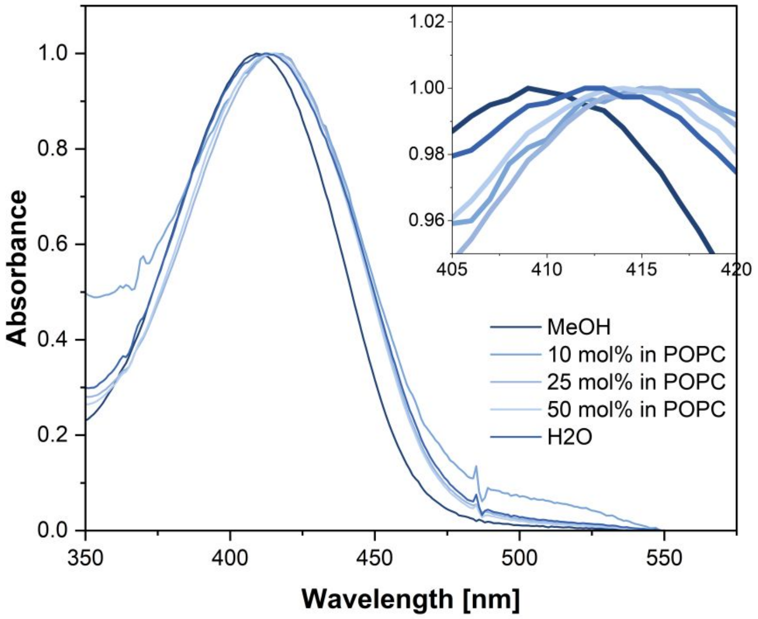

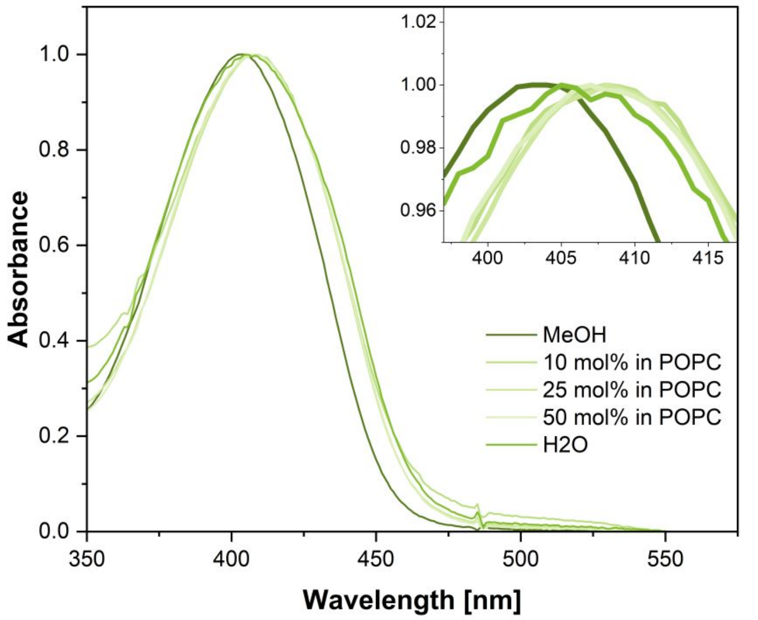

Figure S4: Maxima peak absorbance of **MM1-PC** (top) and **MM2-PC** (bottom) in MeOH or self-assembled in pure H_2O, or in conjugation with POPC at different ratios in H_2O. Insert showing zoomed area of the peak position.

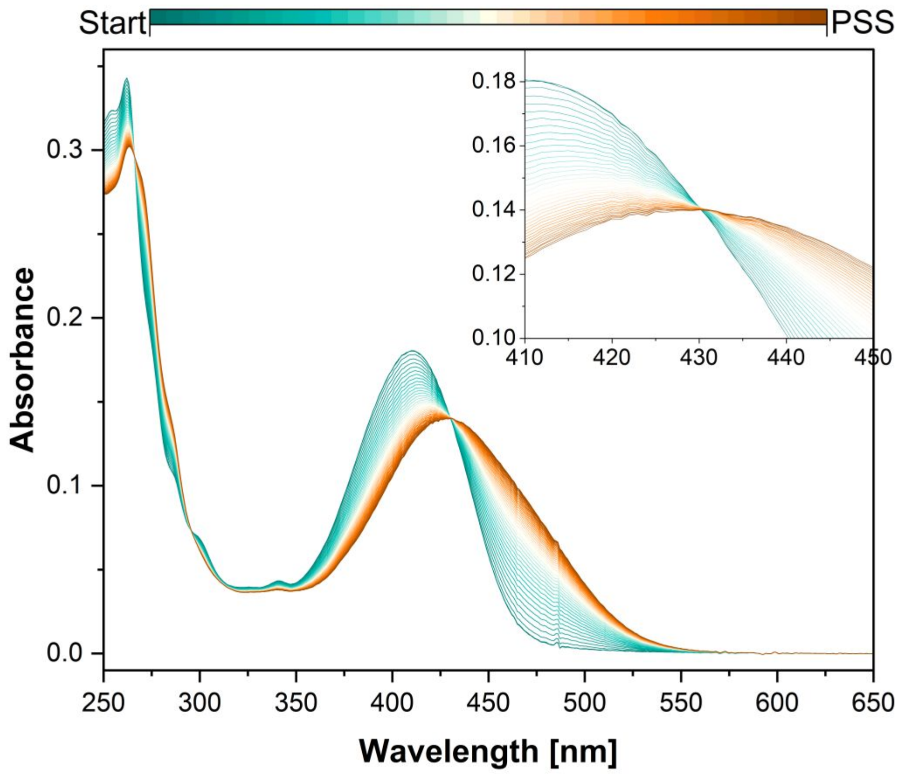

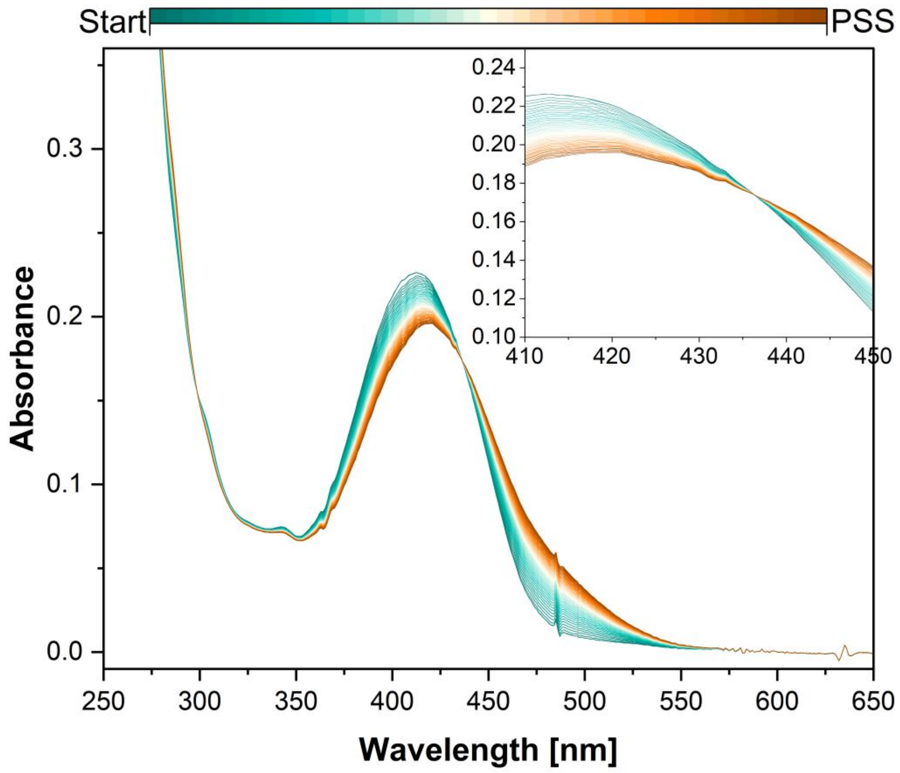

Figure S5: Evolution of **MM1-PC** absorption spectra under 405 nm LED irradiation from the stable state until reaching PSS in MeOH (top) and H_2O (bottom). Insert shows the isosbestic point.

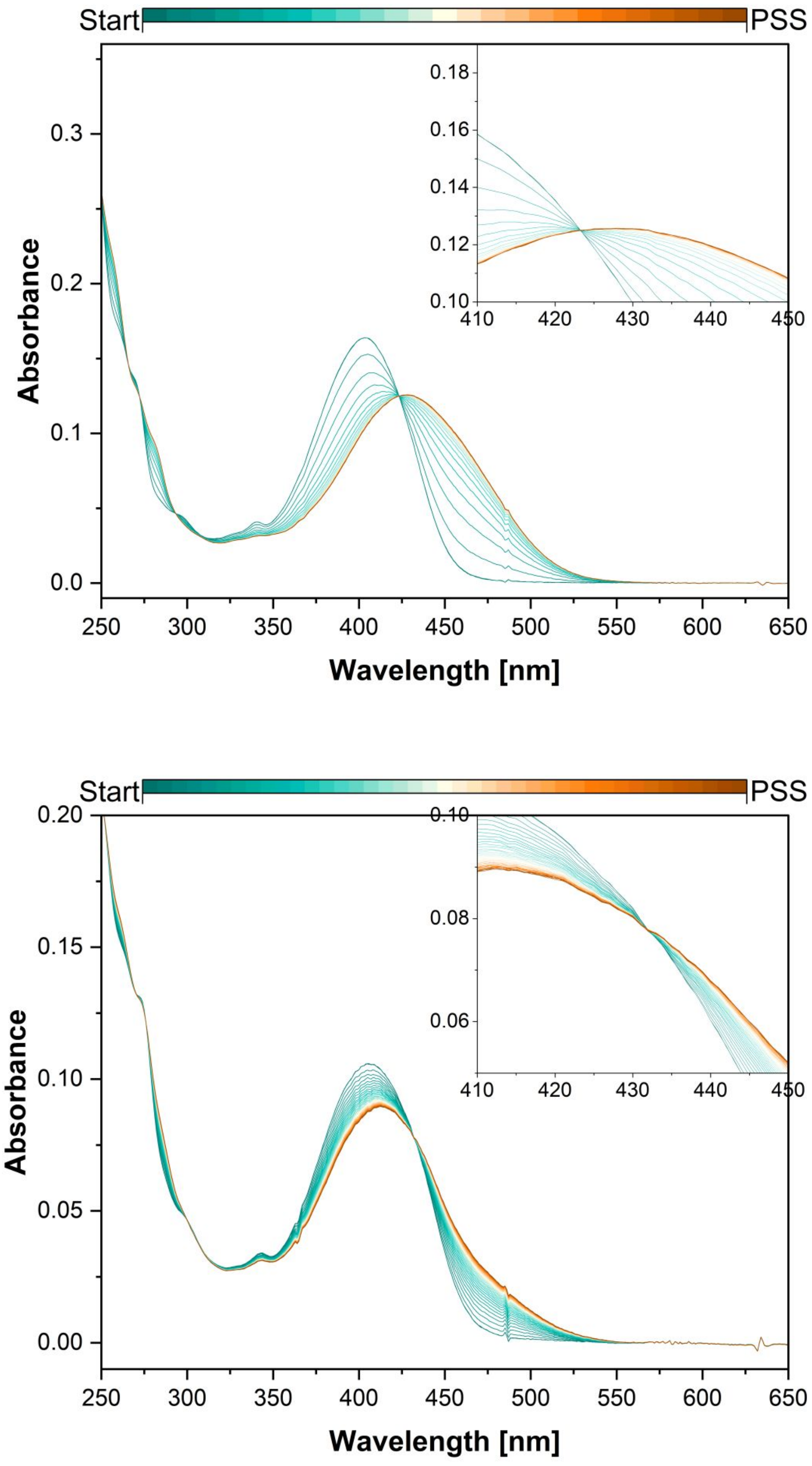

Figure S6: Evolution of **MM2-PC** absorption spectra under 405 nm LED irradiation from the stable state until reaching PSS in MeOH (top) and H_2O (bottom). Insert shows the isosbestic point.

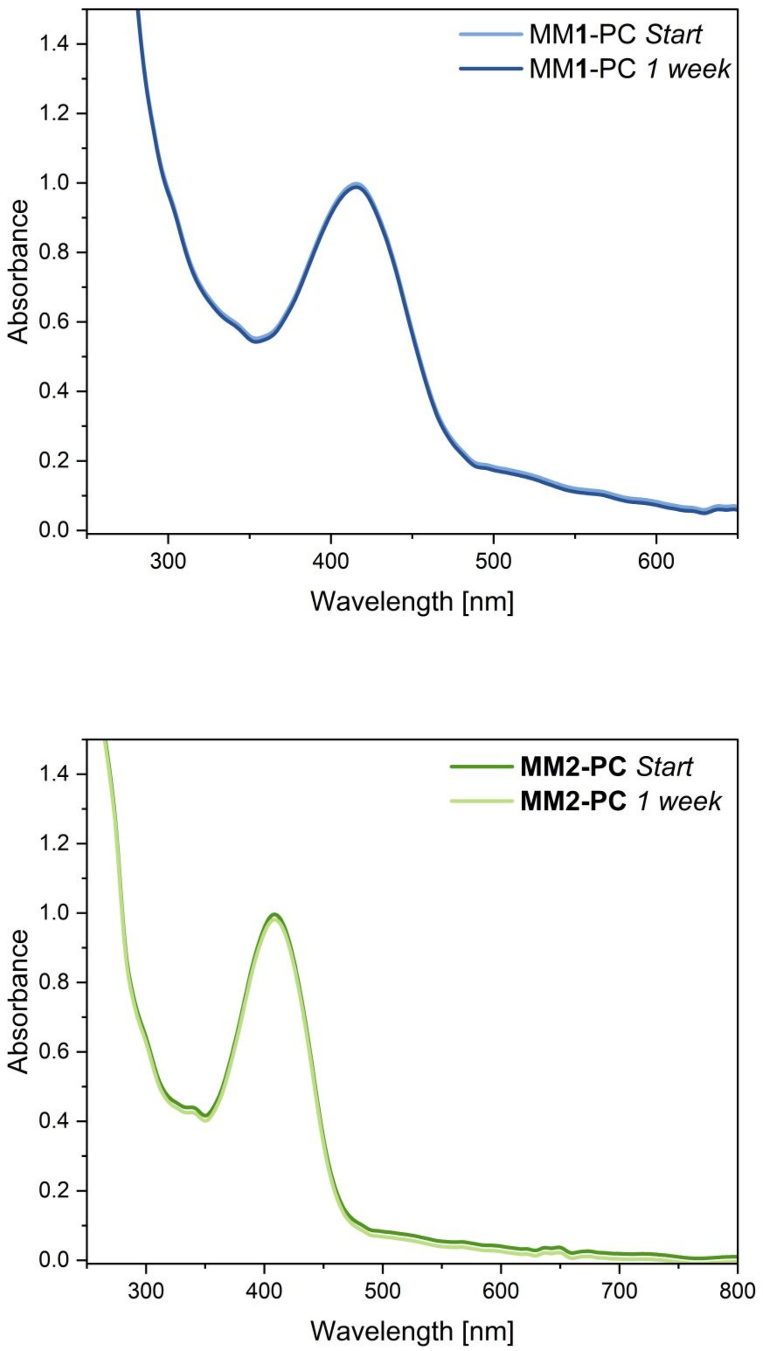

Figure S7: MM1-PC (top) and **MM2-PC** (bottom) self-assembled in H_2O systems stability over 1 week in solution in the dark.

a. Quantum Yield Determination

Quantum yield determinations of **MM1-PC** and **MM2-PC** in solution and in combination with lipid systems were carried out at 5 °C, ensuring the stability of the metastable state over the duration of the study. For all the experiments, solutions of the stable state in the different environments at a concentration of 50 μM were prepared in a 10 mm quartz cuvette. UV-Vis measurements were taken every 1 s over the first 60 s of constant irradiation until PSS was reached at 405 nm, following the evolution of the absorption maximum. Baseline corrections were carried out to account for baseline drifting, and the absorbance at 405 nm over the course of the measurement was extracted. The data was subsequently processed in Copasi, using a method outlined by Stranius & Börjesson, based on Equation 1[5]:

$$\frac{d[A]}{dt} = -\frac{QY_{SM} * I * \beta_S(t)}{N_A * V} + \frac{QY_{MS} * I * \beta_M(t)}{N_A * V}$$

Where I is the molar photon flux of the 405 nm LED (1.50E^{-04} mE/s, previously determined by chemical actinometry[6], V is the total volume of the irradiated solution (1.5 mL), and β is the fraction of photons absorbed by either the stable or metastable isomer, a number that is determined using the PSS ratio and the corresponding molar extinction coefficient of each isomer. Molar absorption coefficients of stable (ε_S $[M^{-1}c^{-1}]$) and metastable (ε_M $[M^{-1}c^{-1}]$) motor isomers were determined by measuring UV-Vis spectra at three different known concentrations and volumes following Lambert-Beer law[7]. Data shown in Table S1 corresponds to the averaged QY data over five separate runs for each system.

Table S1: Photoisomerization quantum yield values for 405 nm irradiation of **MM1-PC** and **MM2-PC** in different environments.

Motor	Environment	QY_{SM}	QY_{MS}
**MM1-PC**	MeOH	3.17 ± 1.54%	19.72 ± 1.54%
	10 mol% MM in POPC (SUVs in H_2O)	1.11 ± 0.18%	4.72 ± 0.18%
	25 mol% MM in POPC (SUVs in H_2O)	0.35 ± 0.05%	1.57 ± 0.05%
	50 mol% MM in POPC (SUVs in H_2O)	0.3 ± 0.08%	0.98 ± 0.08%
	H_2O	0.4 ± 0.17%	0.17 ± 0.01%
**MM2-PC**	MeOH	5.81 ± 0.28%	2.82 ± 0.28%
	10 mol% MM in POPC (SUVs in H_2O)	2.18 ± 0.56%	1.39 ± 0.56%
	25 mol% MM in POPC (SUVs in H_2O)	0.68 ± 0.11%	0.11 ± 0.01%
	50 mol% MM in POPC (SUVs in H_2O)	0.54 ± 0.13%	0.13 ± 0.01
	H_2O	3.57 ± 0.77%	1.03 ± 0.77%

b. Eyring Analysis

Solutions of the stable state in the different environments at a concentration of 50 µM were prepared in a 10 mm quartz cuvette and irradiated with 405 nm LED until PSS. Eyring plot analysis of the thermal isomerization processes in different environments was performed by monitoring the decrease in absorption of a UV-Vis sample over time in three different temperatures. Rate constants (k) as changes of absorbance over time were determined by fitting a first-order decay and plotted to determine the thermodynamic parameters of the thermal helix inversions using Origin 2023 software and a least squares analysis was performed on the Eyring equation to retrieve the $\Delta G^{‡}$. The activation parameters at 25 °C for the metastable → stable process could be determined and are displayed in Table S2. Eyring plots and half-lives times at 25 °C for the studied environments are shown in Figure S8 and S9 and fitting of Eyring analysis in Table S3.

Table S2: Activation parameters of **MM1-PC** and **MM2-PC** in different environments at 25 °C.

Motor	Environment	$\Delta G^{‡}[kcal\ mol^{-1}]$	$\Delta H^{‡}$ $[kcal\ mol^{-1}]$	$\Delta S^{‡}$ $[cal\ K^{-1} mol^{-1}]$	$t_{1/2}\ [sec]$
MM1-PC	MeOH	20.787 ± 0.10	14.00	-22.75	192
	10 mol% in POPC	21.139 ± 0.03	14.99	-20.63	349
	25 mol% in POPC	21.308 ± 0.06	17.24	-13.63	464
	50 mol% in POPC	21.315 ± 0.03	14.73	-22.10	470
	H_2O	21.385 ± 0.06	16.14	-17.59	528
MM2-PC	MeOH	20.685 ± 0.08	14.56	-20.55	162
	10 mol% in POPC	20.719 ± 0.02	14.25	-21.71	172
	25 mol% in POPC	20.853 ± 0.02	15.93	-16.51	215
	50 mol% in POPC	20.890 ± 0.08	14.83	-20.33	229
	H_2O	20.071 ± 0.01	21.18	3.70	58

Table S3: Linear fitting data of the Eyring plot analysis of **MM1-PC** and **MM2-PC**.

	Environment	Intercept	Slope	R-Square
MM1-PC	MeOH	-10.53 ± 3.63	-7312 ± 1066	0.979
	10 mol% in popc	-10.12 ± 1.01	-7616 ± 296	0.998
	25 mol% in popc	-6.37 ± 1.93	-8817 ± 565	0.996
	50 mol% in popc	-11.38 ± 1.02	-7336 ± 298	0.998
	H_2O	-8.32 ± 2.10	-8275 ± 616	0.994

	Environment	Intercept	Slope	R-Square
MM2-PC	MeOH	-10.36 ± 2.75	-7322 ± 792	0.988
	10 mol% in popc	-11.11 ± 0.75	-7114 ± 220	0.999
	25 mol% in popc	-8.10 ± 0.83	-8077 ± 243	0.999
	50 mol% in popc	-9.49 ± 2.96	-7678 ± 867	0.987
	H_2O	1.97 ± 0.42	-10687 ± 124	0.999

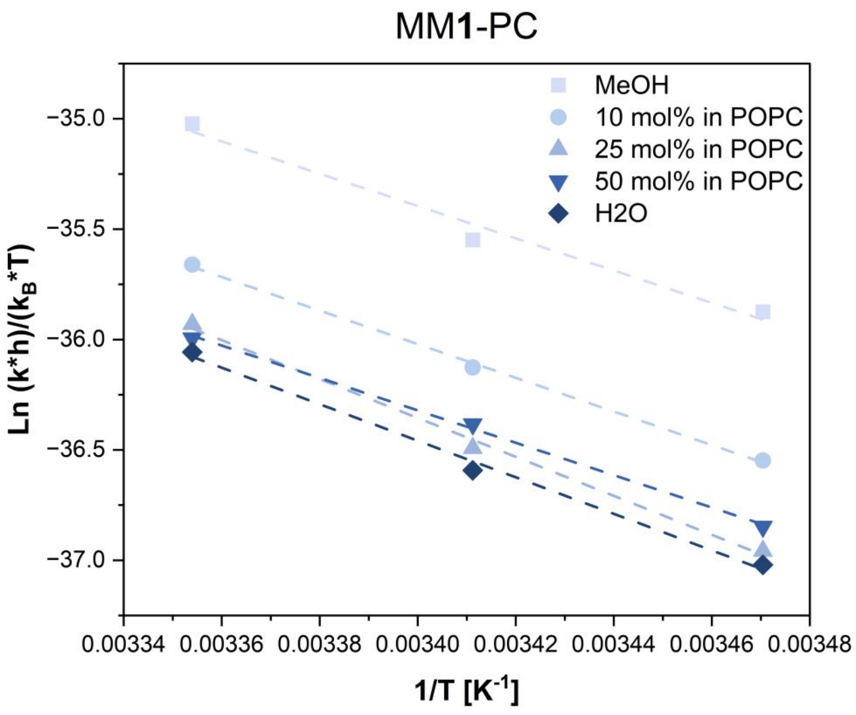

Figure S8: Eyring plot analysis of **MM1-PC** monitoring the decrease in absorption at 475 nm at three different temperatures.

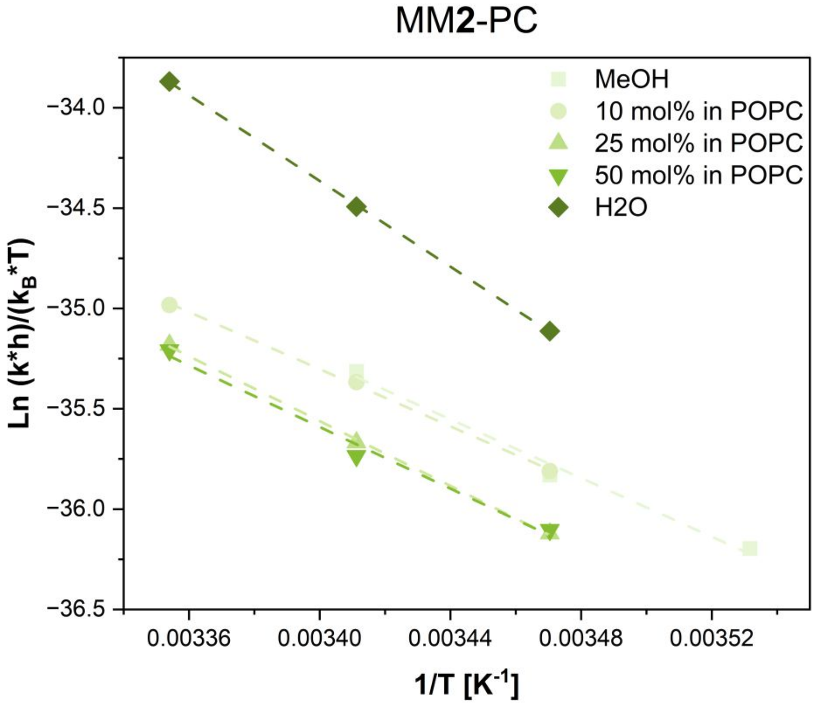

Figure S9: Eyring plot analysis of **MM2-PC** monitoring the decrease in absorption at 475 nm at three different temperatures.

5. Fluorescence Lifetime Spectroscopy

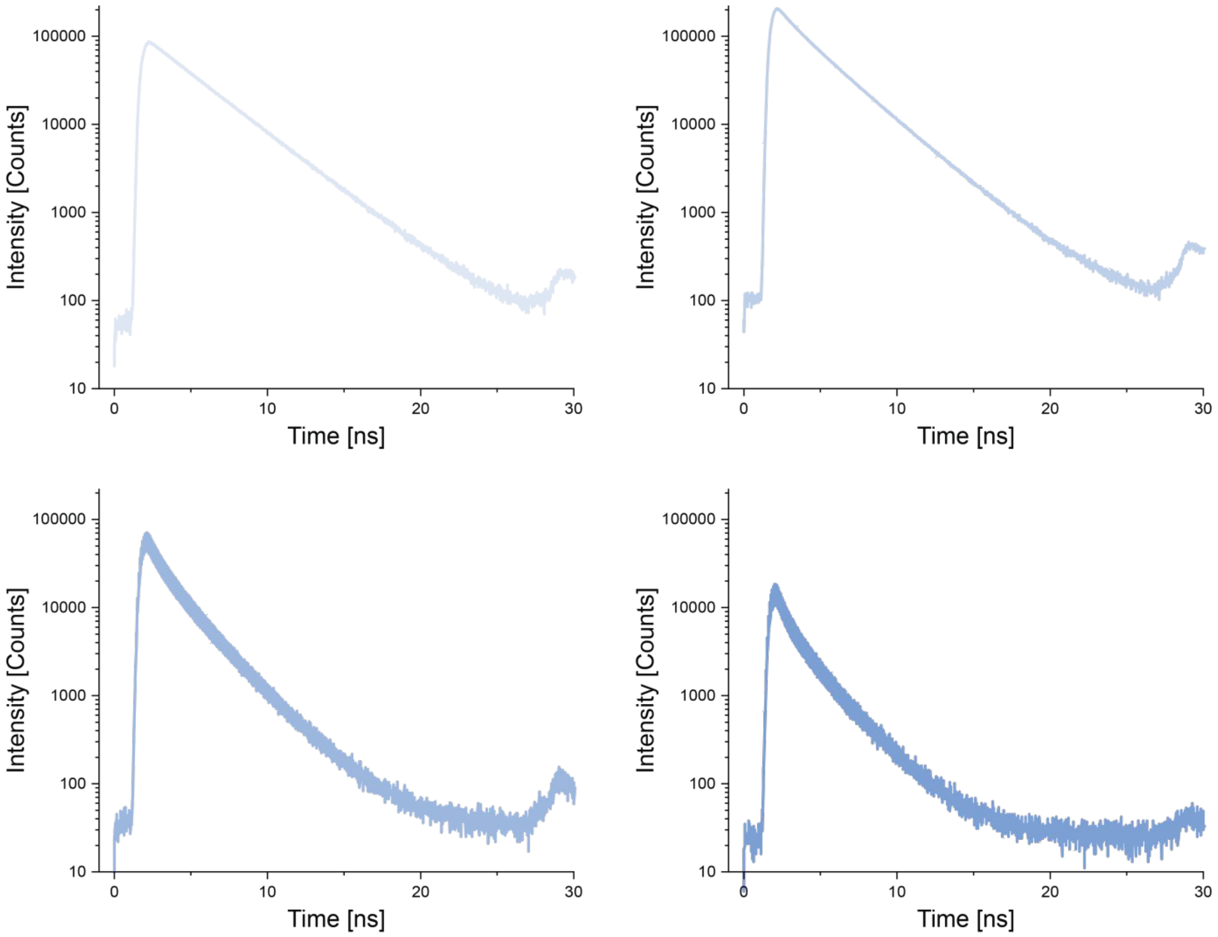

Figure S10: Decrease in the fluorescence lifetime of the A655-PE lipid conjugate dye upon increasing percentage (top-left to right-bottom, 1, 5, 10, 25 mol%) of **MM1-PC** incorporation in the membranes.

MicroTime200 (PicoQuant GmBH, Germany) equipped with a dual SPAD detection unit and a MultiHarp 150 TCSPC unit was used to measure fluorescence lifetime of A655-DOPE present in the membrane. SUVs were prepared with the desired concentration of the **MM1-PC** and 0.002 mol% A655-DOPE. The SUVs were deposited on a #1.5 coverslip and excited with a 641 nm laser line and the emission was collected through a 50 μm pinhole. Single photon counting histograms were collected for 180 seconds for each measurement. SymphoTime64 was used to analyze and fit the decay curves an get the fluorescence lifetime for each case.

6. Cryo- Electron Microscopy

3 μL of SUVs prepared at 2.5 mg/mL was applied on a glow-discharged Quantifoil 1.2/1.3 holey carbon grid, Cu 300 mesh (Quantifoil Micro Tools GmbH) and blotted for 3.5 sec in 100% humidity at 20 °C and immediately plunge-frozen into an ethane-propane 1:1 mixture using the Leica EM GP2 (Leica). Grids were then loaded into Titan Krios G4 transmission electron microscope operated at 300 kV, equipped with Falcon4i direct detector camera, Selectris X energy filter and CFEG electron source (ThermoFisher Scientific). Images were collected automatically using EPU software (ThermoFisher Scientific) in electron-event representation (EER) format [8]. Micrographs were collected at a magnification of 135,000x, resulting in a pixel size of 0.95 Å, with an acquisition time of 2.9 seconds and with a total dose of 40 e^-/Å^2. EER movies were rendered as an 8k x 8k grid and further Fourier cropped into a 4k x 4k grid using RELION-5.0 [9]. The movies were motion-corrected, dose-weighted, and averaged using RELION-5.0 MotionCorr2 algorithm[10] . Frames were dose-fractionated into groups resulting in a dose of 0.9 e-/Å2 per fraction. CTF estimation was performed by CTFfind4 algorithm [11]. crYOLO software was used to automatically recognize and pick edges of the lipid vesicles (v1.9.2). A new picking model was trained from scratch by manually picking the edges of the lipid vesicles in 30 micrographs. Particles were then imported to cryoSPARC 4.0 software and extracted with a box size of 256×256 pixels [12]. Reference free 2D classification was then performed on the extracted particles.

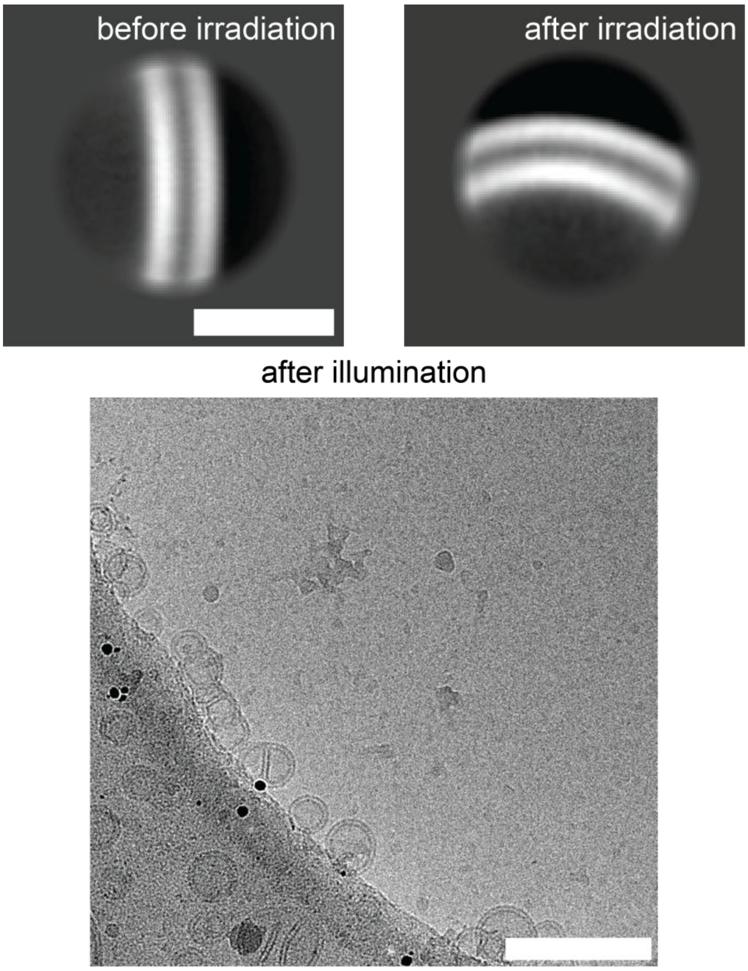

Figure S11: Classified Cryo-TEM images of the lipid bilayer composing of POPC:**MM1-PC** (1:1). Membrane reconstruction before and after irradiation (top). Zoom out of vesicles after irradiation (bottom). Irradiation @405 nm, 10 min. Scale bar 10 nm (top) and 100 nm (bottom).

7. Critical Aggregation Concentration

The critical aggregation concentration of **MM1-PC** and **MM2-PC** in H_2O using the Nile Red Fluorescence Assay[13,14]. A stock solution of Nile Red (1 mM, MeOH) was diluted with **MM1-PC** and **MM2-PC** solutions in H_2O to a final concentration of 2 µM and incubated for 1h. Concentrations of **MM1-PC** and **MM2-PC** before and after 1 min irradiation with a 405 nm LED were ranging from 0.1 µM to 100 µM. Sample solutions were excited at 550 nm and emission was recorded from 590 to 750 nm using a JASCO FP6200 spectrometer. Fluorescent intensity was calculated by measuring the increase compared to a reference solution of Nile Red in H_2O. The increase in fluorescent intensity was plotted against their respective **MM1-PC** and **MM2-PC** concentrations and the intersection between the linear fittings determined the critical aggregation concentrations.

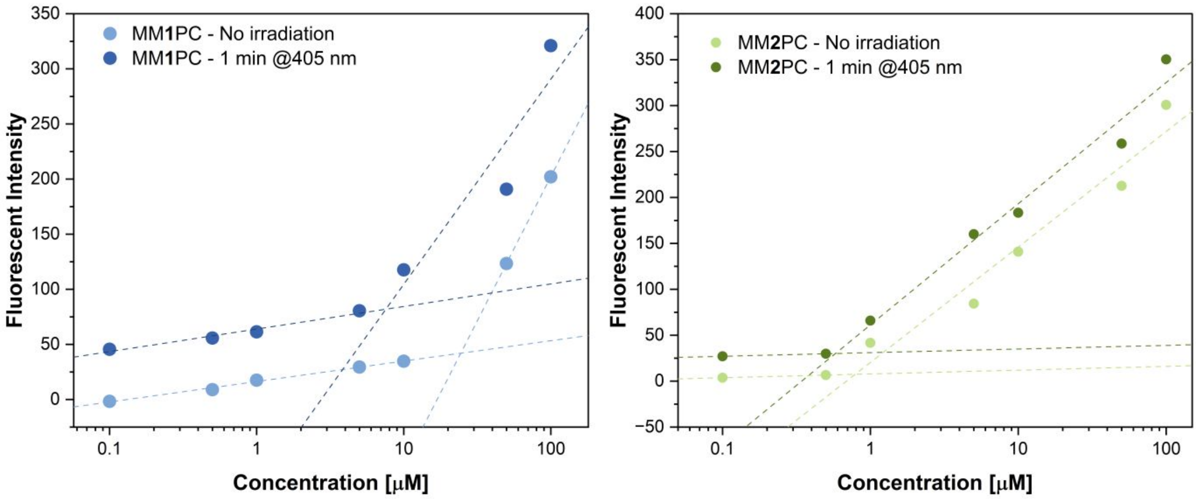

Figure S12: Increase in Nile Red (2 µM) fluorescence intensity in **MM1-PC** (blue, left) and **MM2-PC** (green, right) increasing concentrations of non-irradiated (clear) and irradiated (dark) solutions in H_2O.

Table S4: CAC values obtained for **MM1-PC** and **MM2-PC**.

**MM1-PC**	Before irradiation	**24.417 µM**
	1 min @405 nm	**7.565 µM**
**MM2-PC**	Before irradiation	**0.788 µM**
	1 min @405 nm	**0.580 µM**

8. Giant Unilamelar Vesicles

Giant Unilamellar Vesicles (GUVs) were prepared through electroformation with Pt electrodes. Briefly, 6 μL of the desired lipid mixture in chloroform was deposited on two platinum electrodes in custom chambers. The electrodes were dried under an Argon flow and then under a vacuum for 30 minutes. The electrodes were then inserted in a 300 mM sucrose solution and connected to a signal generator at $2V_{rms}$ at 10 Hz for 1.5 hours and then lowered to 2 Hz for 30 minutes. The GUVs were collected in an Eppendorf with equal volume of equimolar glucose solution.

a. GUVs imaging – Spinning Disk Confocal Microsocpy

Spinning disk confocal imaging was performed on a Nikon/Yokogawa CSU-W1 spinning disk confocal microscope, using 405, 488, and 641 nm laser lines. The 50 μm pinhole spinning disk was used at 4000 rpm. The sample was illuminated through a Nikon Apo TIRF 60x Oil DIC N2 immersion oil objective and the images were acquired in pco.edge sCMOS cameras (pco.edge 4.2 LT USB) at 100 ms exposure time. For z-stack imaging, the desired optical sectioning was set at 0.2 μm.

References

[1] P. Urban, S. D. Pritzl, D. B. Konrad, J. A. Frank, C. Pernpeintner, C. R. Roeske, D. Trauner, T. Lohmüller, *Langmuir* **2018**, *34*, 13368–13374.

[2] L. Pfeifer, M. Scherübl, M. Fellert, W. Danowski, J. Cheng, J. Pol, B. L. Feringa, *Chem. Sci.* **2019**, *10*, 8768–8773.

[3] L. Pfeifer, N. V Hoang, M. Scherübl, M. S. Pshenichnikov, B. L. Feringa, *Sci. Adv.* **2020**, *6*, eabb6165.

[4] L. Pfeifer, N. V Hoang, S. Crespi, M. S. Pshenichnikov, B. L. Feringa, *Sci. Adv.* **2022**, *8*, eadd0410.

[5] K. Stranius, K. Börjesson, *Scientific Reports 2017 7:1* **2017**, *7*, 1–9.

[6] M. Montalti, A. Credi, L. Prodi, M. T. Gandolfi, *Handbook of Photochemistry* **2006**, DOI 10.1201/9781420015195.

[7] D. F. Swinehart, *J Chem Educ* **1962**, *39*, 333–335.

[8] H. Guo, E. Franken, Y. Deng, S. Benlekbir, G. Singla Lezcano, B. Janssen, L. Yu, Z. A. Ripstein, Y. Z. Tan, J. L. Rubinstein, *IUCrJ* **2020**, *7*, 860–869.

[9] D. Kimanius, L. Dong, G. Sharov, T. Nakane, S. H. W. Scheres, *Biochemical Journal* **2021**, *478*, 4169–4185.

[10] S. Q. Zheng, E. Palovcak, J.-P. Armache, K. A. Verba, Y. Cheng, D. A. Agard, *Nat Methods* **2017**, *14*, 331–332.

[11] A. Rohou, N. Grigorieff, *J Struct Biol* **2015**, *192*, 216–221.

[12] A. Punjani, J. L. Rubinstein, D. J. Fleet, M. A. Brubaker, *Nat Methods* **2017**, *14*, 290–296.

[13] F. K. C. Leung, T. Van Den Enk, T. Kajitani, J. Chen, M. C. A. Stuart, J. Kuipers, T. Fukushima, B. L. Feringa, *J Am Chem Soc* **2018**, *140*, 17724–17733.

[14] F. Tantakitti, J. Boekhoven, X. Wang, R. V. Kazantsev, T. Yu, J. Li, E. Zhuang, R. Zandi, J. H. Ortony, C. J. Newcomb, L. C. Palmer, G. S. Shekhawat, M. O. De La Cruz, G. C. Schatz, S. I. Stupp, *Nature Materials 2016 15:4* **2016**, *15*, 469–476.

Appendix

NMR Spectra

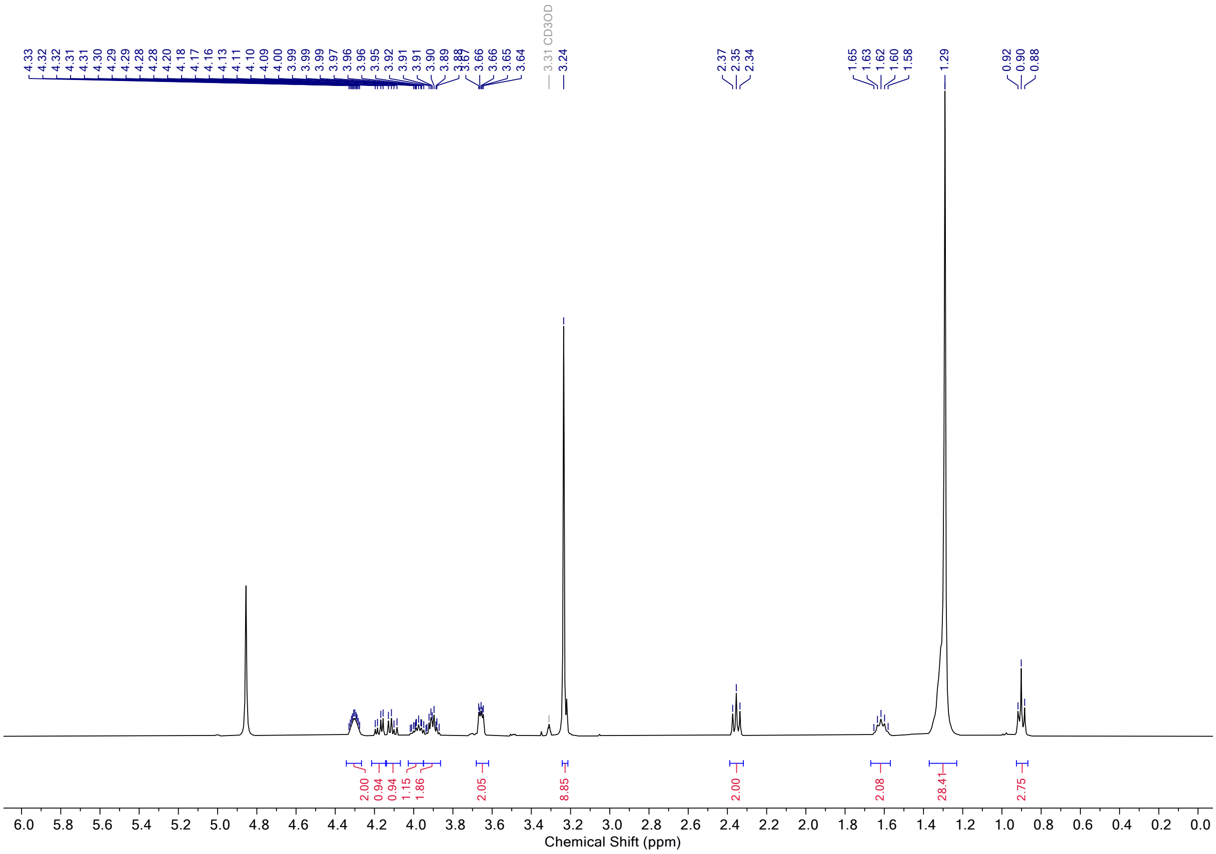

Figure S13: ^{1}**H NMR** spectrum of **PC** (CD_3OD).

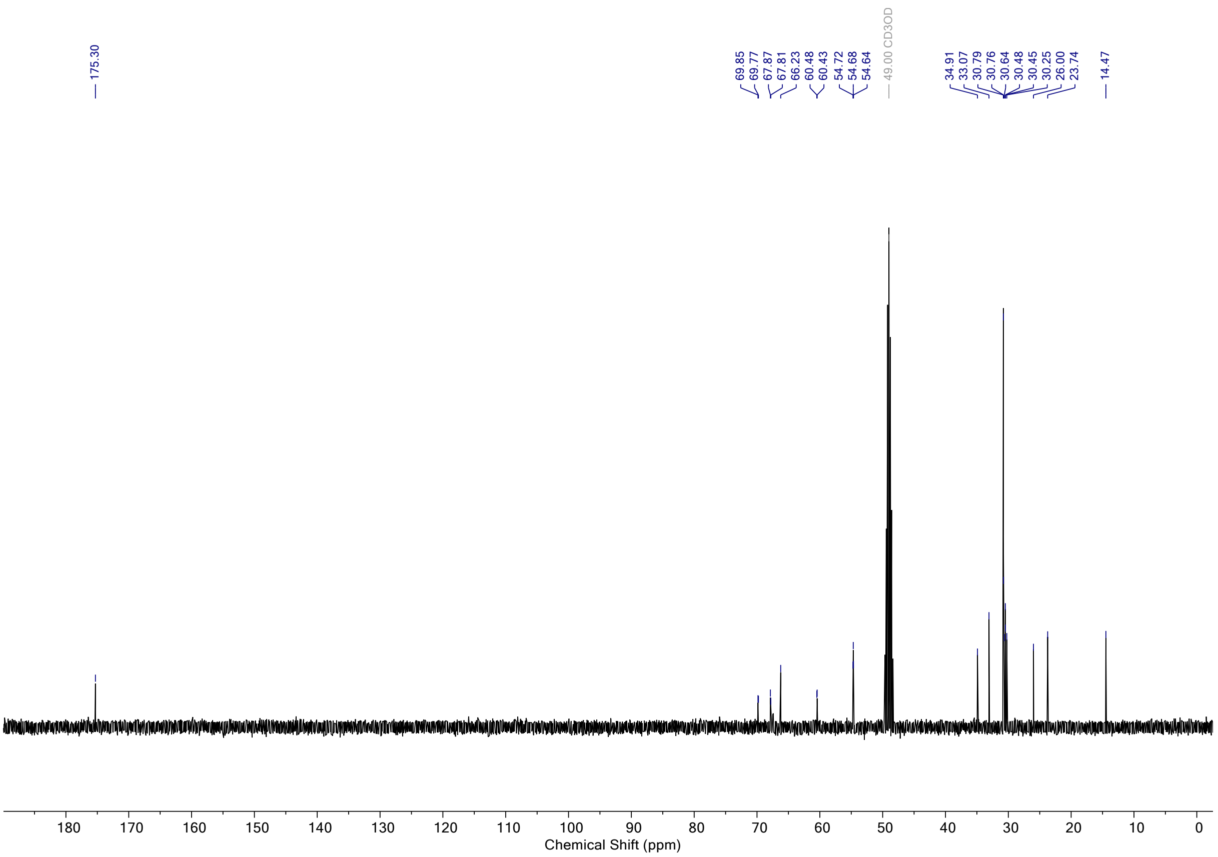

Figure S14: **$^{13}C\{^1H\}$ NMR** spectrum of **PC** (CD_3OD).

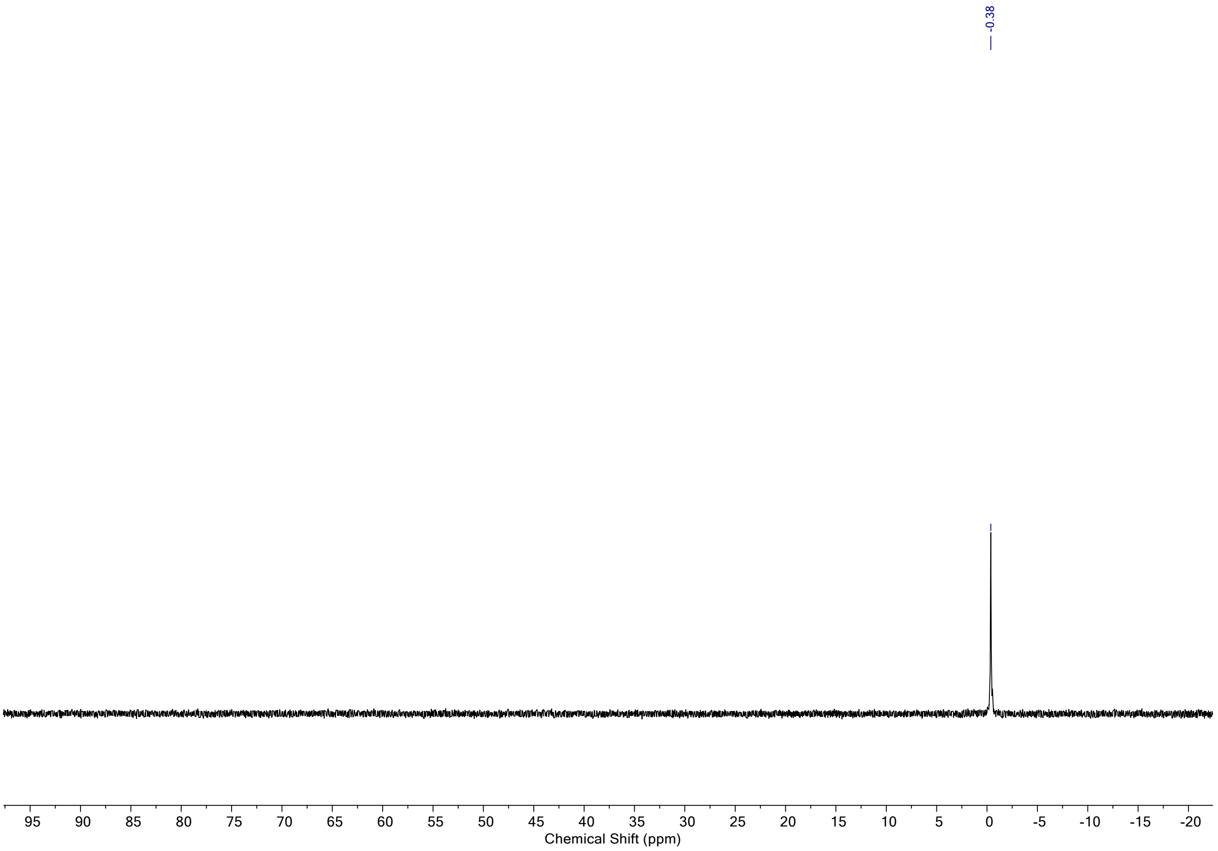

Figure S15: **^{31}P NMR** spectrum of **PC** (CD_3OD).

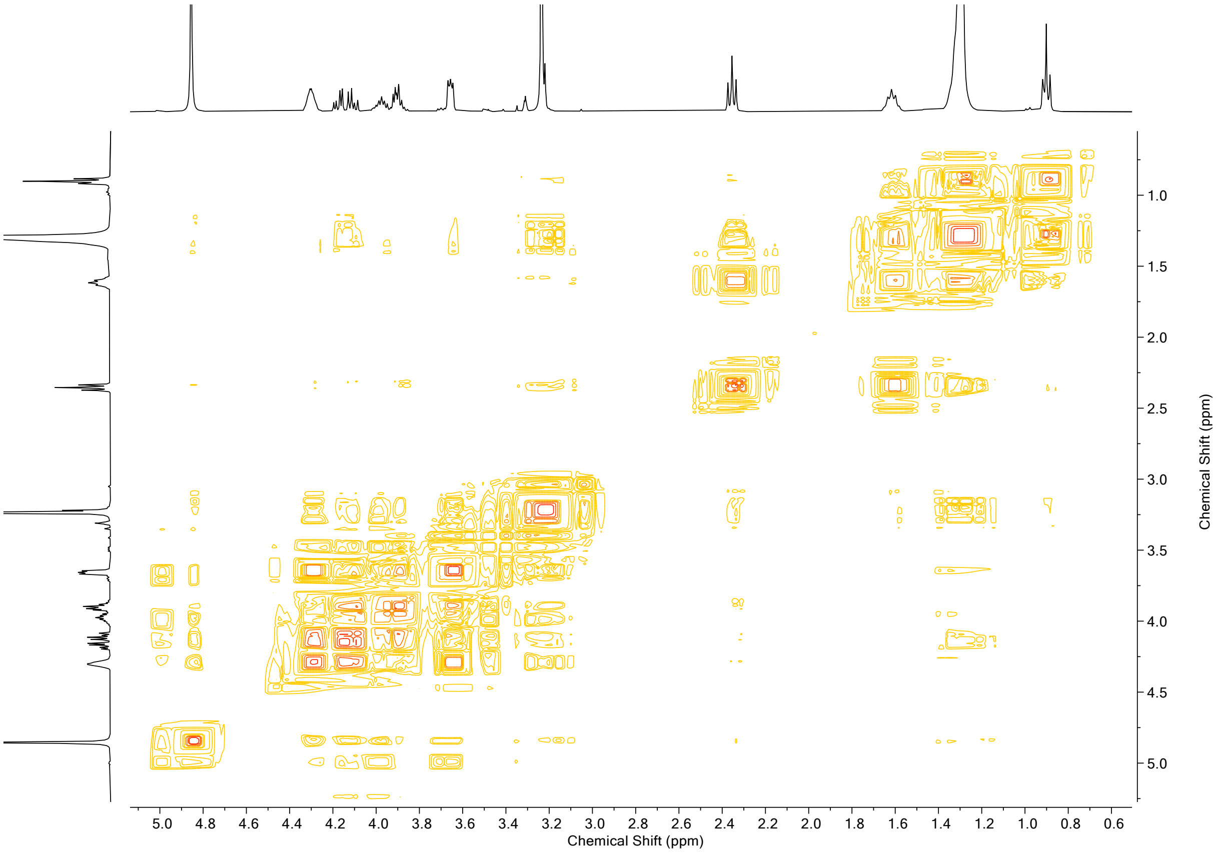

Figure S16: COSY spectrum of **PC** (CD_3OD).

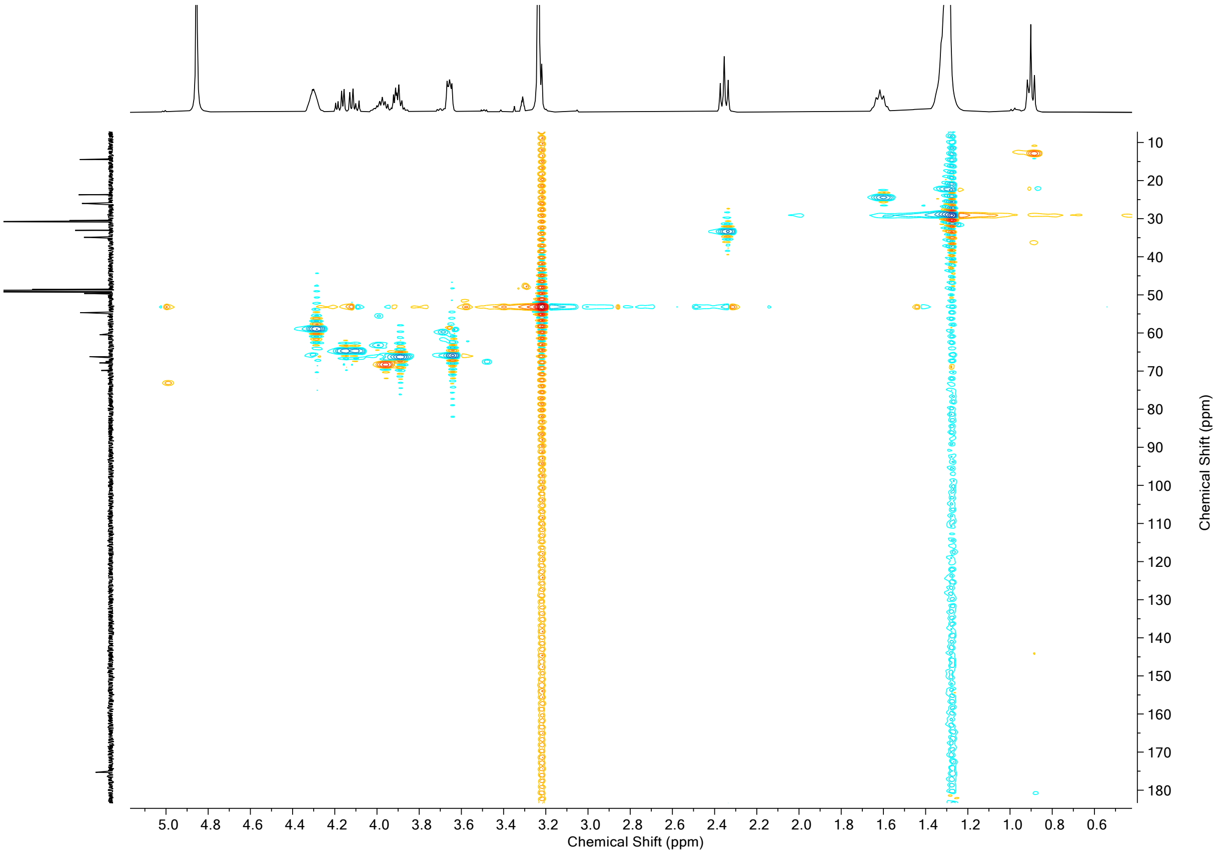

Figure S17: HSQC spectrum of **PC** (CD_3OD).

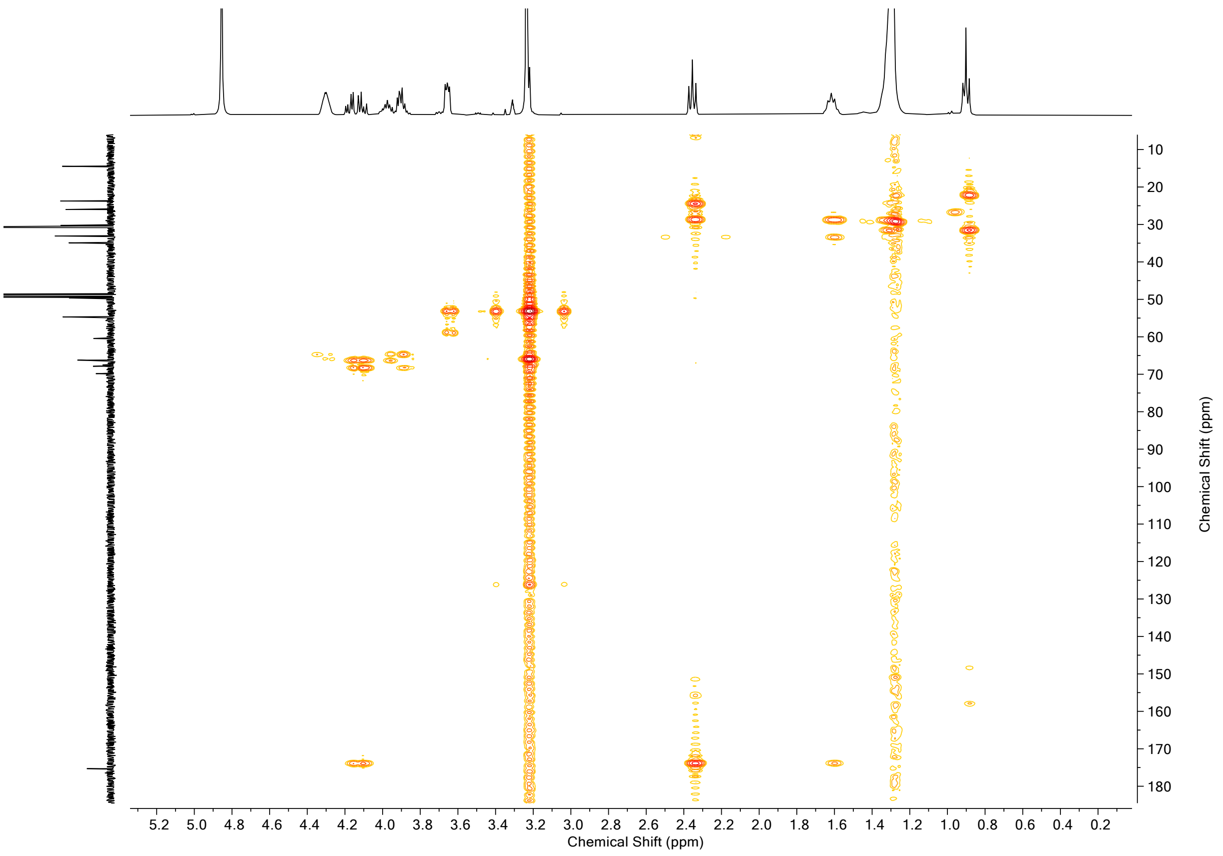

Figure S18: HMBC spectrum of **PC** (CD_3OD).

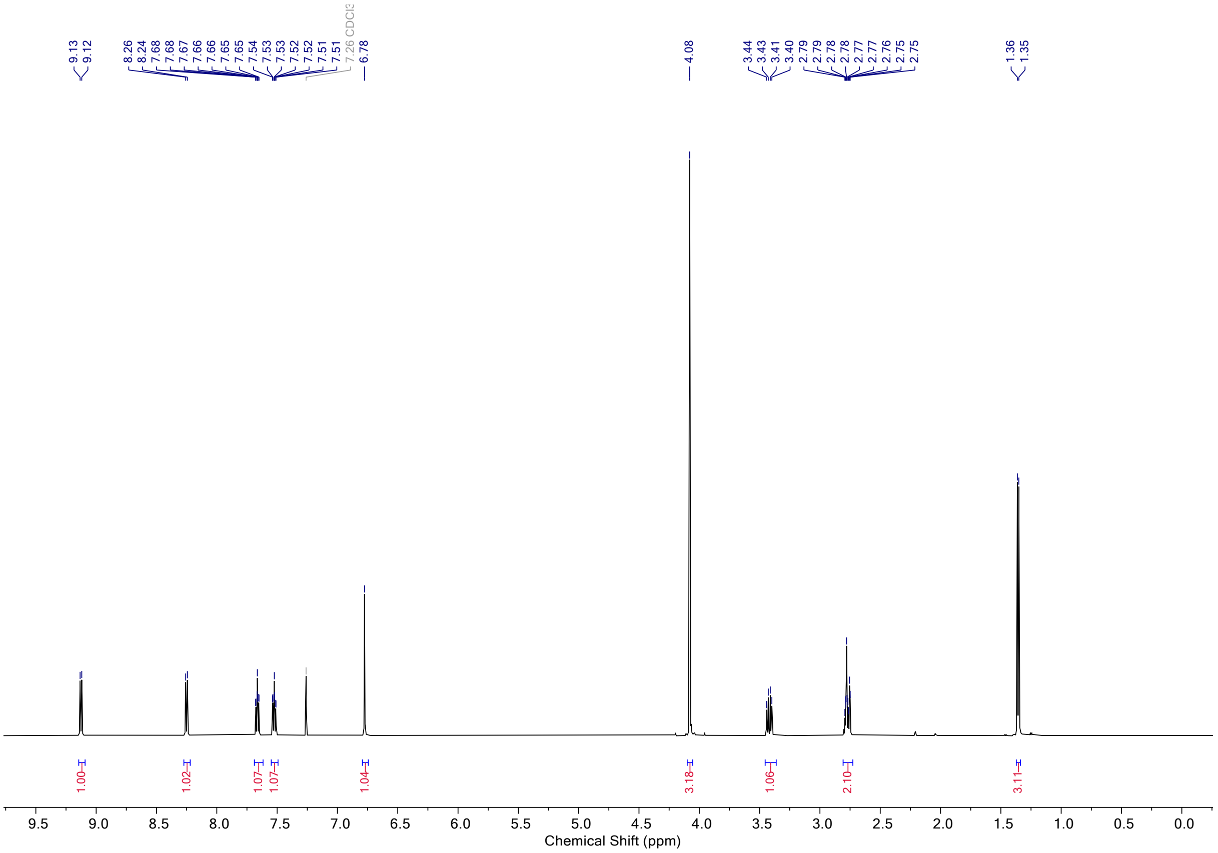

Figure S19: **^{1}H NMR** spectrum of **1** ($CDCl_3$).

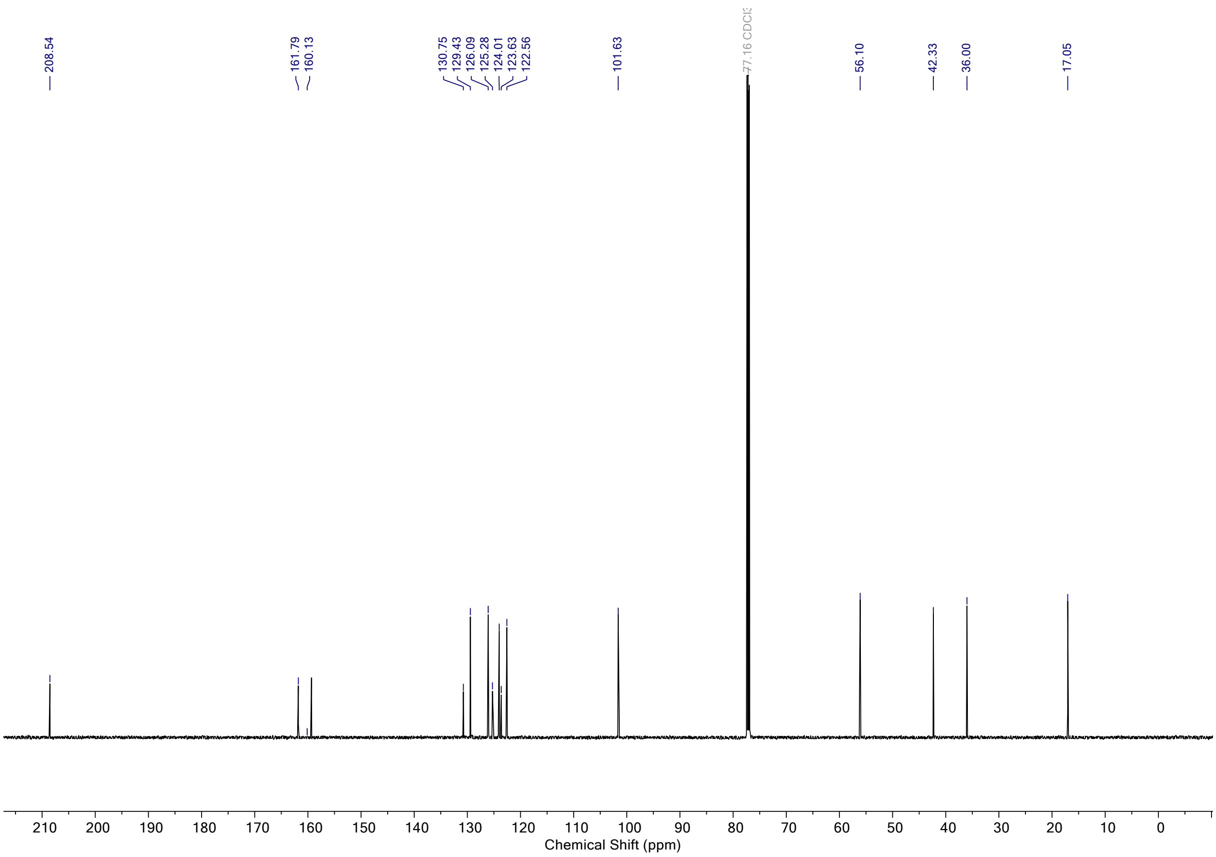

Figure S20: $^{13}C\{^{1}H\}$ **NMR** spectrum of **1** ($CDCl_3$).

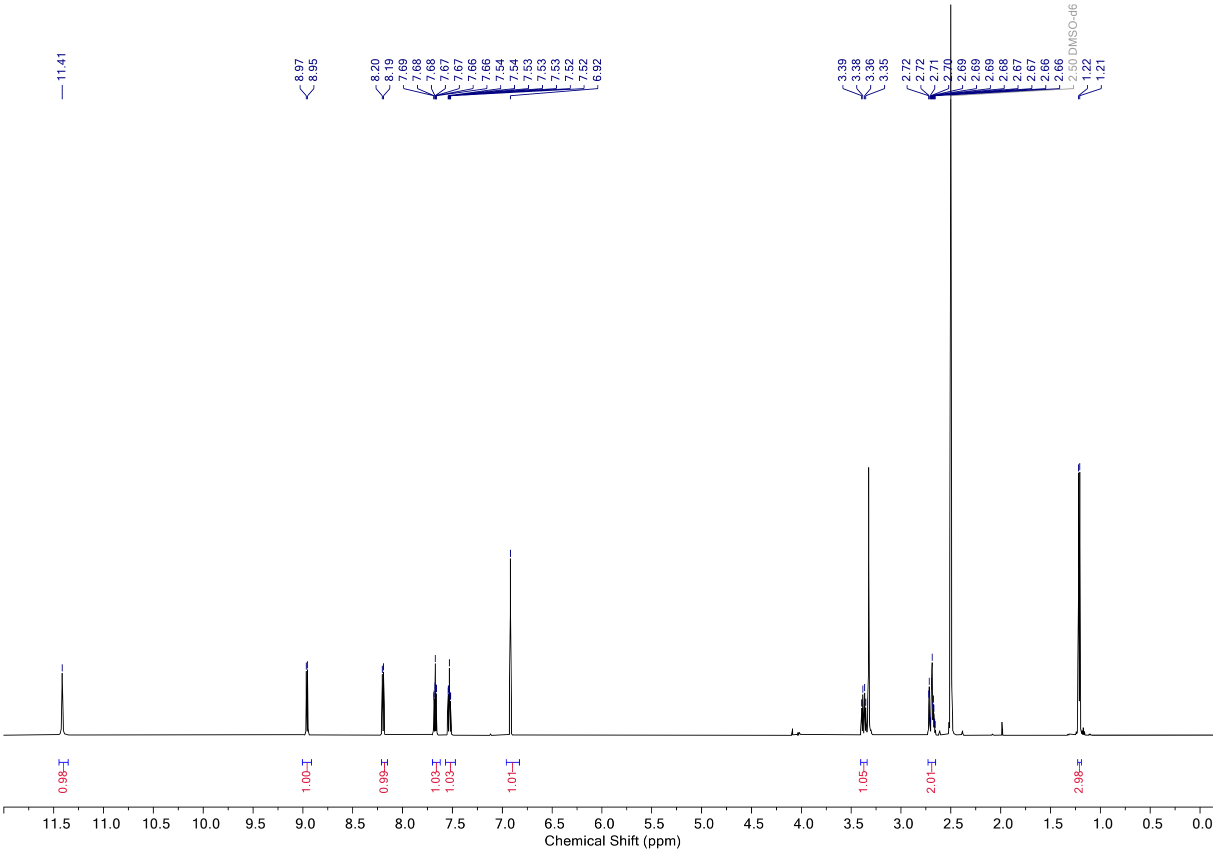

Figure S21: ^{1}H **NMR** spectrum of **2** ($(CD_3)_2SO$).

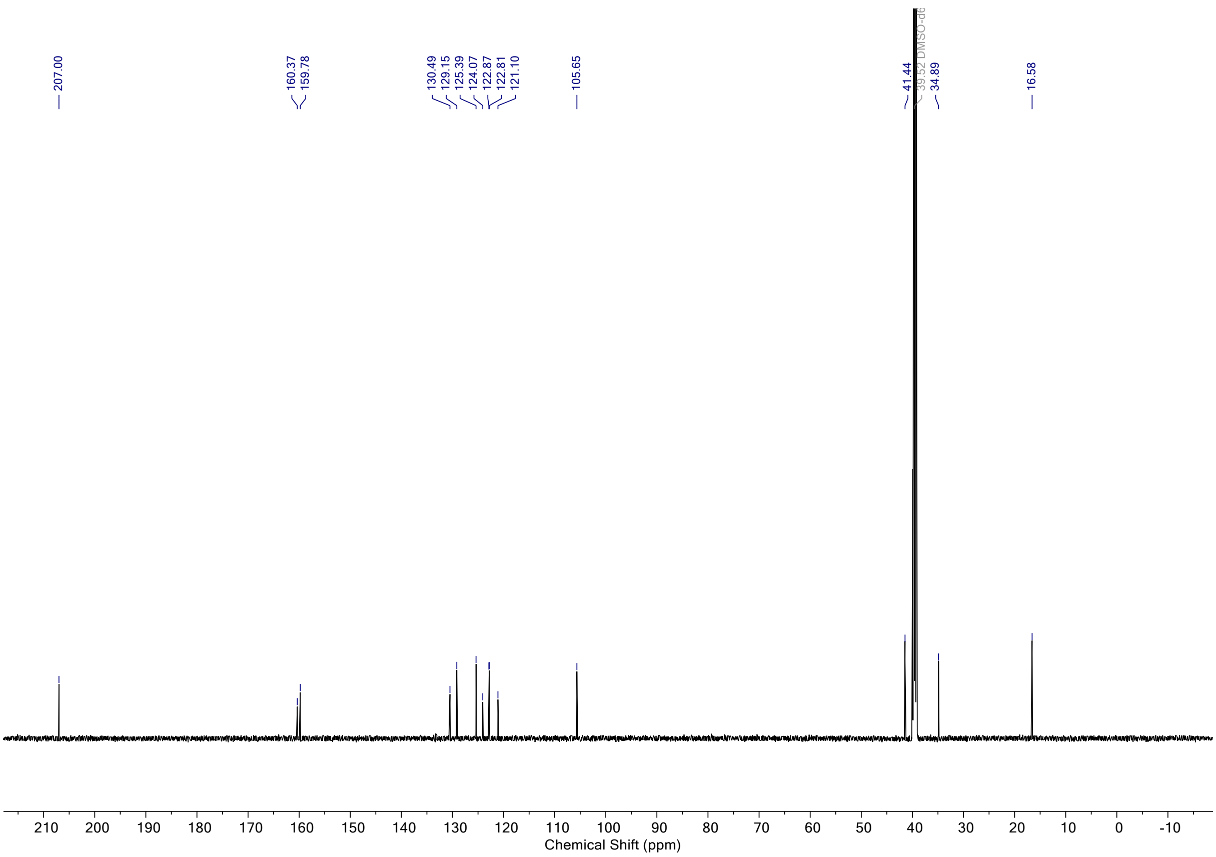

Figure S22: $^{13}C\{^{1}H\}$ **NMR** spectrum of **2** ($(CD_3)_2SO$).

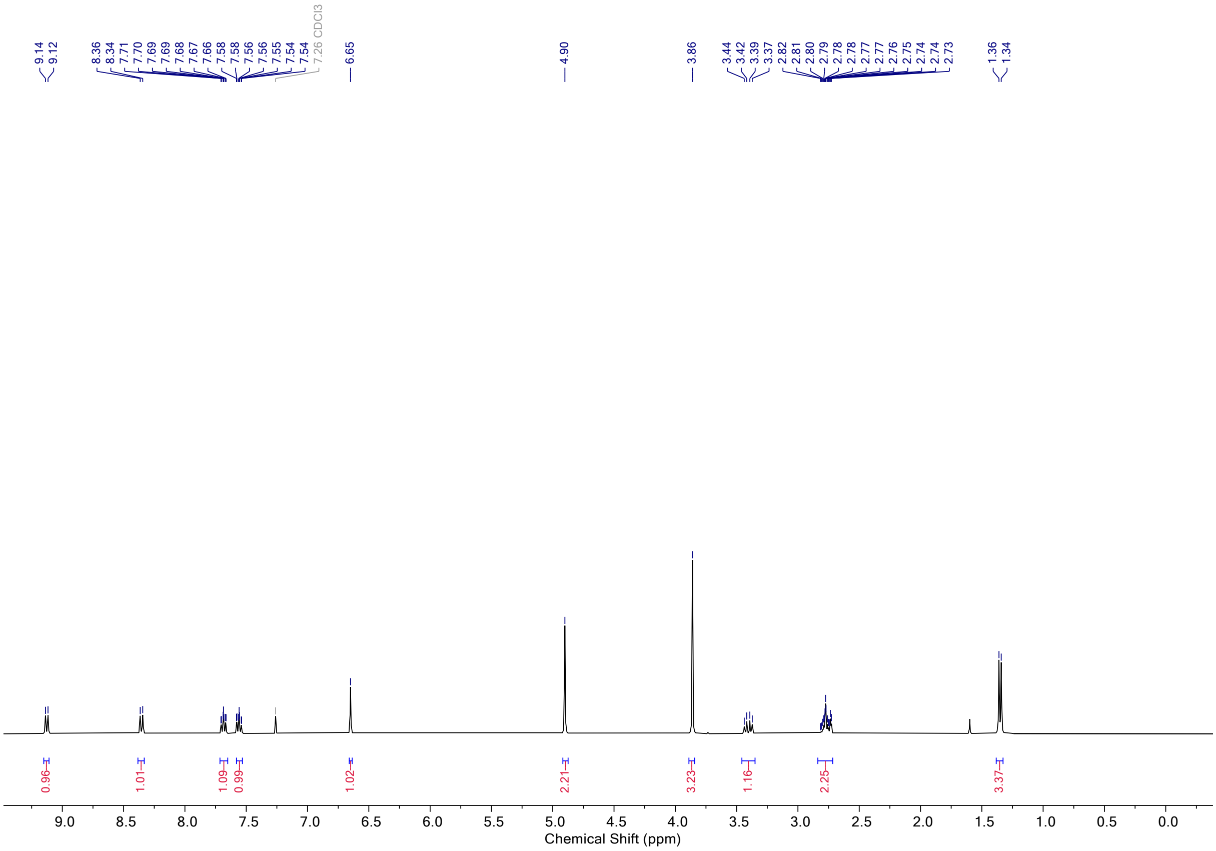

Figure S23: ^{1}H **NMR** spectrum of **3a** ($CDCl_3$).

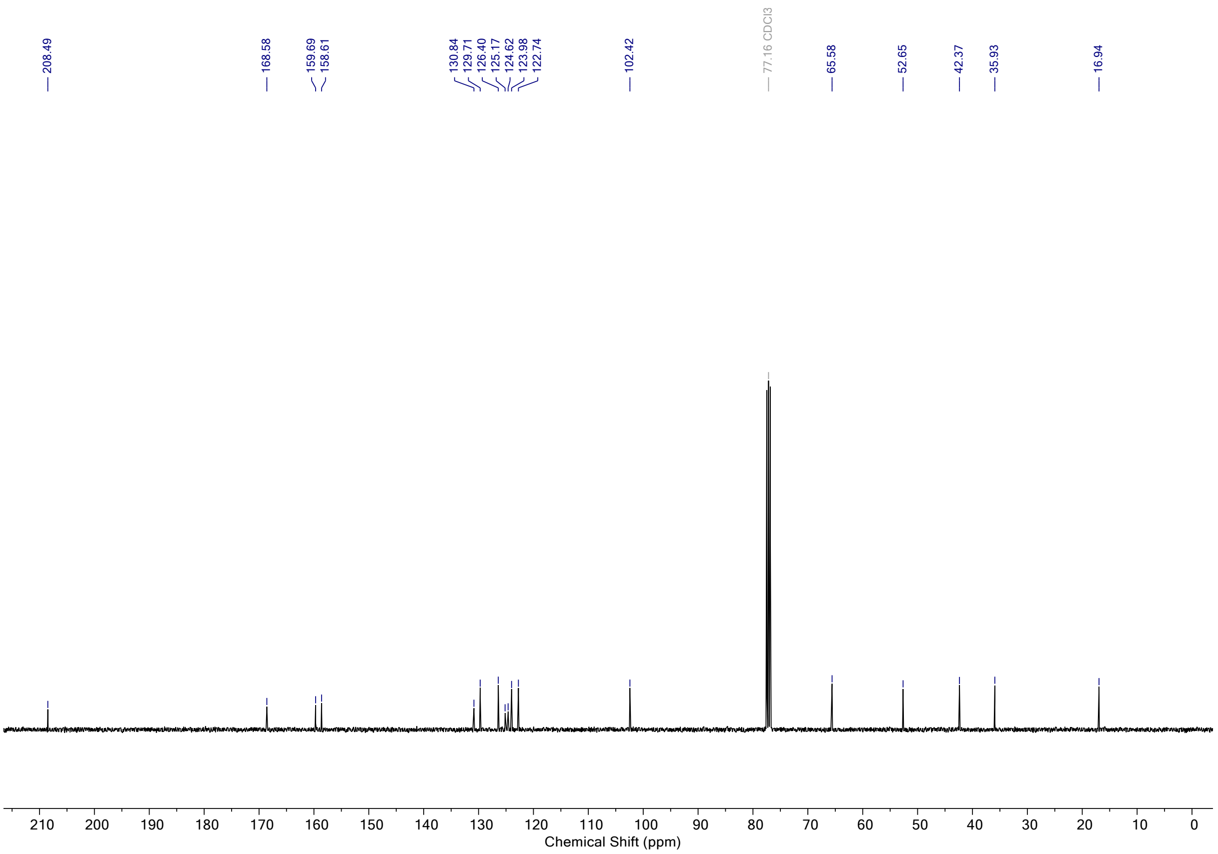

Figure S24: $^{13}C\{^1H\}$ **NMR** spectrum of **3a** ($CDCl_3$).

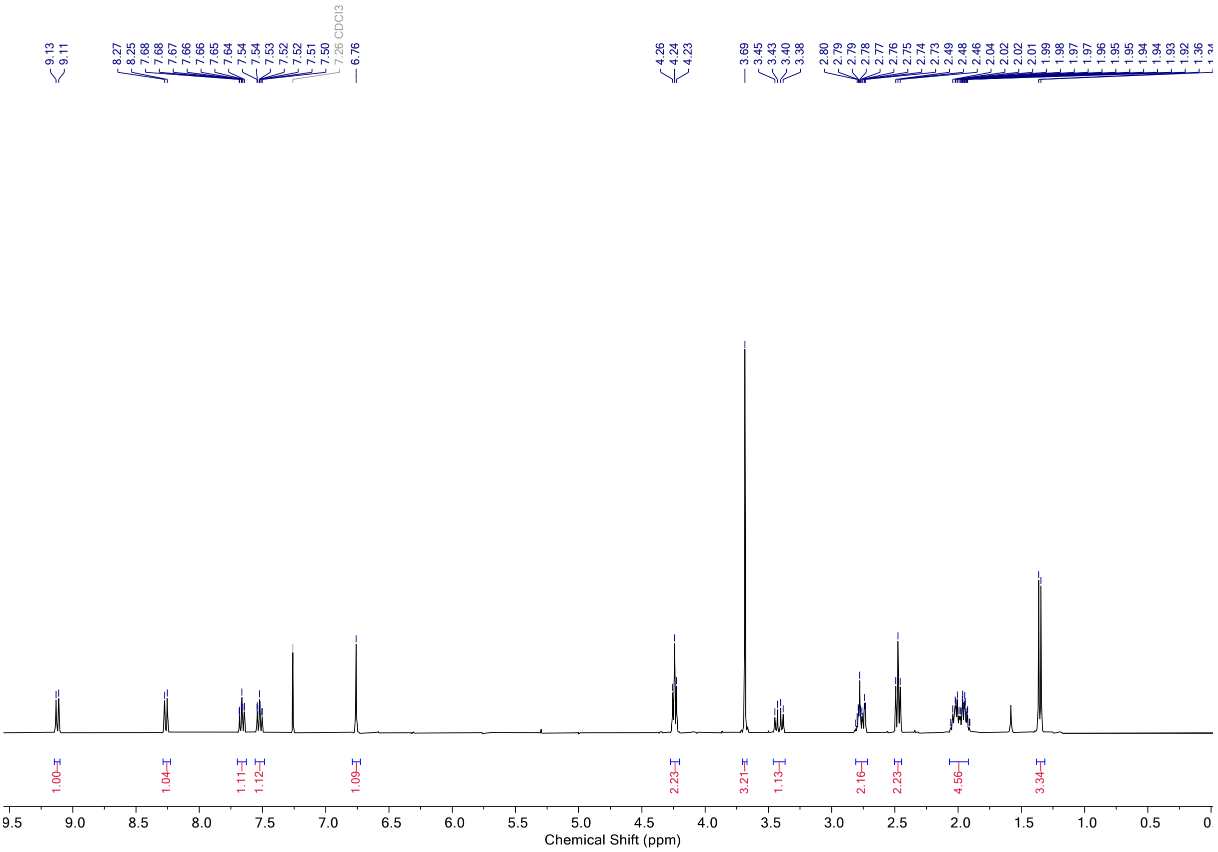

Figure S25: ^{1}H **NMR** spectrum of **3b** ($CDCl_3$).

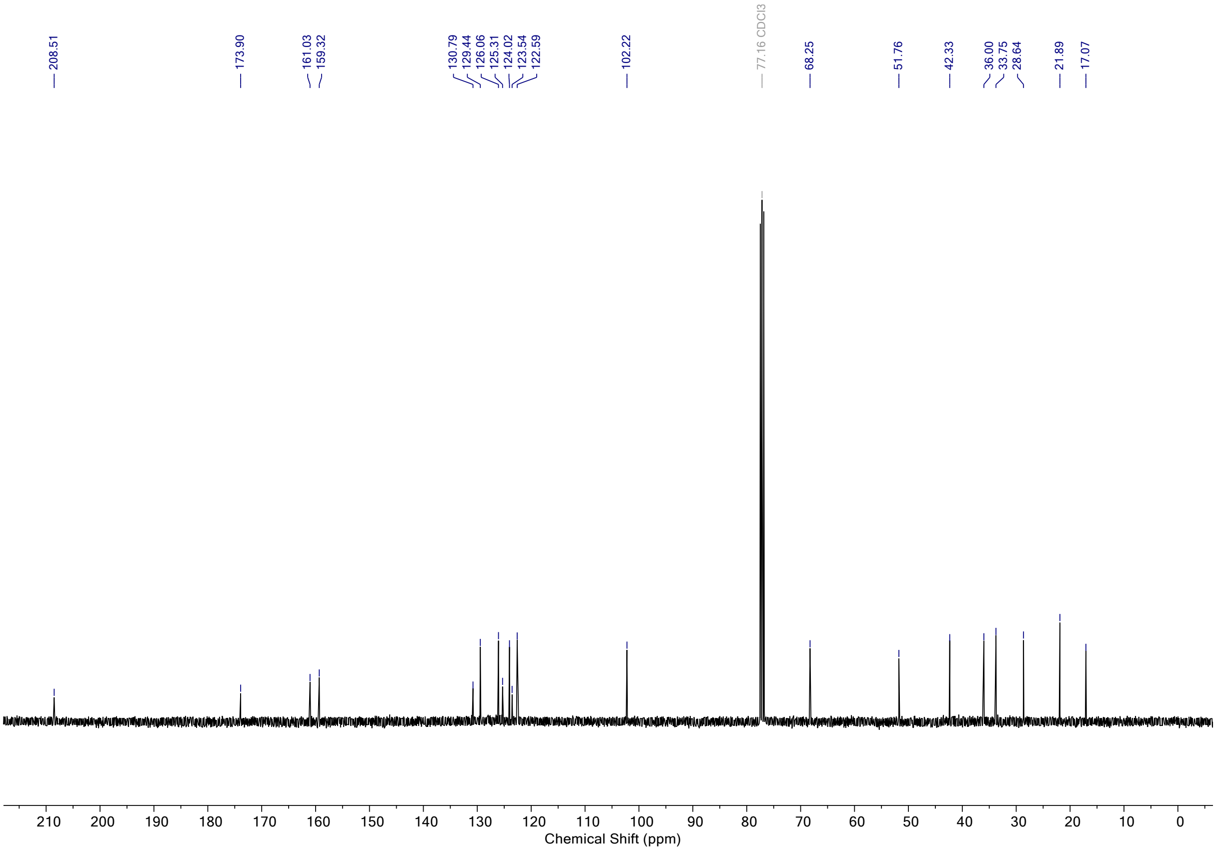

Figure S26: $^{13}C\{^{1}H\}$ **NMR** spectrum of **3b** ($CDCl_3$).

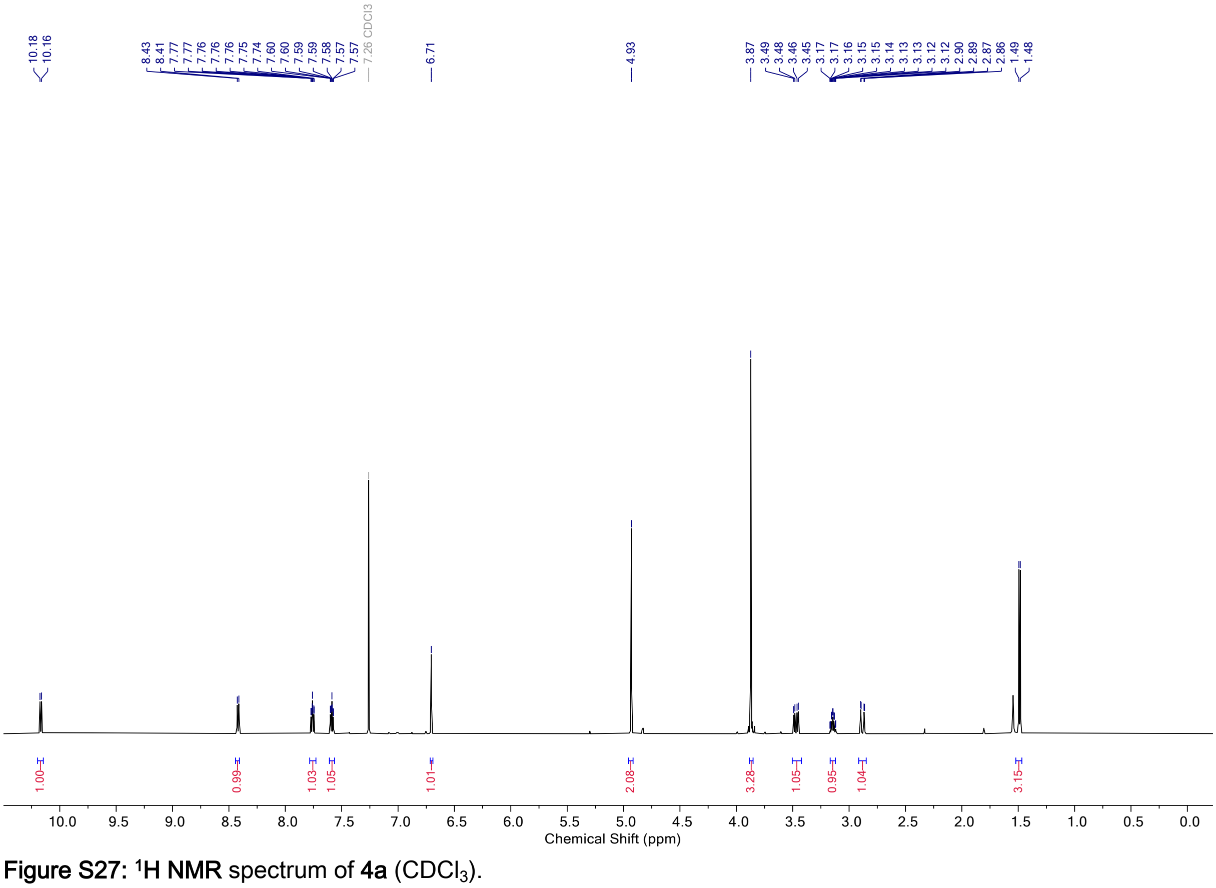

Figure S27: ^{1}H **NMR** spectrum of **4a** ($CDCl_3$).

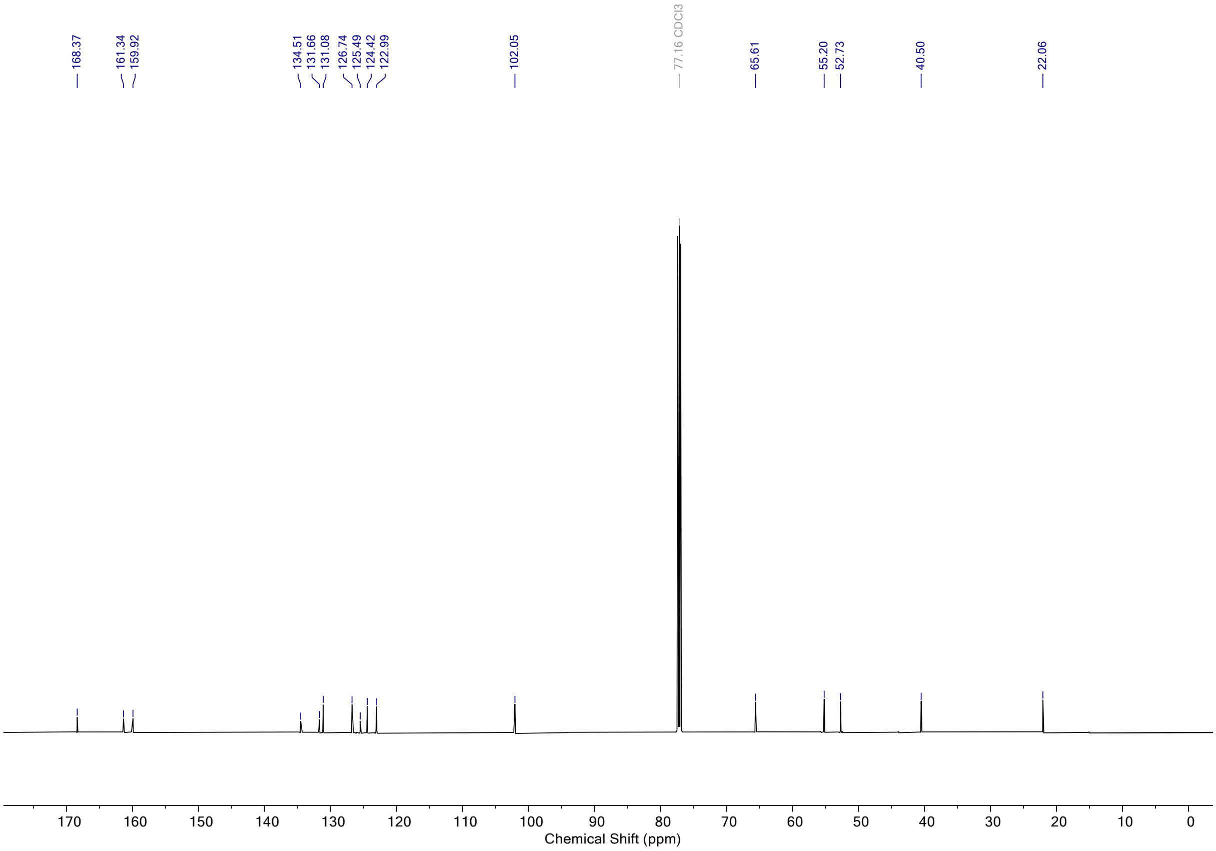

Figure S28: $^{13}C\{^{1}H\}$ **NMR** spectrum of **4a** ($CDCl_3$).

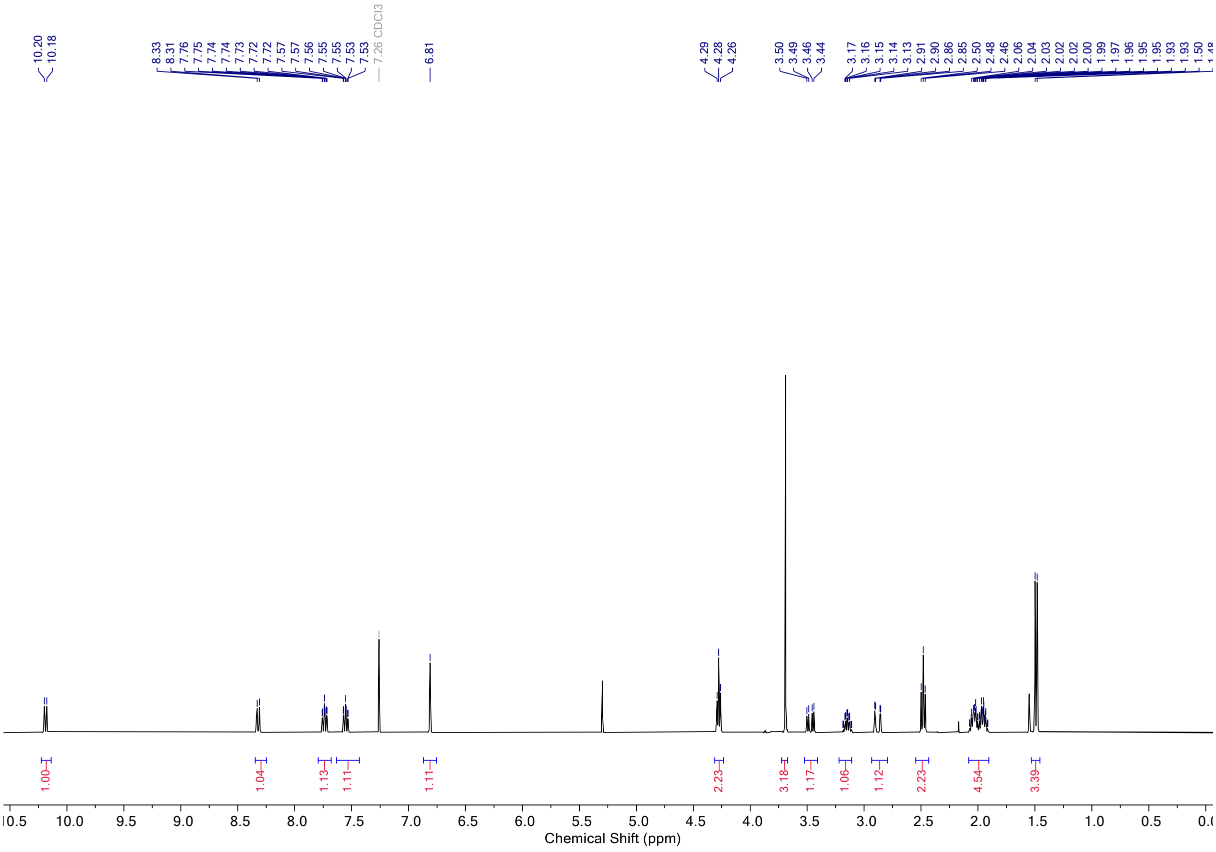

Figure S29: ^{1}H **NMR** spectrum of **4b** ($CDCl_3$).

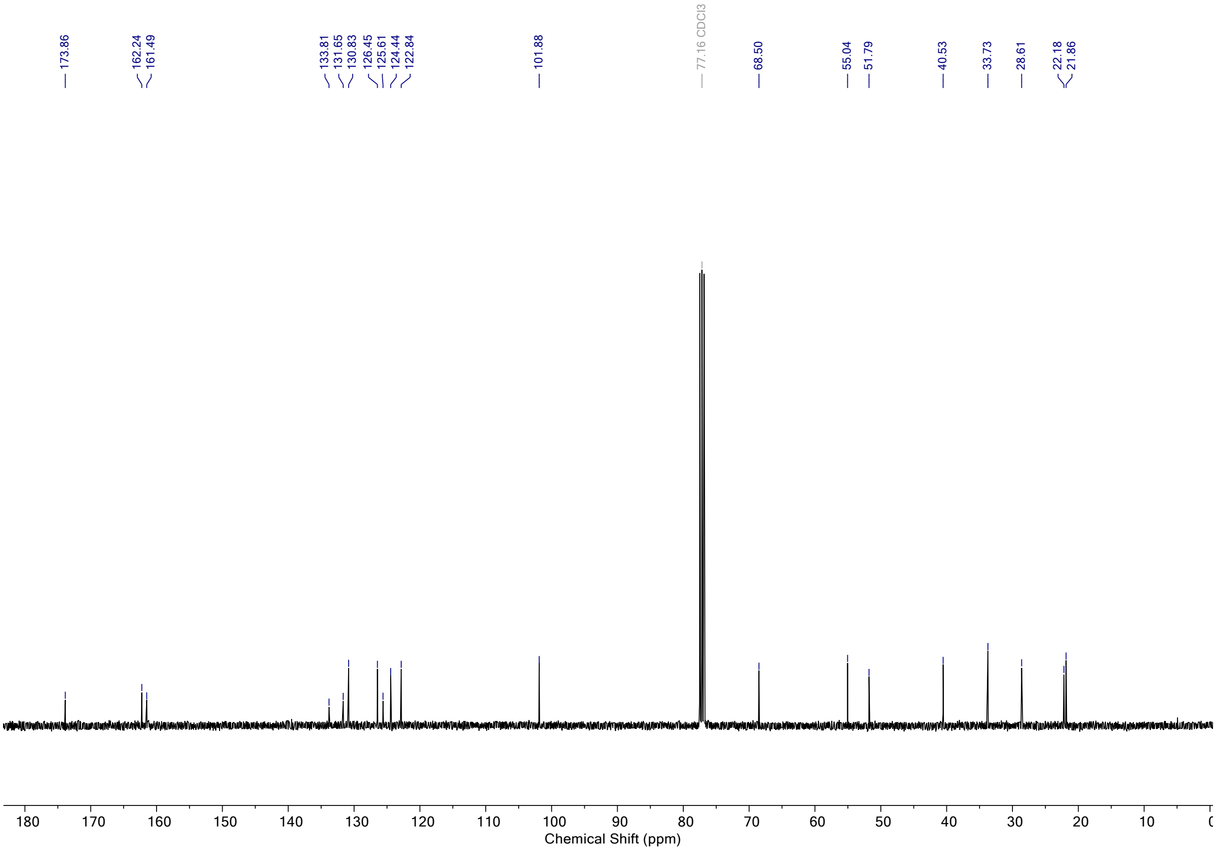

Figure S30: ^{13}C{^{1}H} **NMR** spectrum of **4b** ($CDCl_3$).

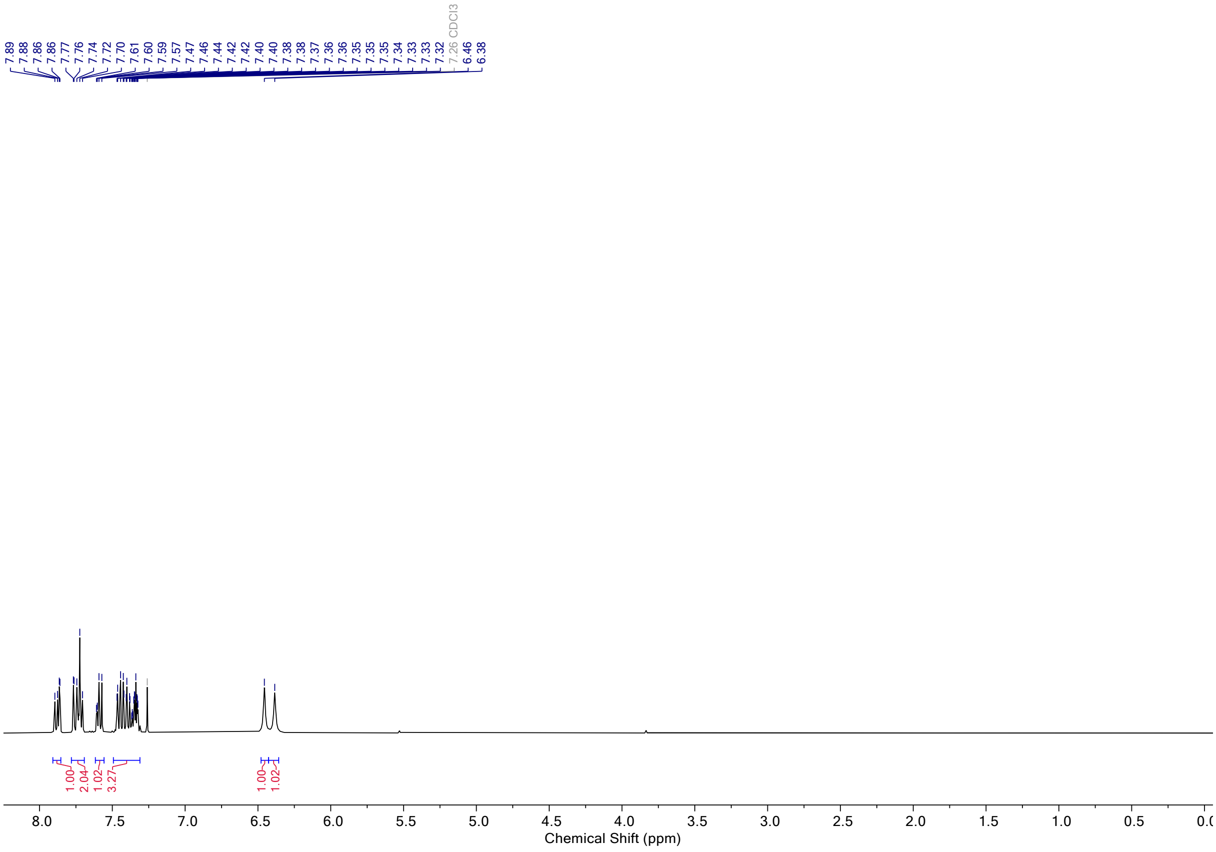

Figure S31: ^{1}H **NMR** spectrum of **5a** ($CDCl_3$).

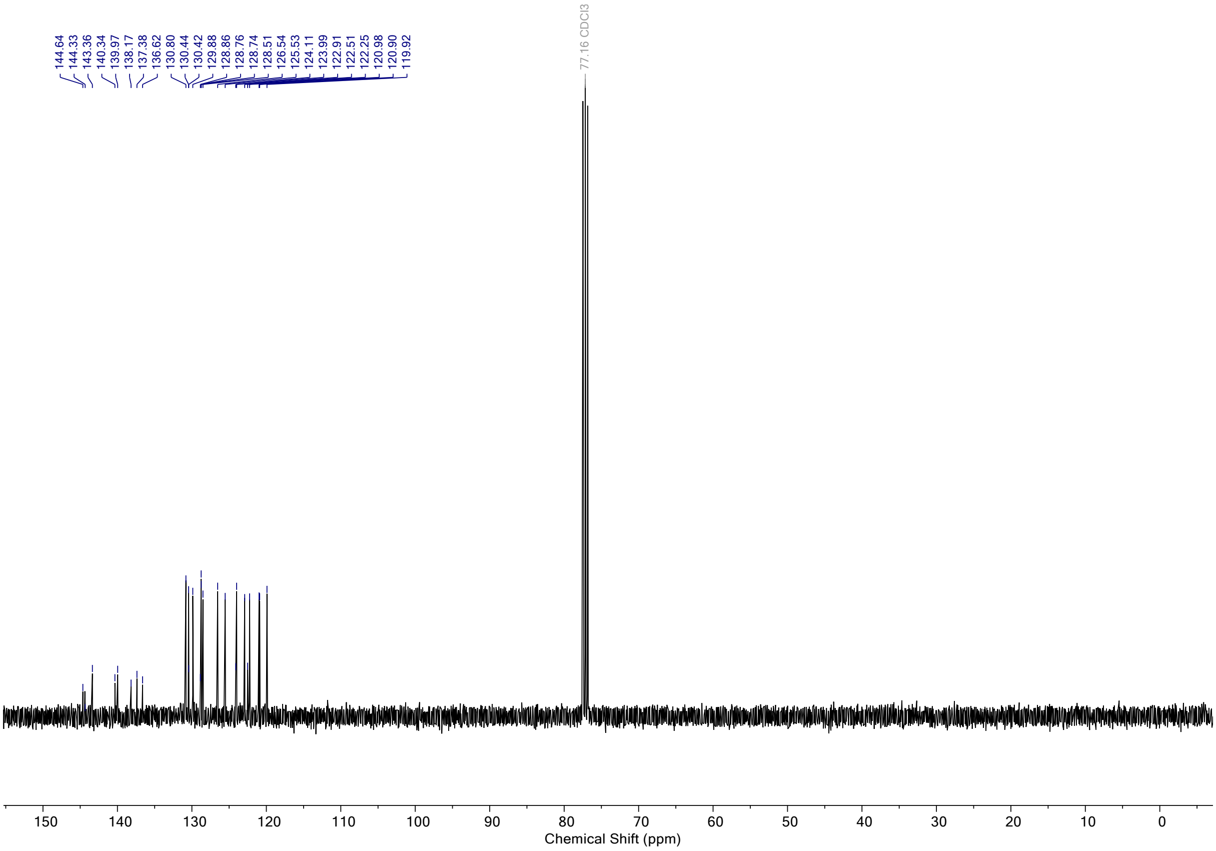

Figure S32: $^{13}C\{^1H\}$ **NMR** spectrum of **5a** ($CDCl_3$).

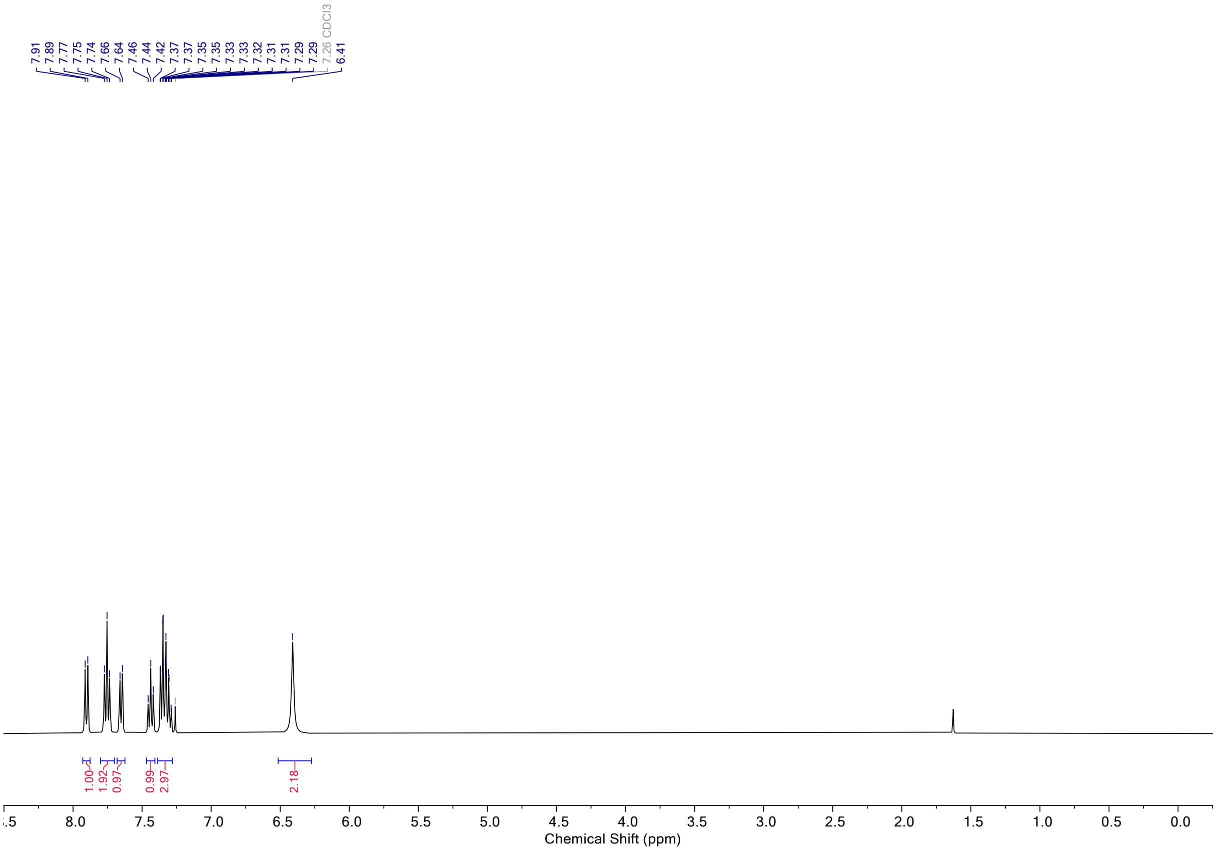

Figure S33: ^1H **NMR** spectrum of **5b** ($CDCl_3$).

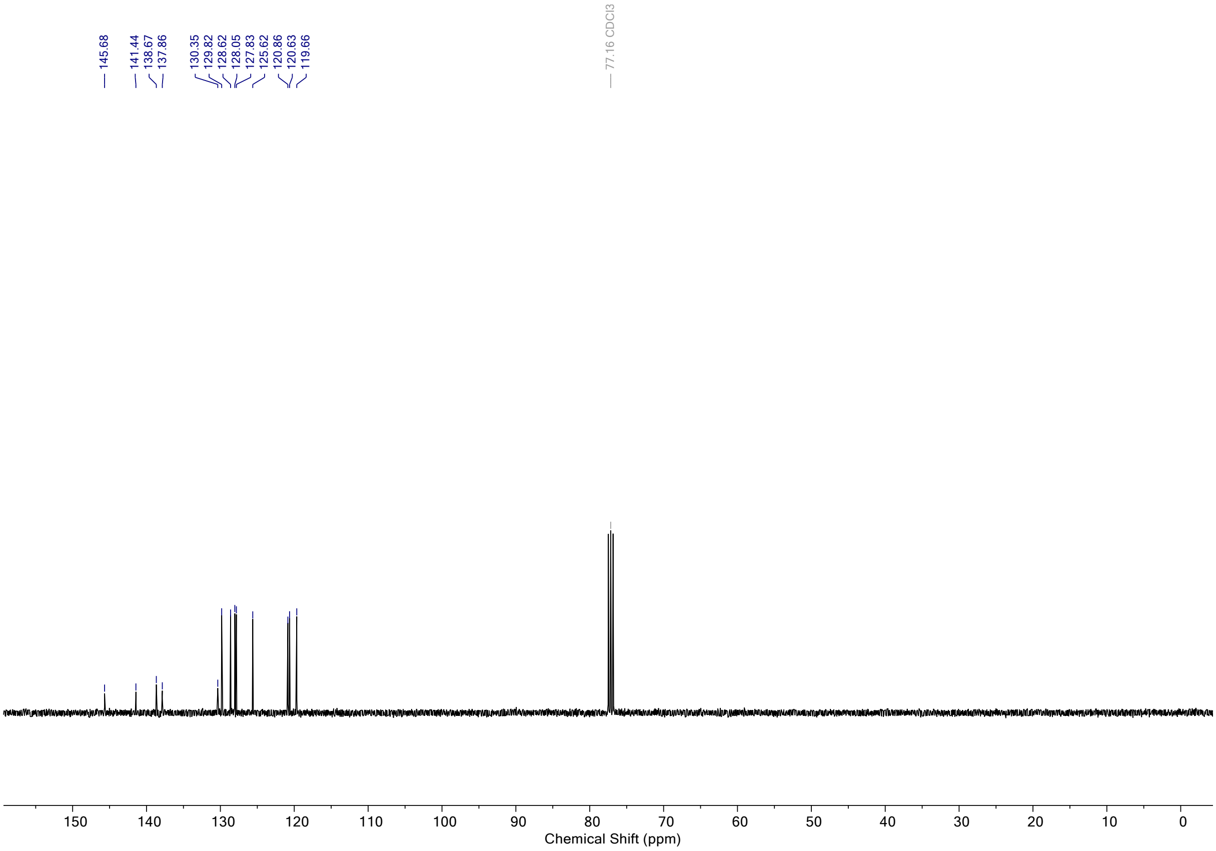

Figure S34: $^{13}C\{^{1}H\}$ **NMR** spectrum of **5b** ($CDCl_3$).

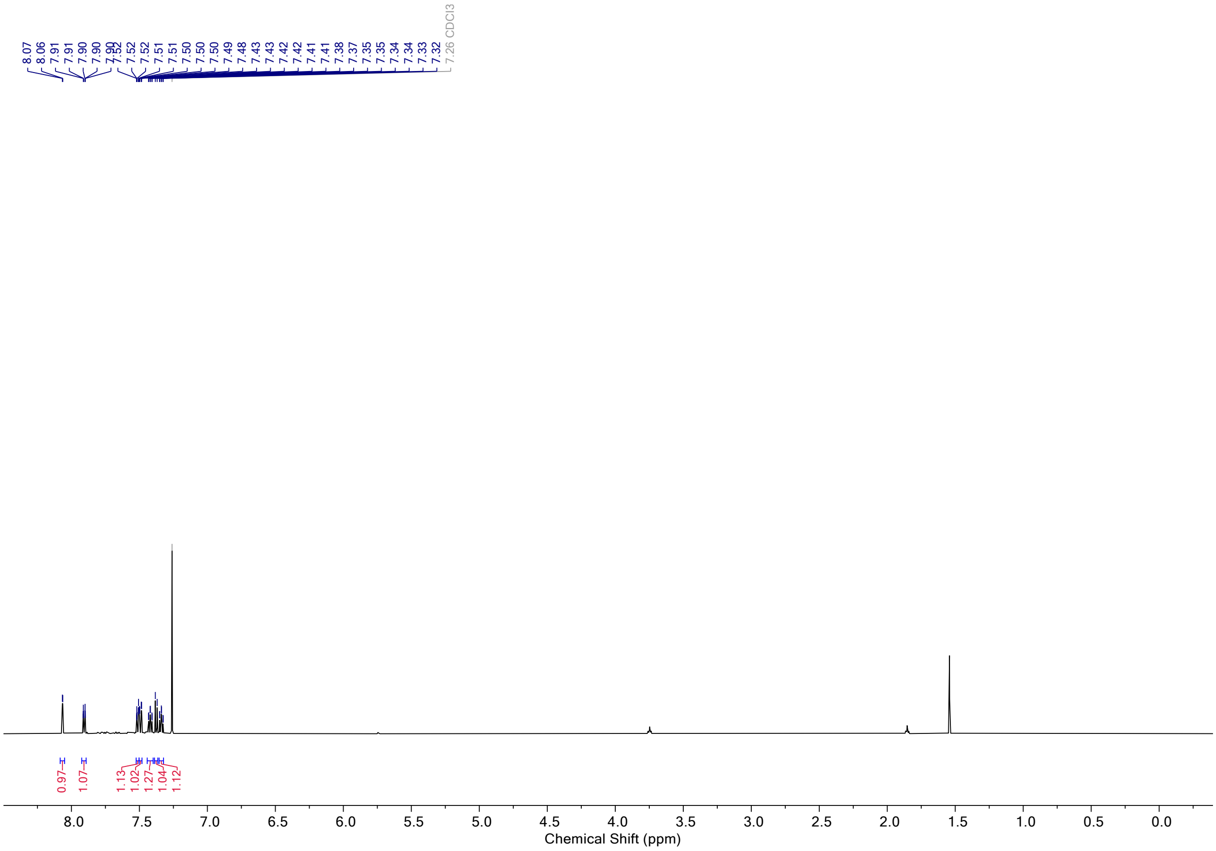

Figure S35: ^{1}H **NMR** spectrum of **6a** ($CDCl_3$).

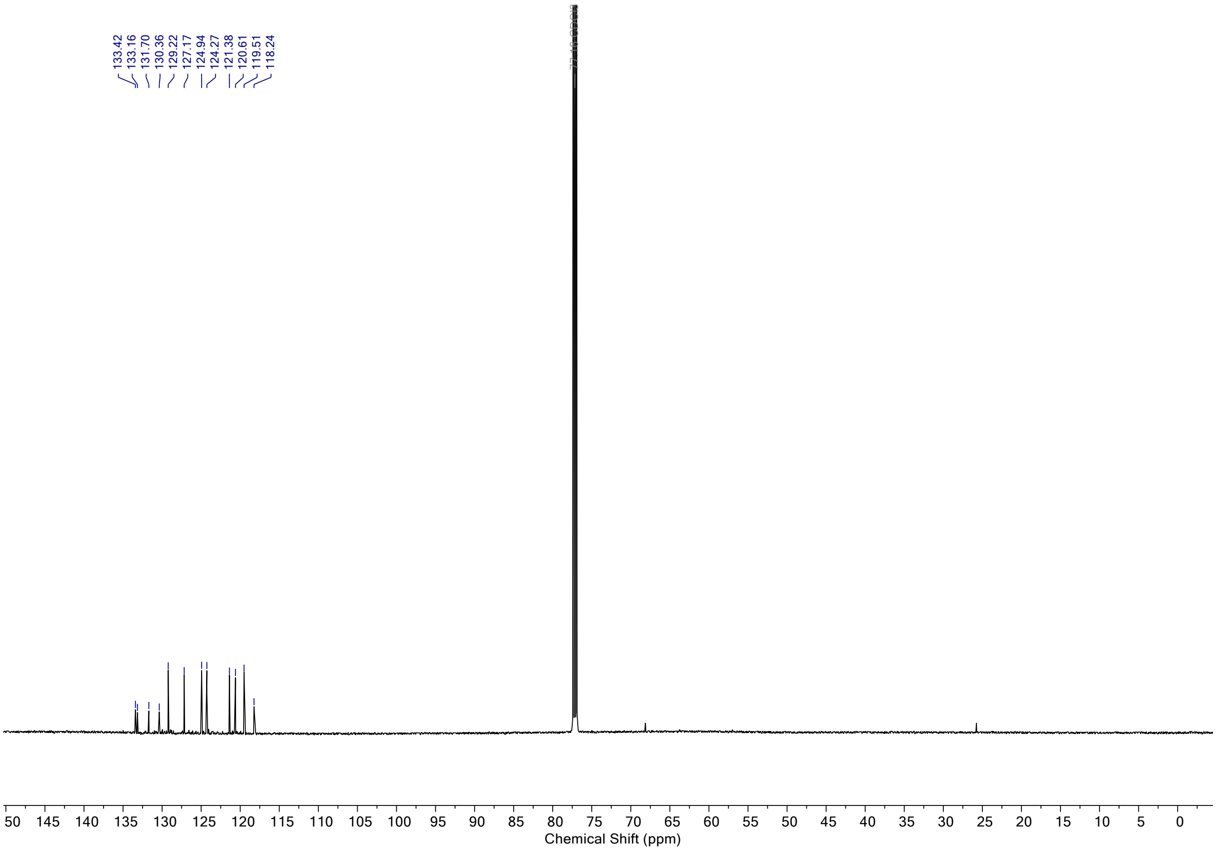

Figure S36: $^{13}C\{^{1}H\}$ **NMR** spectrum of **6a** ($CDCl_3$).

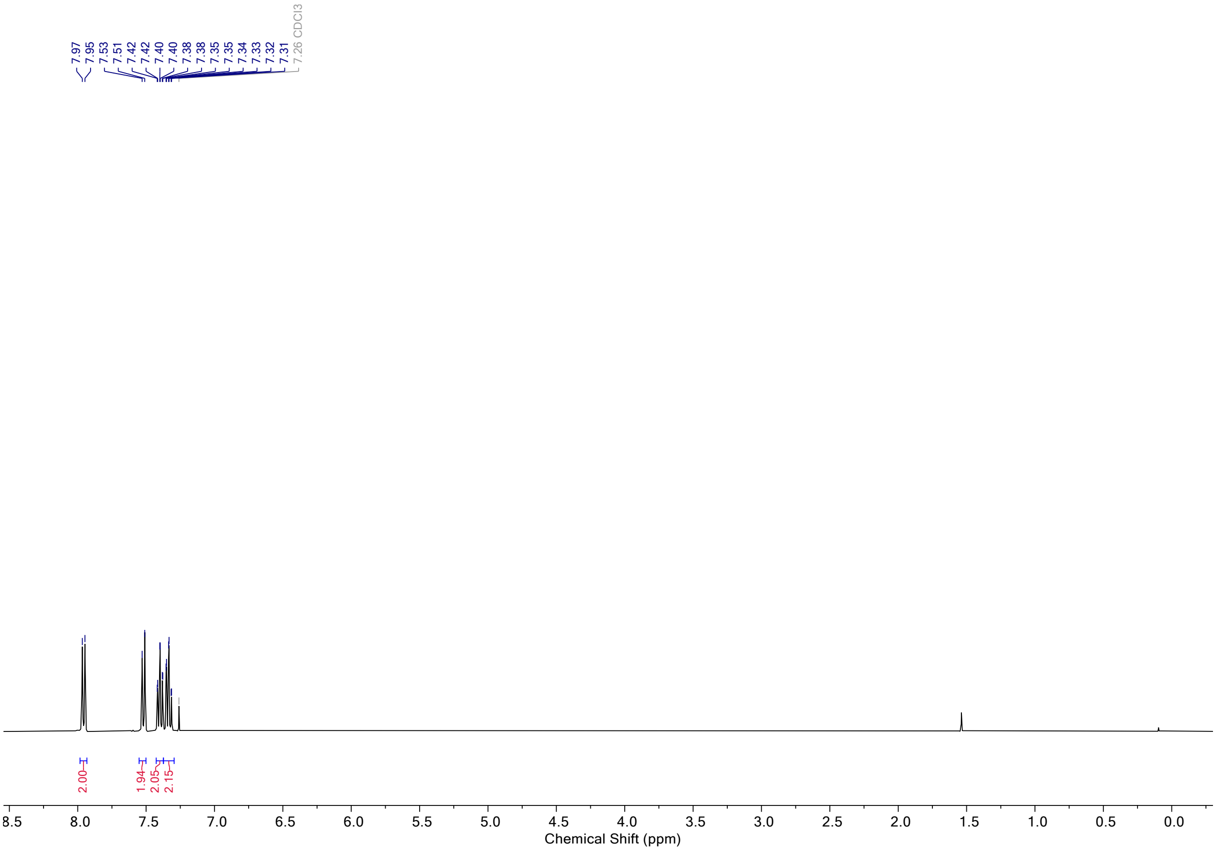

Figure S37: ^{1}H **NMR** spectrum of **6b** ($CDCl_3$).

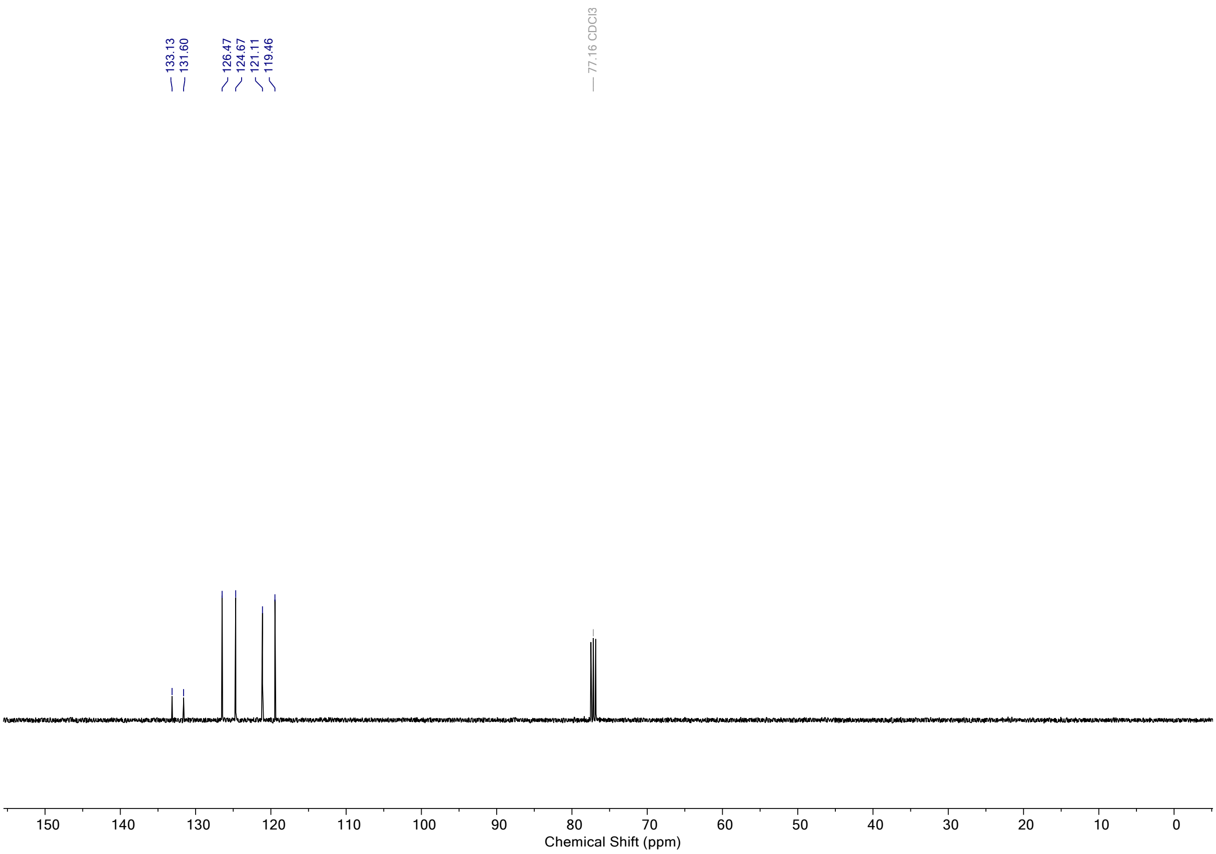

Figure S38: ^{13}**C{**^{1}**H} NMR** spectrum of **6b** ($CDCl_3$).

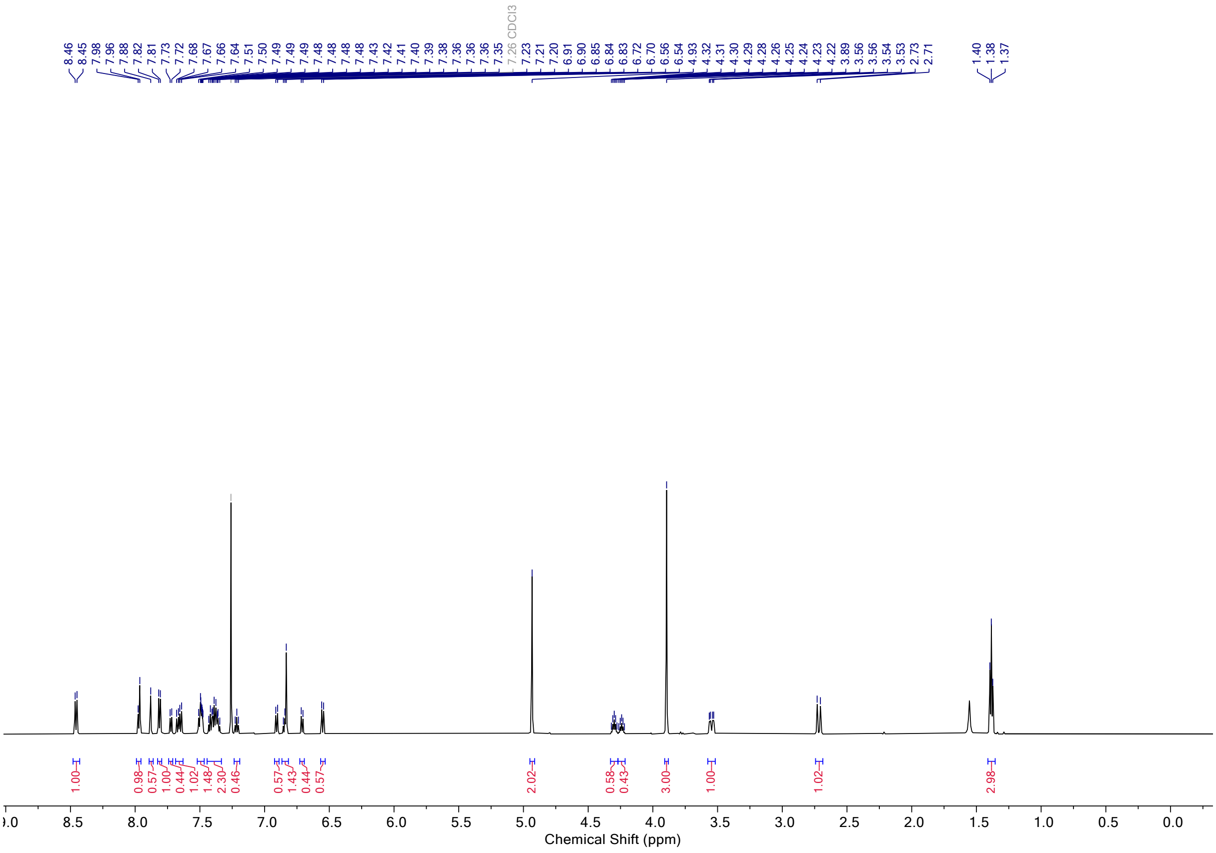

Figure S39: ^{1}**H NMR** spectrum of **7a** ($CDCl_3$).

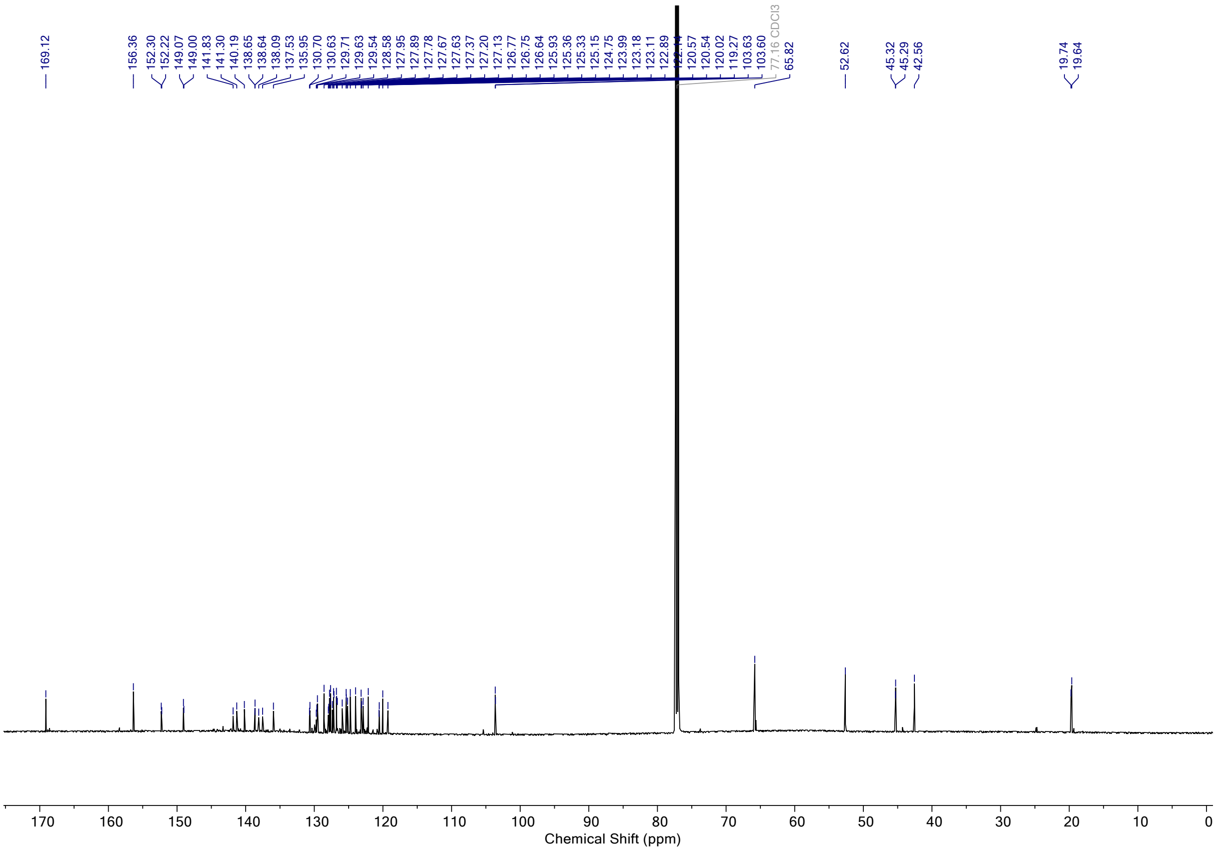

Figure S40: $^{13}C\{^1H\}$ **NMR** spectrum of **7a** ($CDCl_3$).

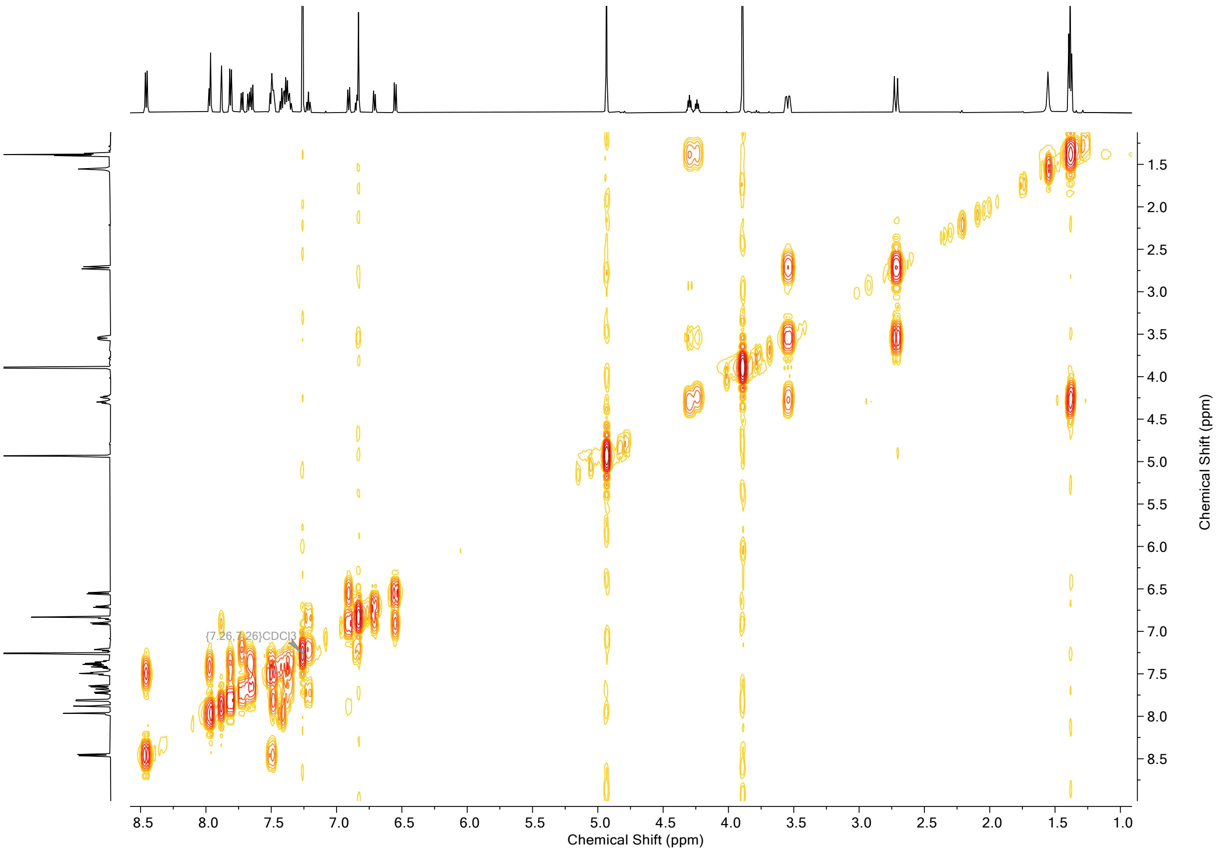

Figure S41: COSY spectrum of **7a** ($CDCl_3$).

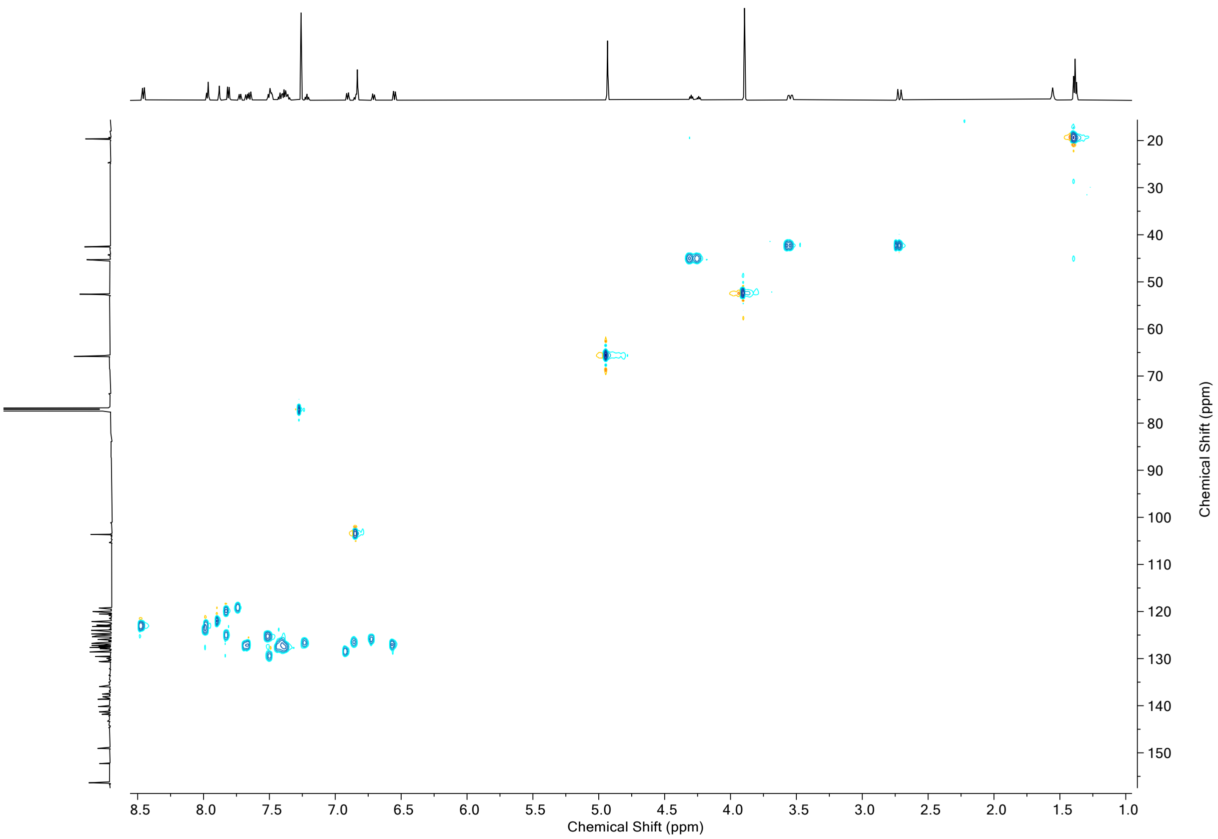

Figure S42: HSQC spectrum of **7a** ($CDCl_3$).

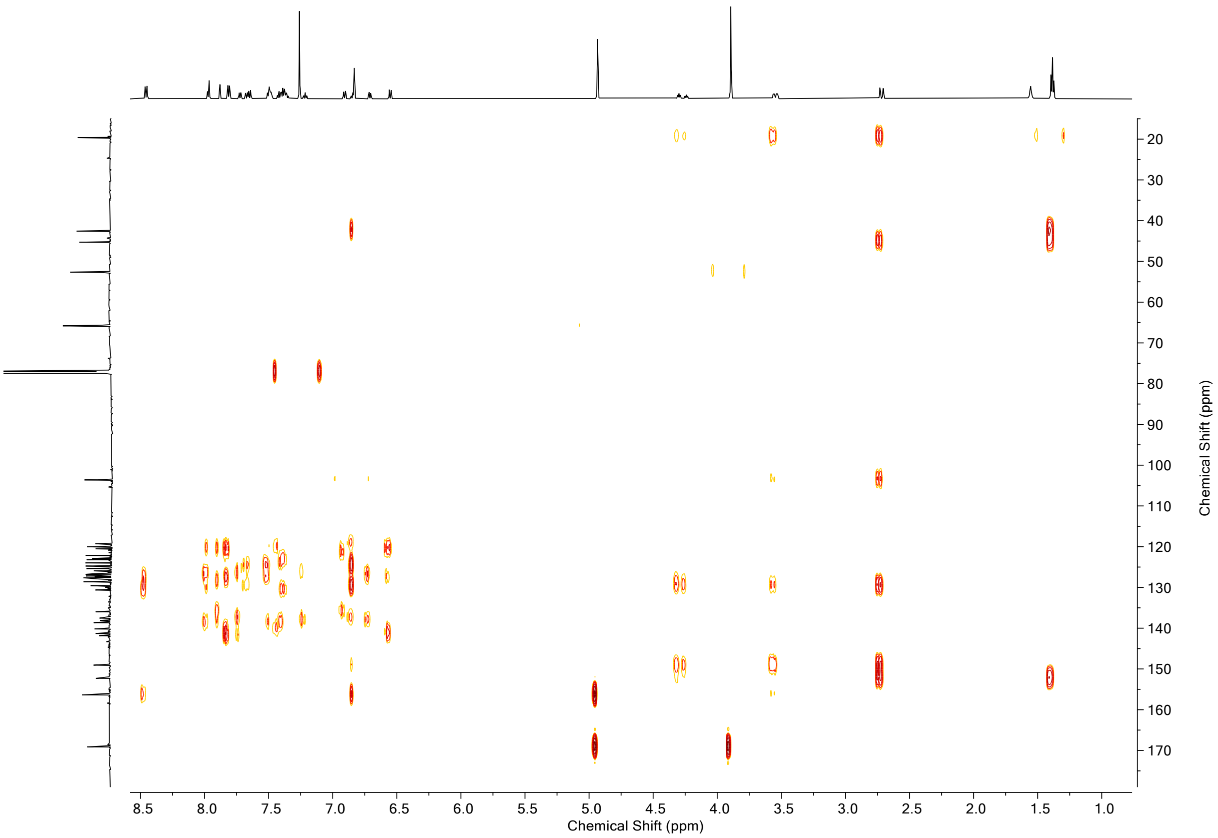

Figure S43: HMBC spectrum of **7a** ($CDCl_3$).

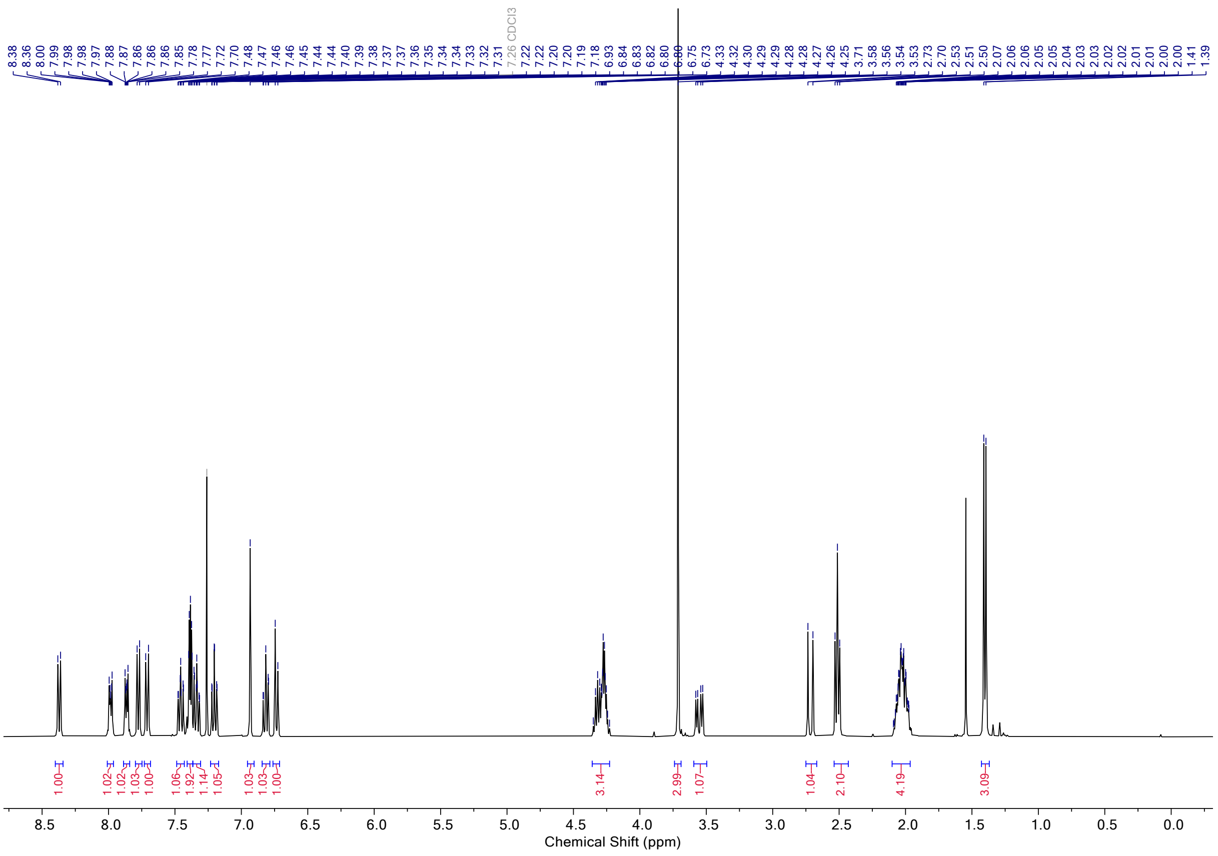

Figure S44: ¹**H NMR** spectrum of **7b** ($CDCl_3$).

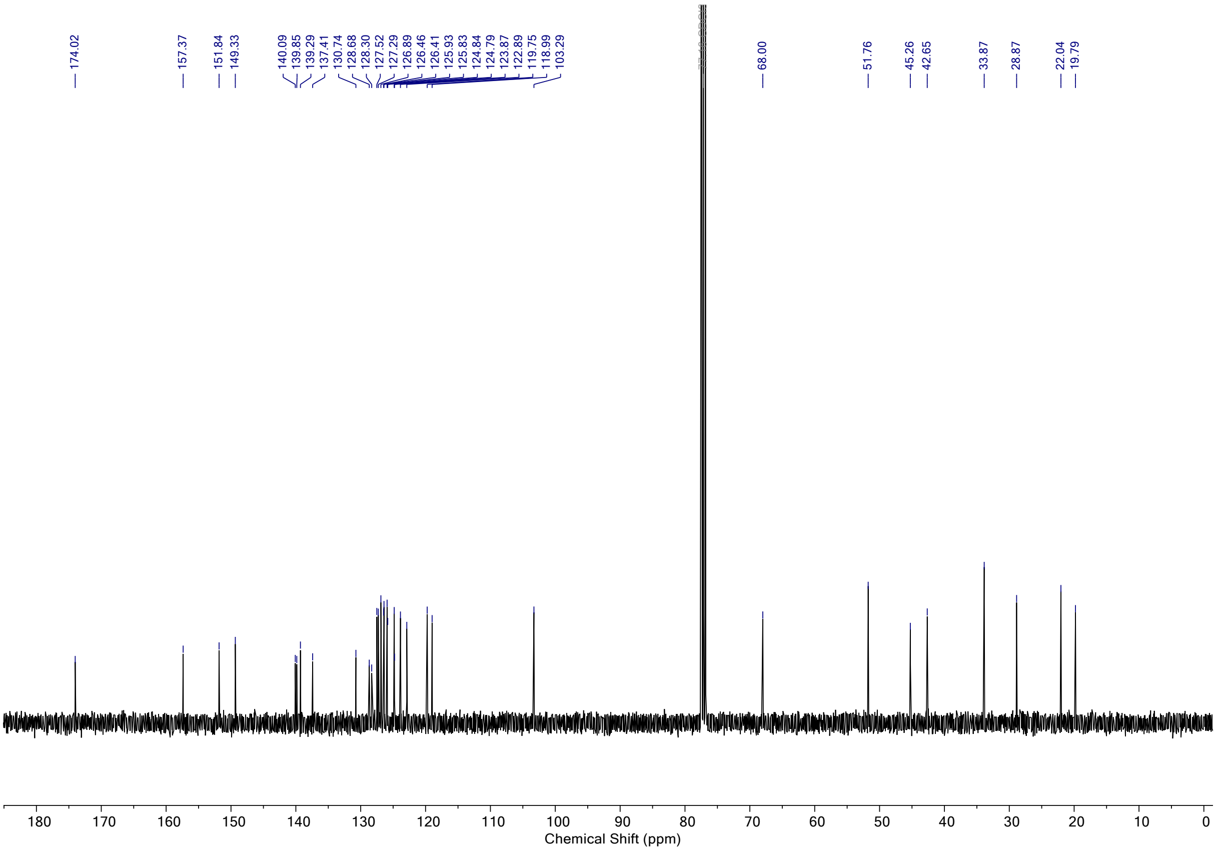

Figure S45: ^{13}**C{¹H} NMR** spectrum of **7b** ($CDCl_3$).

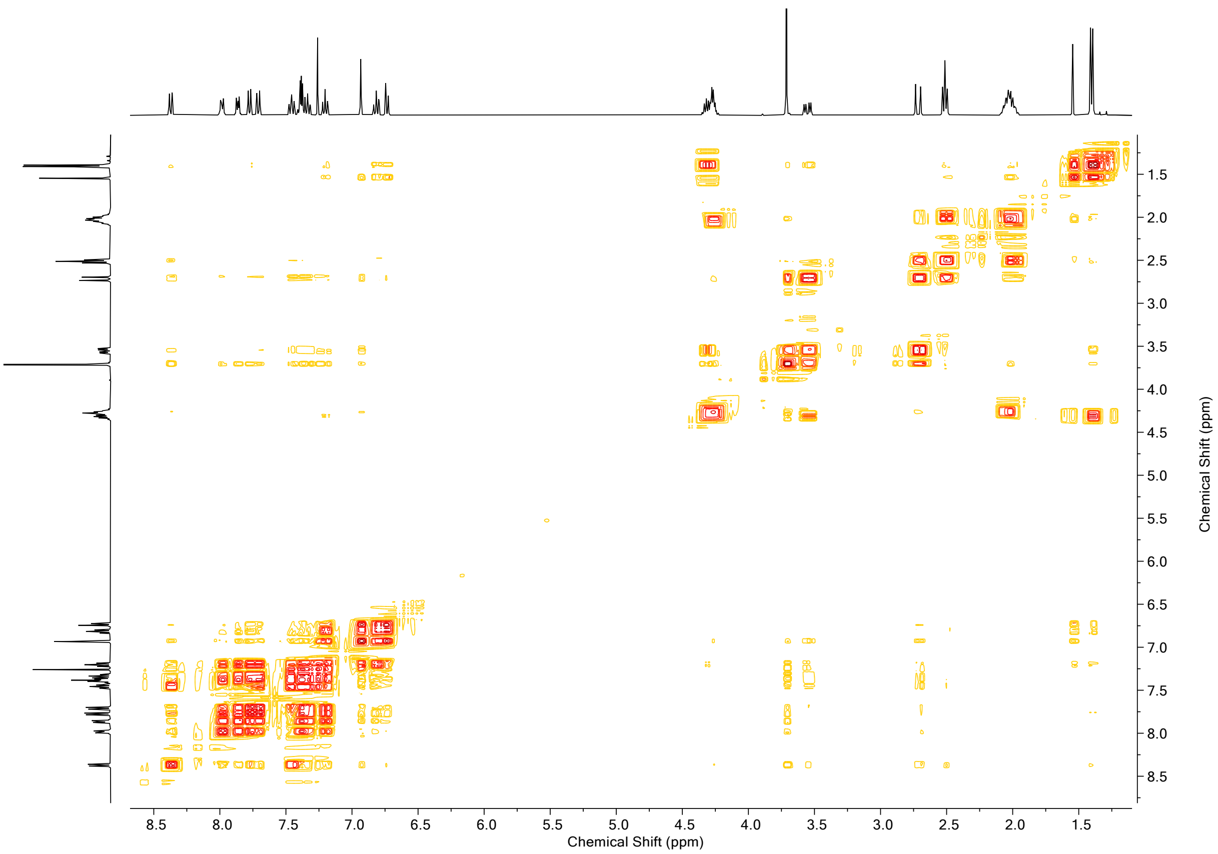

Figure S46: COSY spectrum of **7b** ($CDCl_3$).

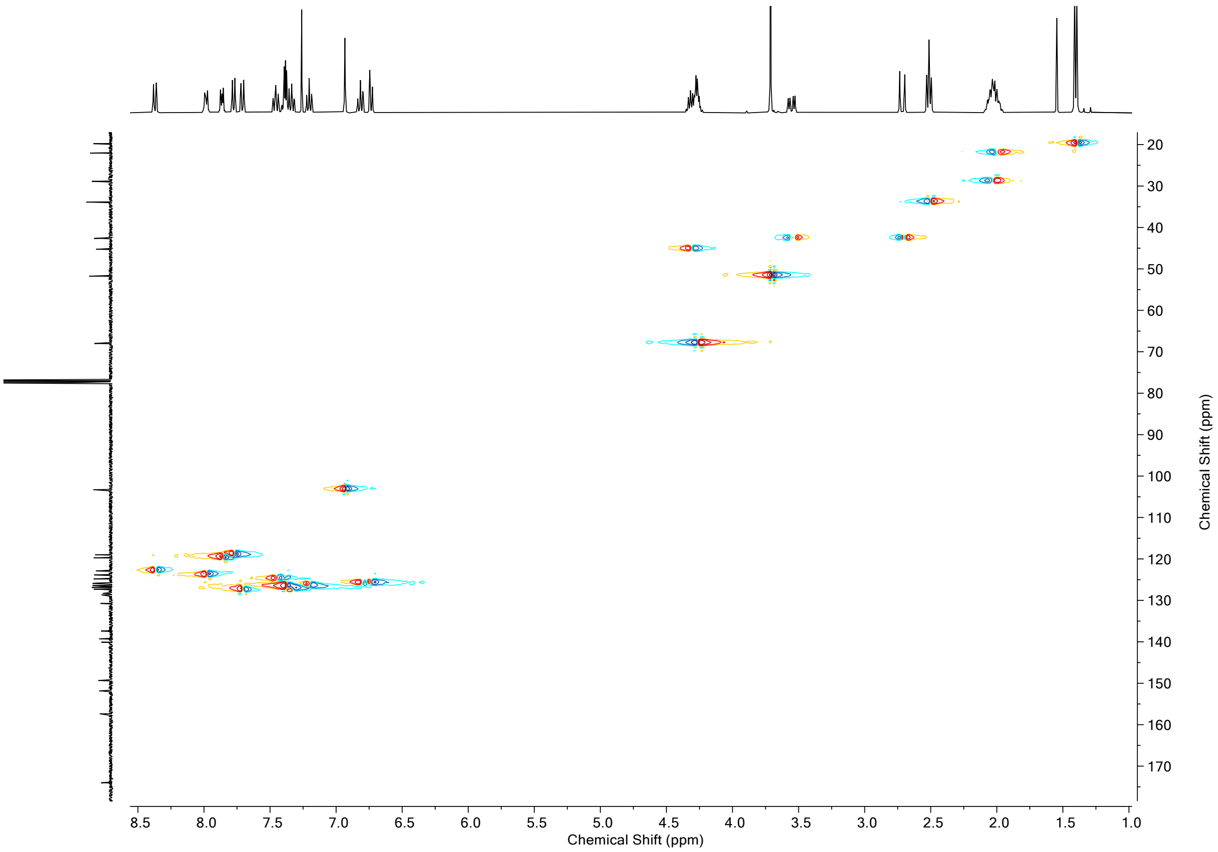

Figure S47: HSQC spectrum of **7b** ($CDCl_3$).

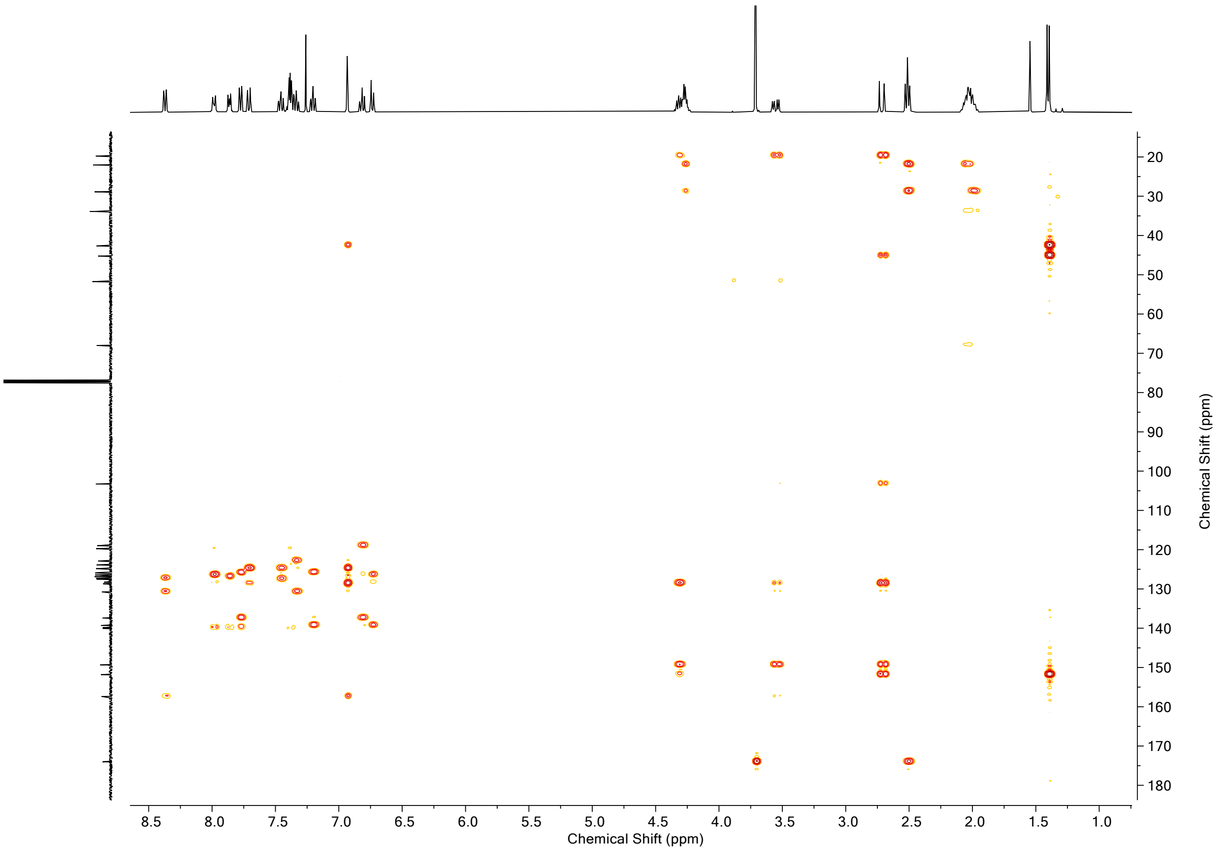

Figure S48: HMBC spectrum of **7b** ($CDCl_3$).

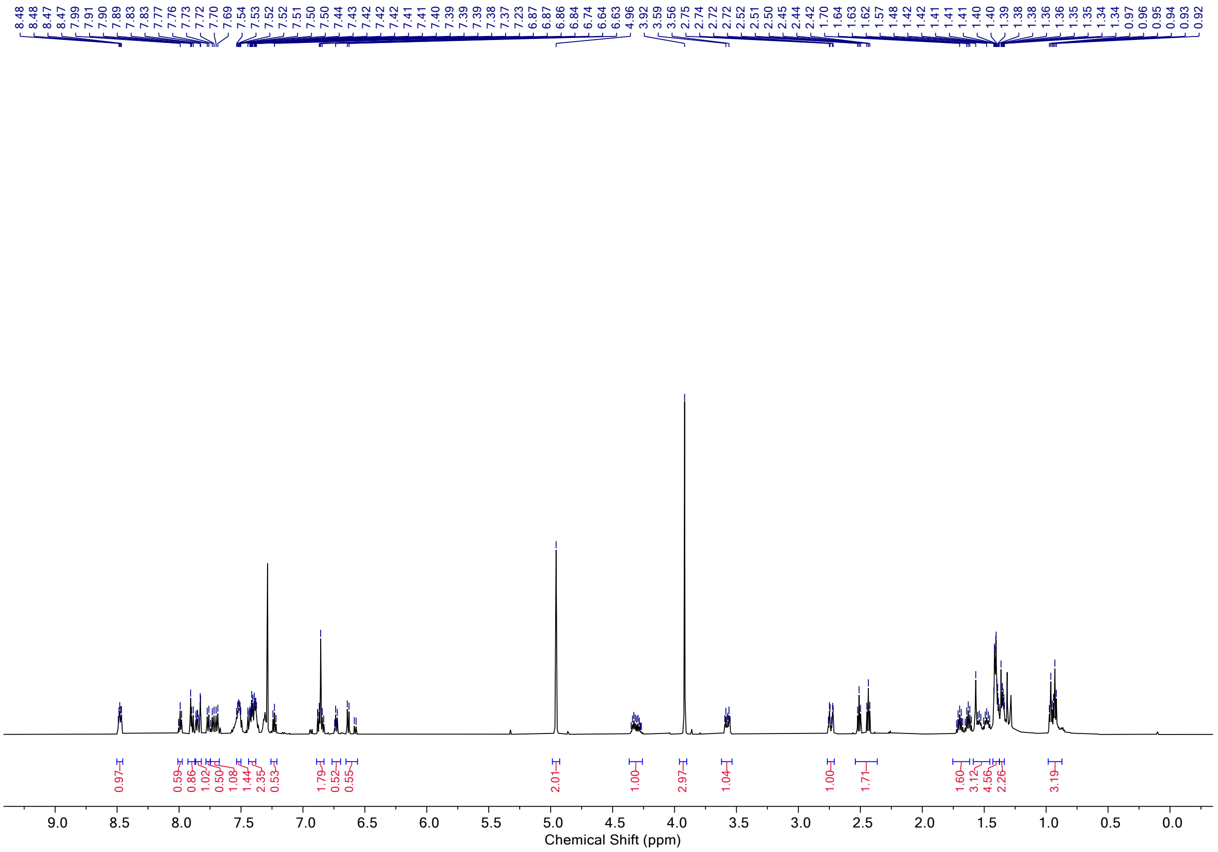

Figure S49: ¹H NMR spectrum of **8** ($CDCl_3$).

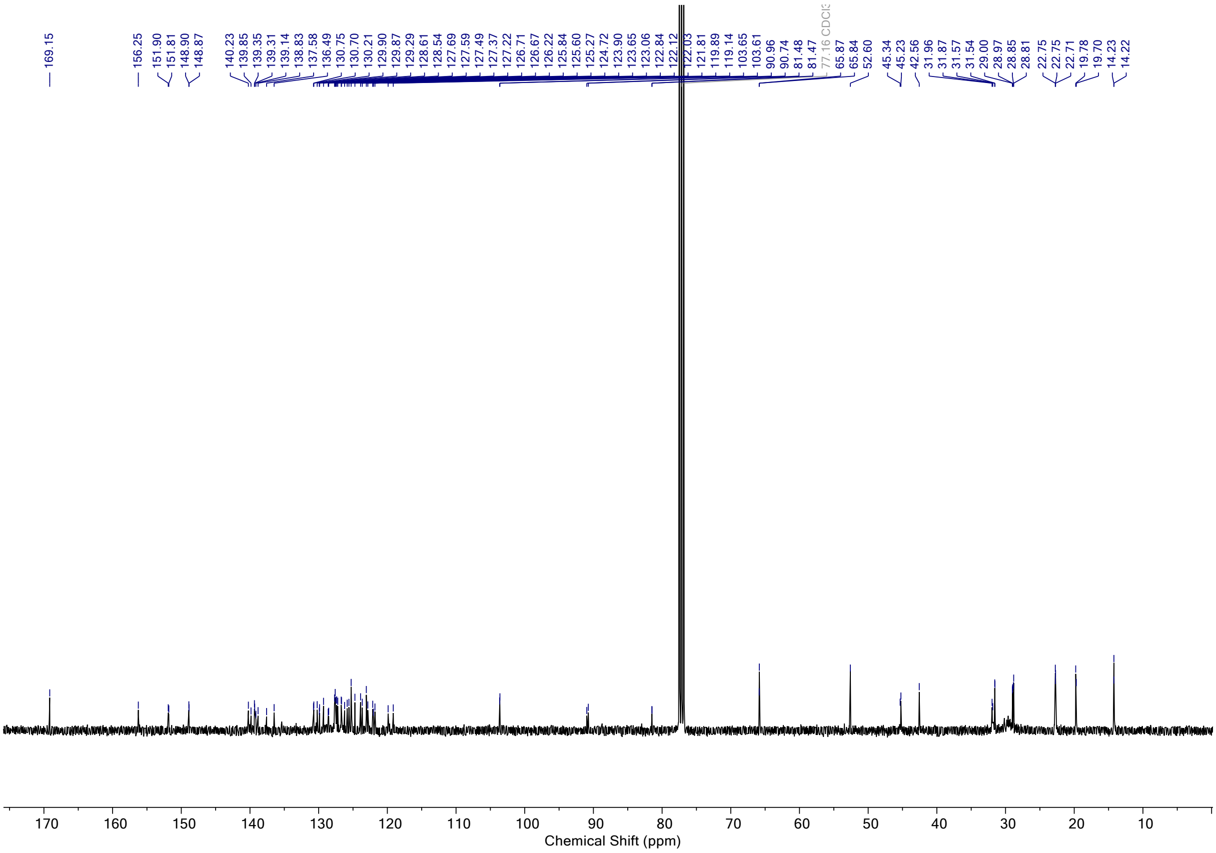

Figure S50: $^{13}C\{^{1}H\}$ **NMR** spectrum of **8** ($CDCl_3$).

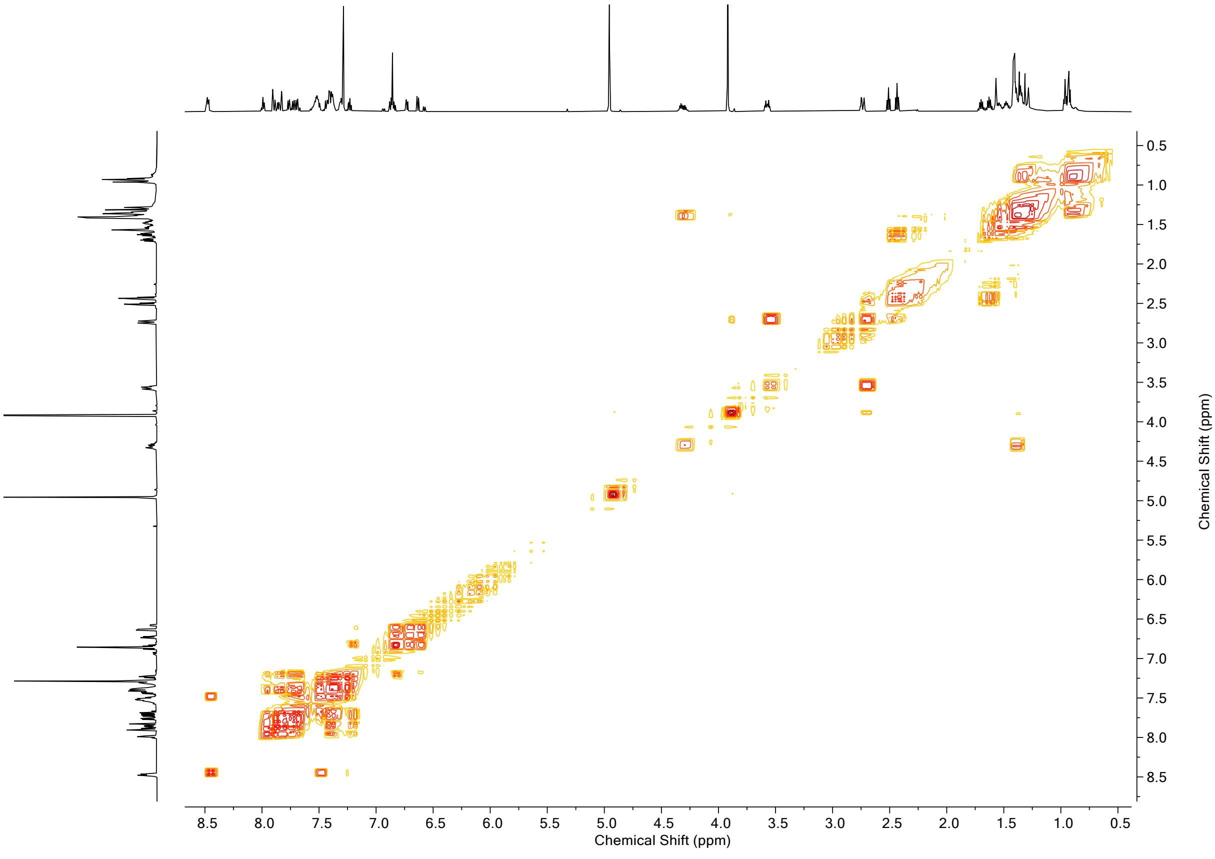

Figure S51: COSY spectrum of **8** ($CDCl_3$).

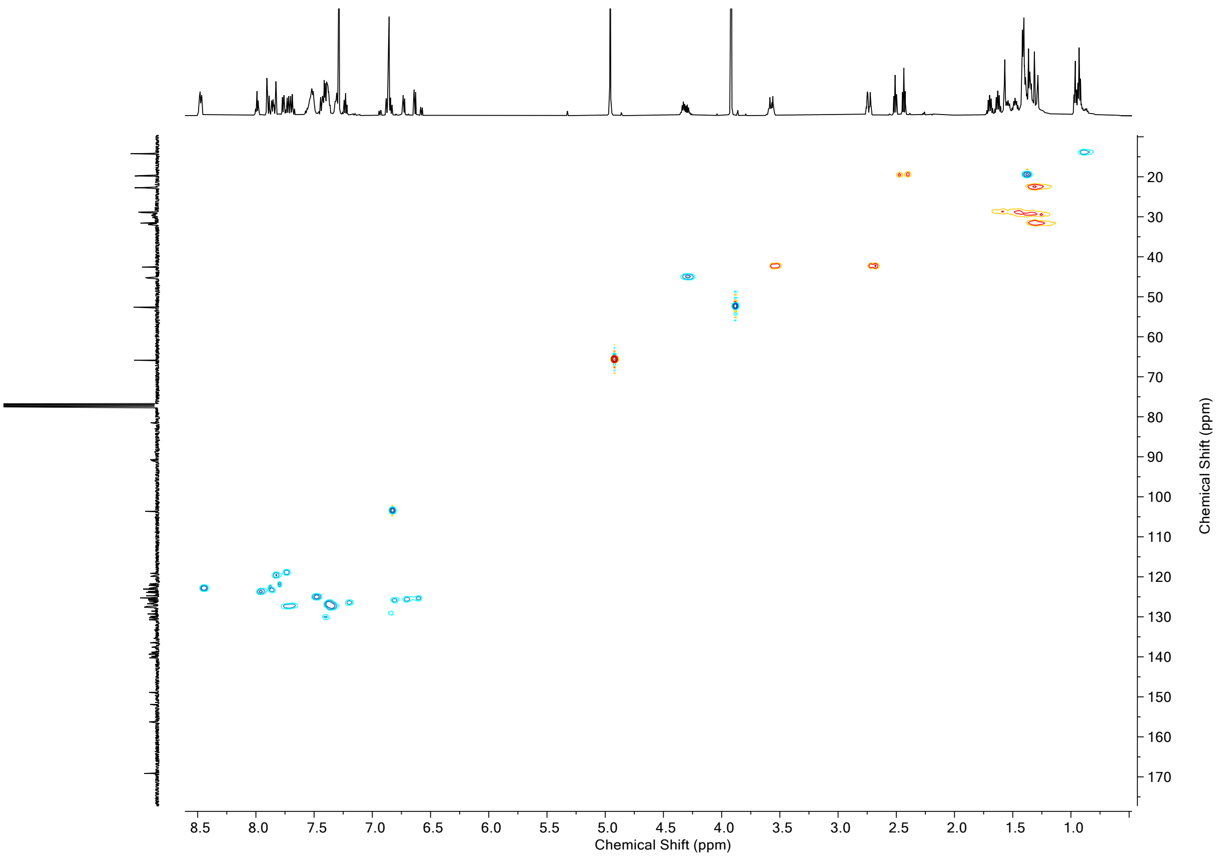

Figure S52: **HSQC** spectrum of **8** ($CDCl_3$).

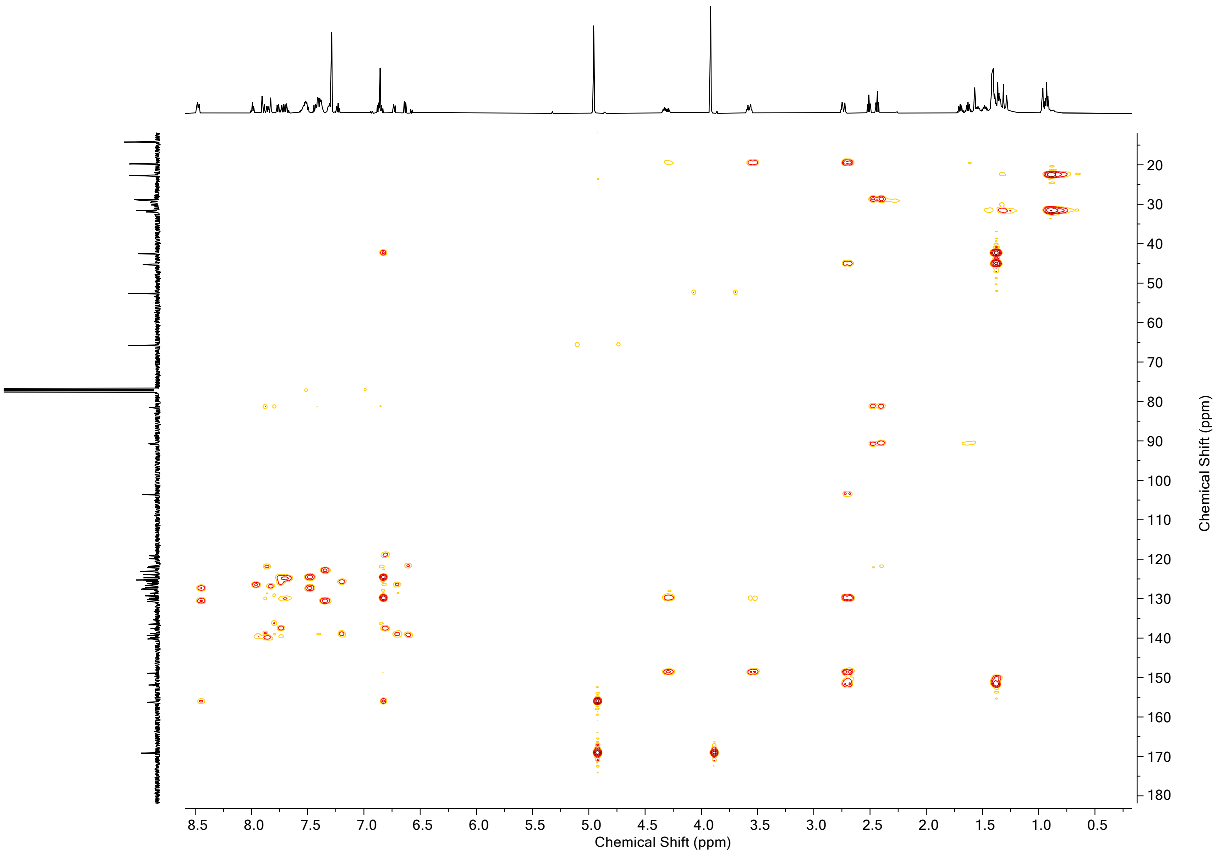

Figure S53: **HMBC** spectrum of **8** ($CDCl_3$).

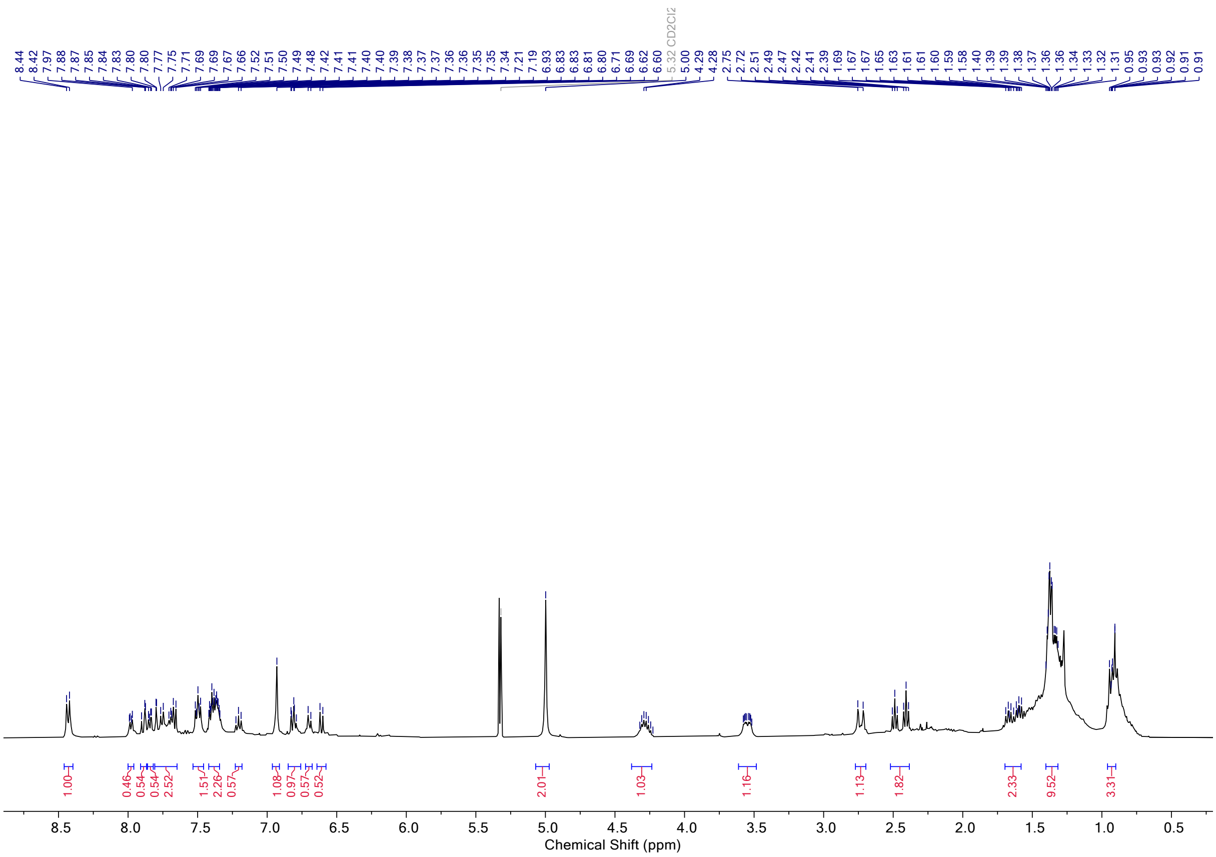

Figure S54: ^{1}H **NMR** spectrum of **MM1** (CD_2Cl_2).

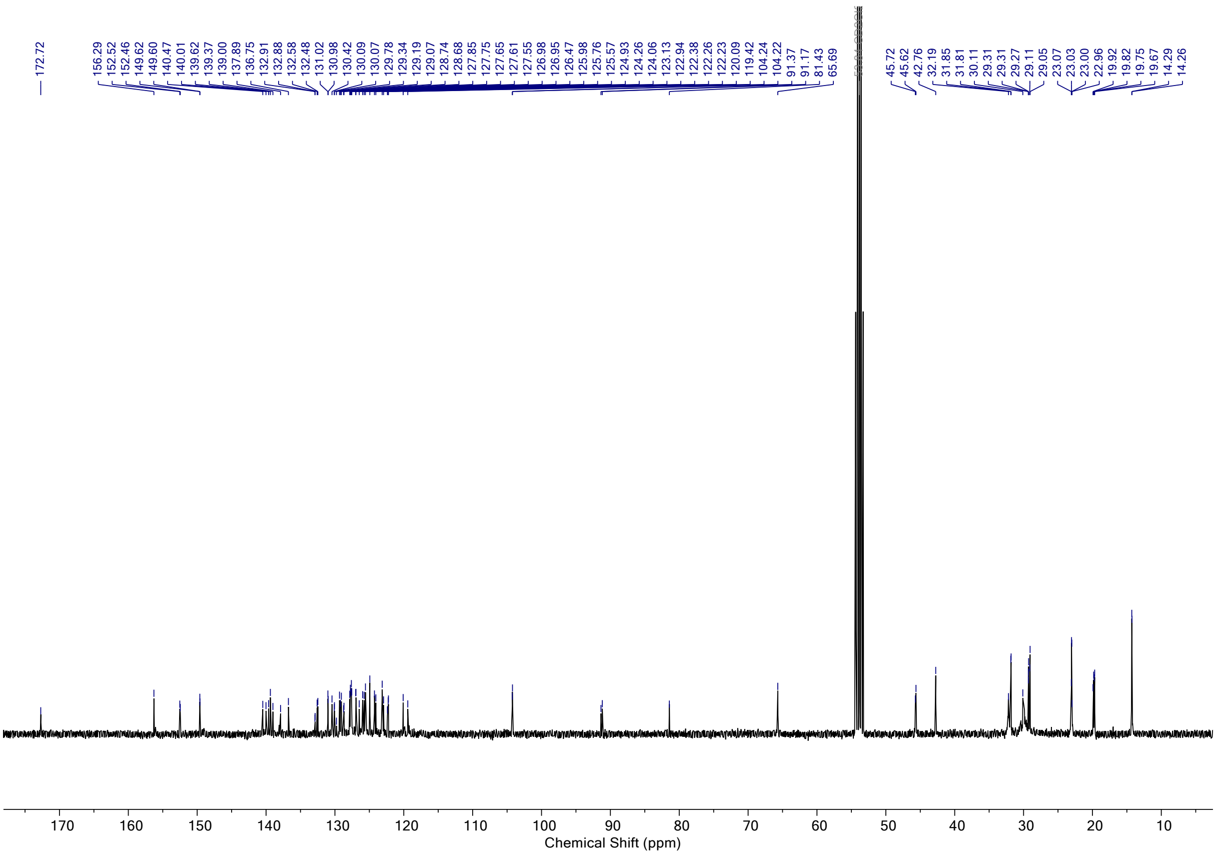

Figure S55: ^{13}C{^{1}H} **NMR** spectrum of **MM1** (CD_2Cl_2).

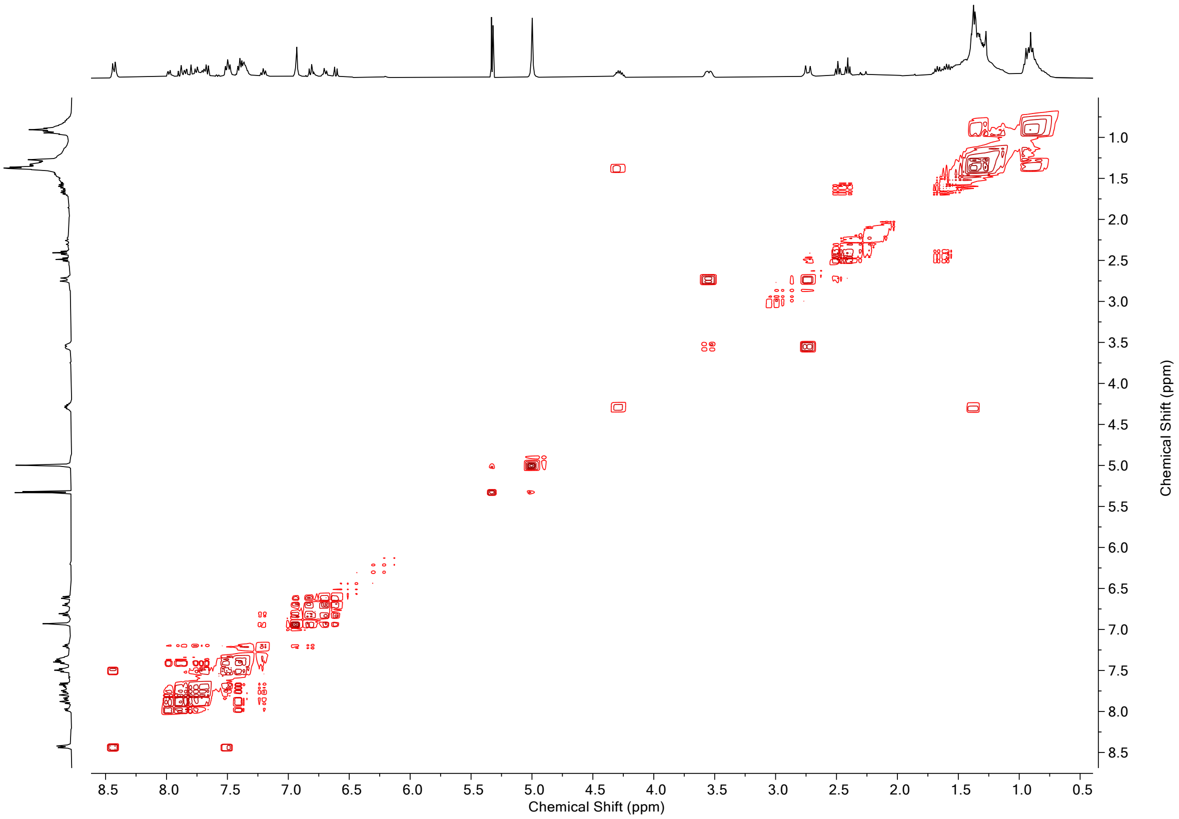

Figure S56: COSY spectrum of **MM1** (CD_2Cl_2).

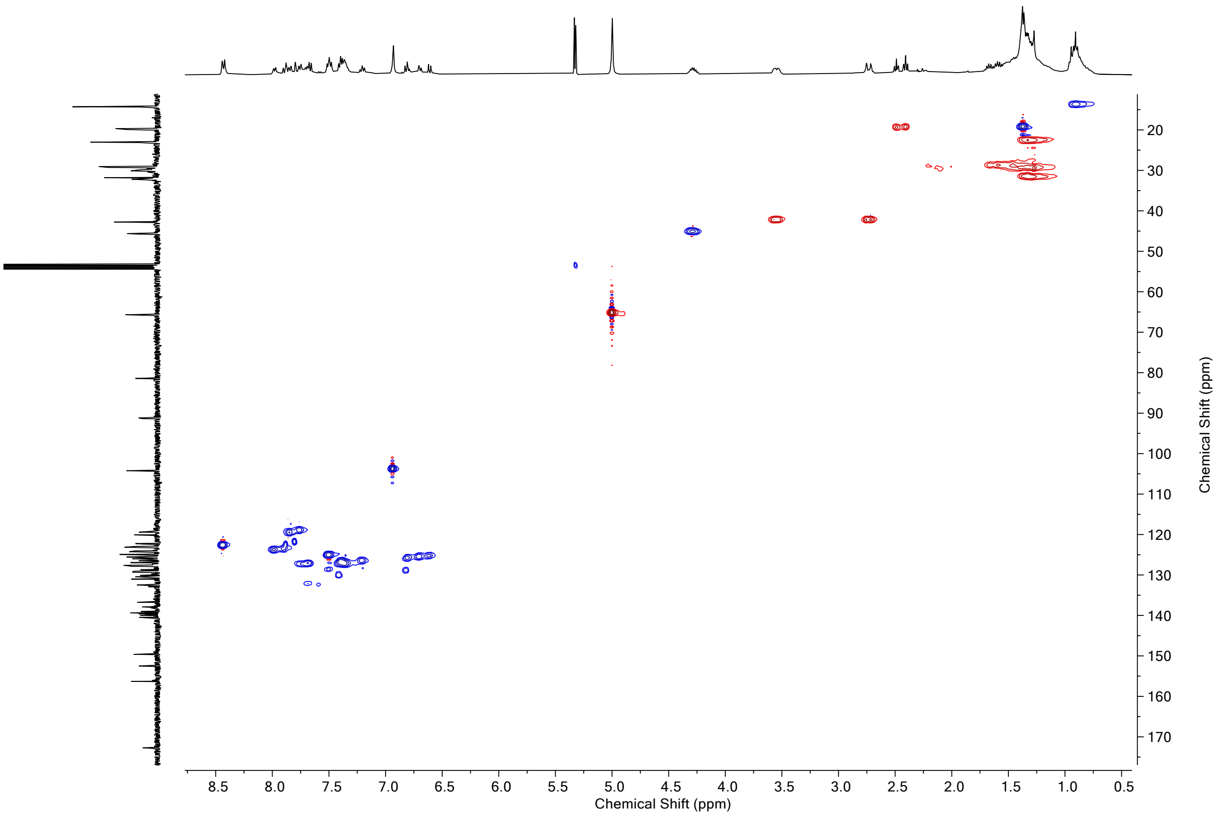

Figure S57: HSQC spectrum of **MM1** (CD_2Cl_2).

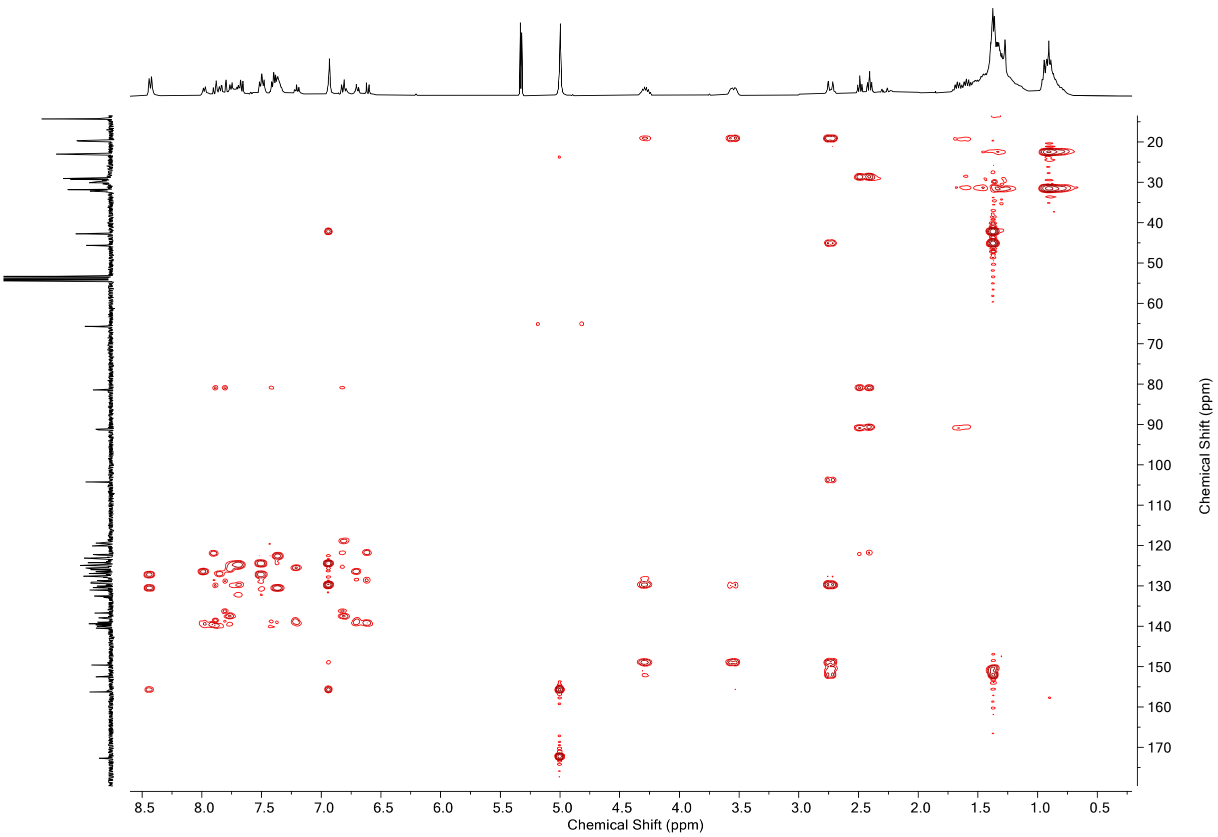

Figure S58: HMBC spectrum of **MM1** (CD_2Cl_2).

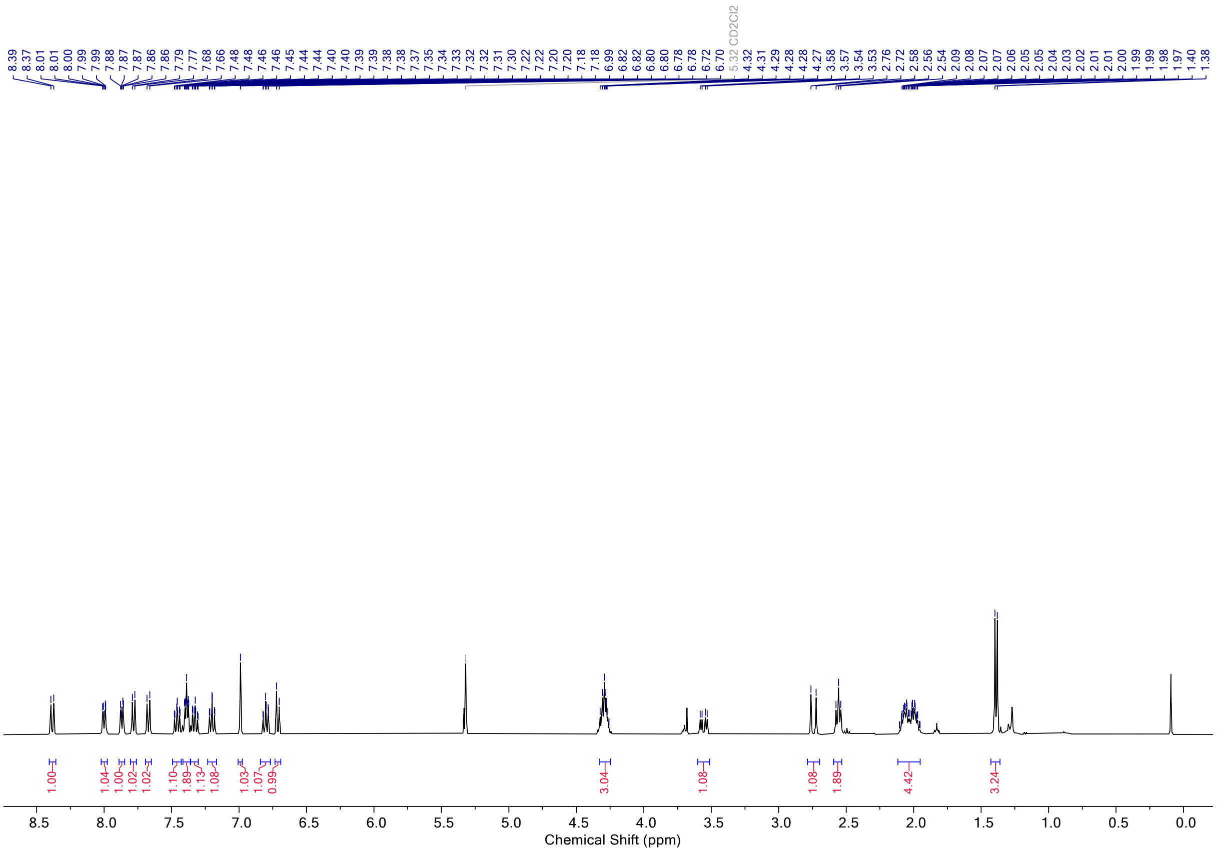

Figure S59: ^{1}**H NMR** spectrum of **MM2** (CD_2Cl_2).

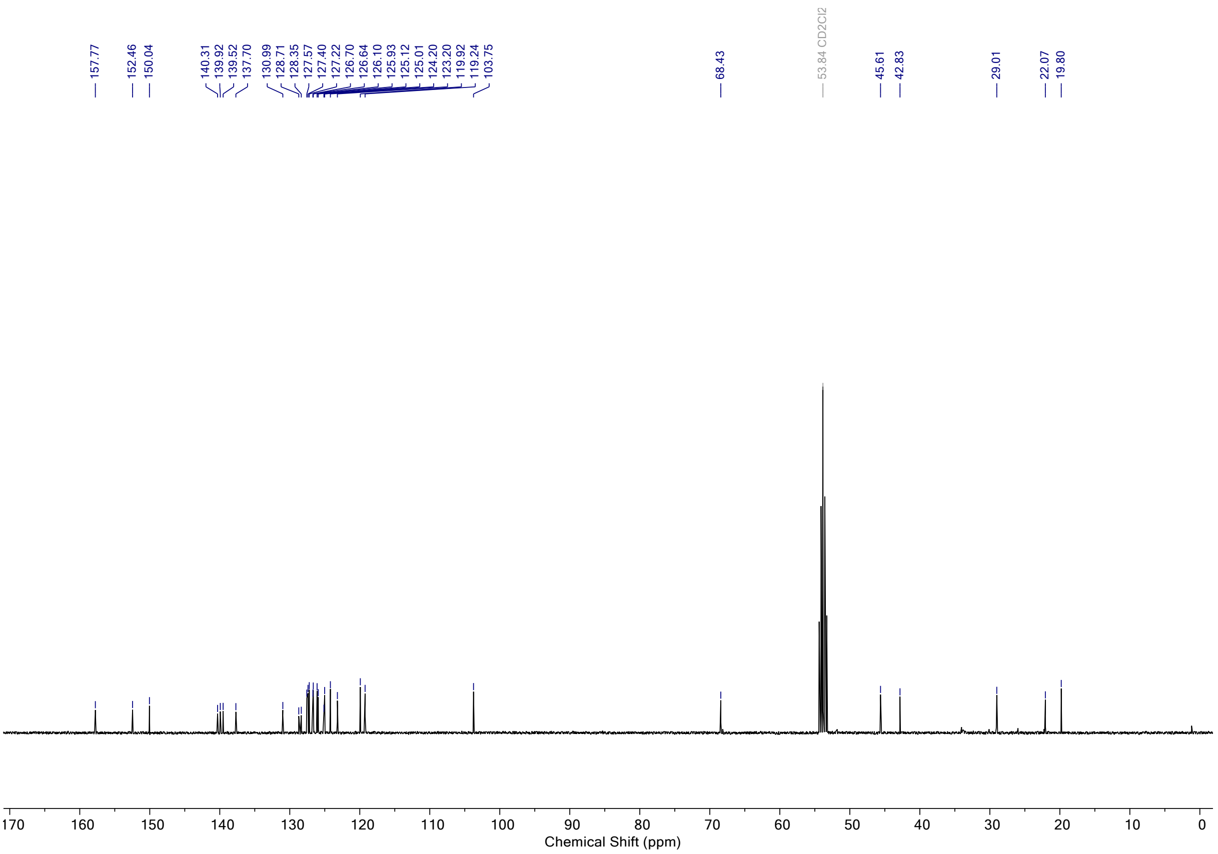

Figure S60: $^{13}C\{^{1}H\}$ **NMR** spectrum of **MM2** (CD_2Cl_2).

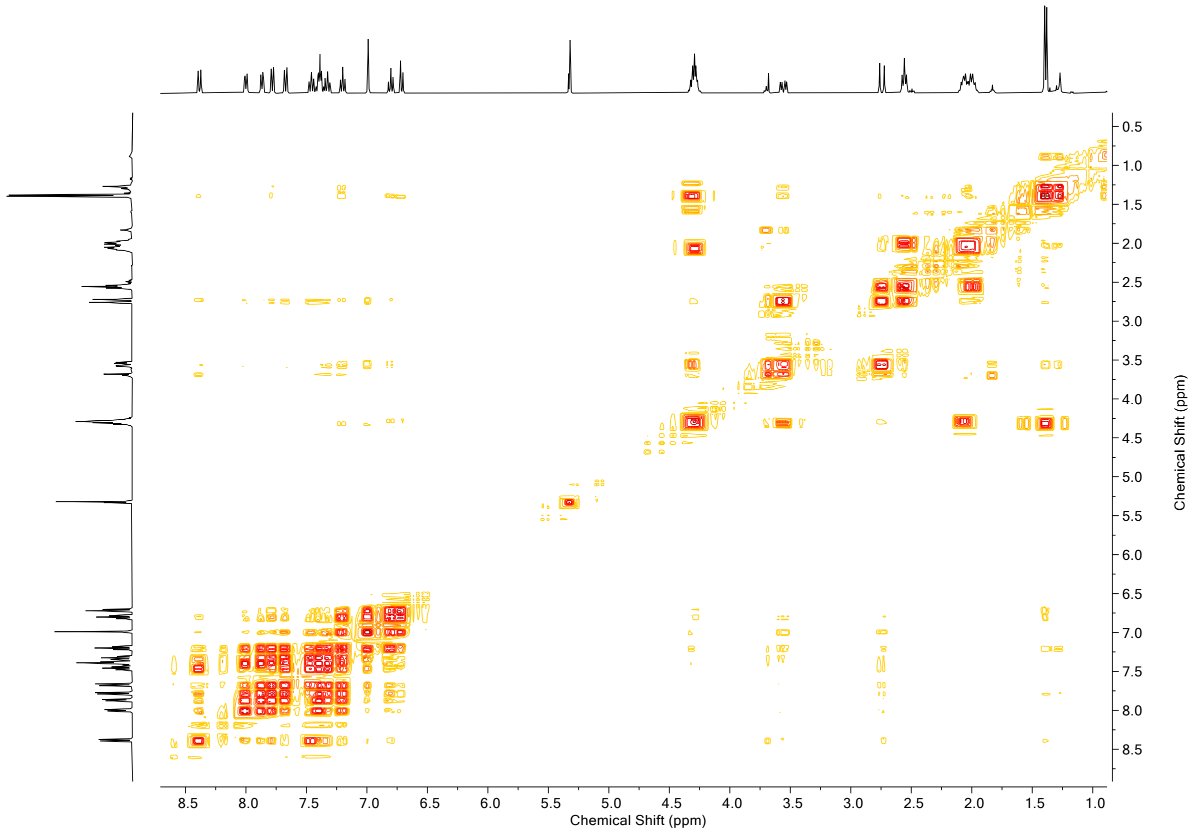

Figure S61: COSY spectrum of **MM2** (CD_2Cl_2).

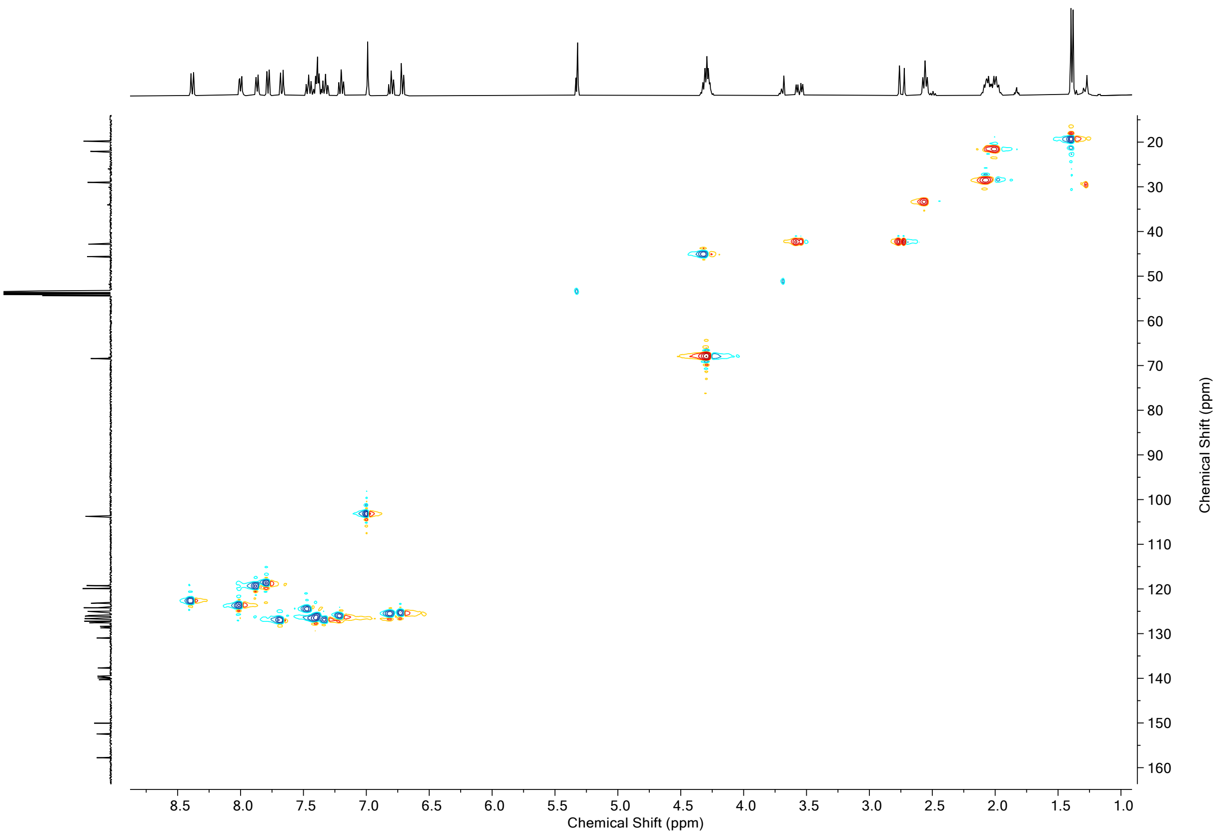

Figure S62: HSQC spectrum of **MM2** (CD_2Cl_2).

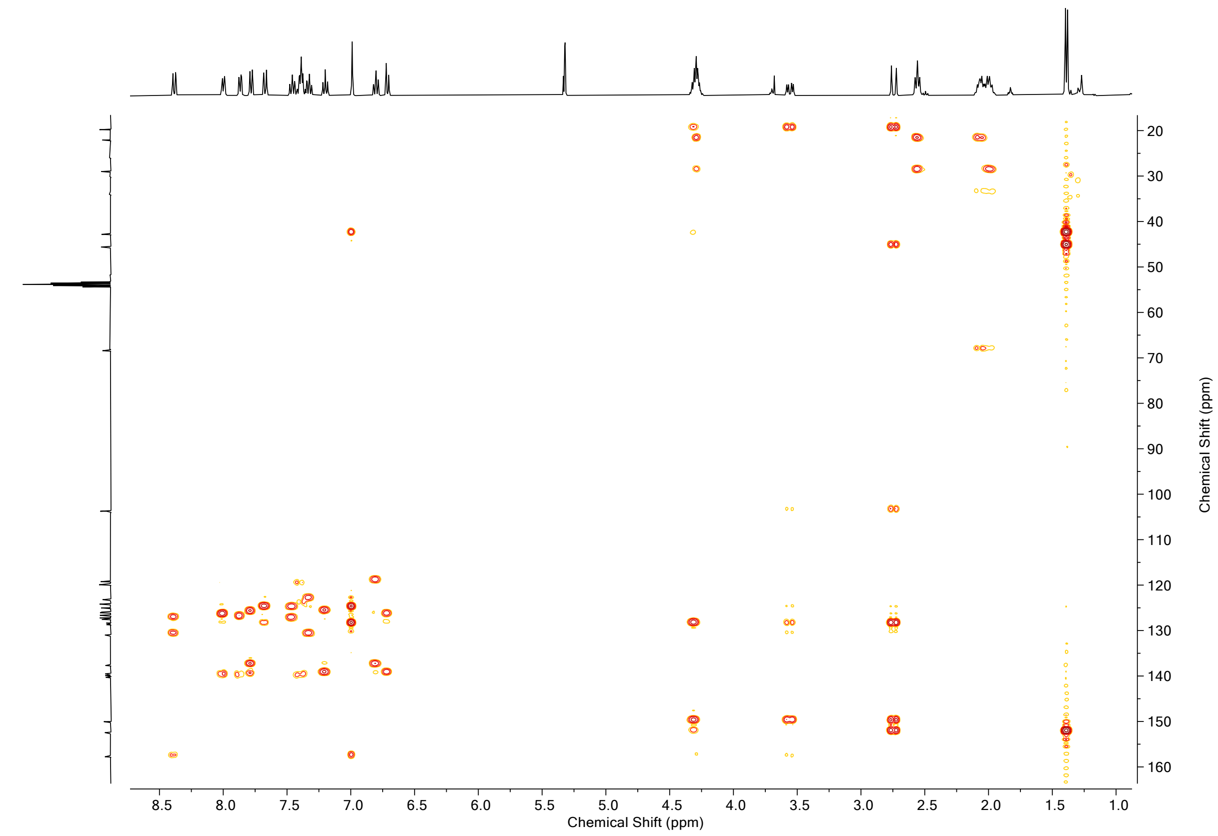

Figure S63: HMBC spectrum of **MM2** (CD_2Cl_2).

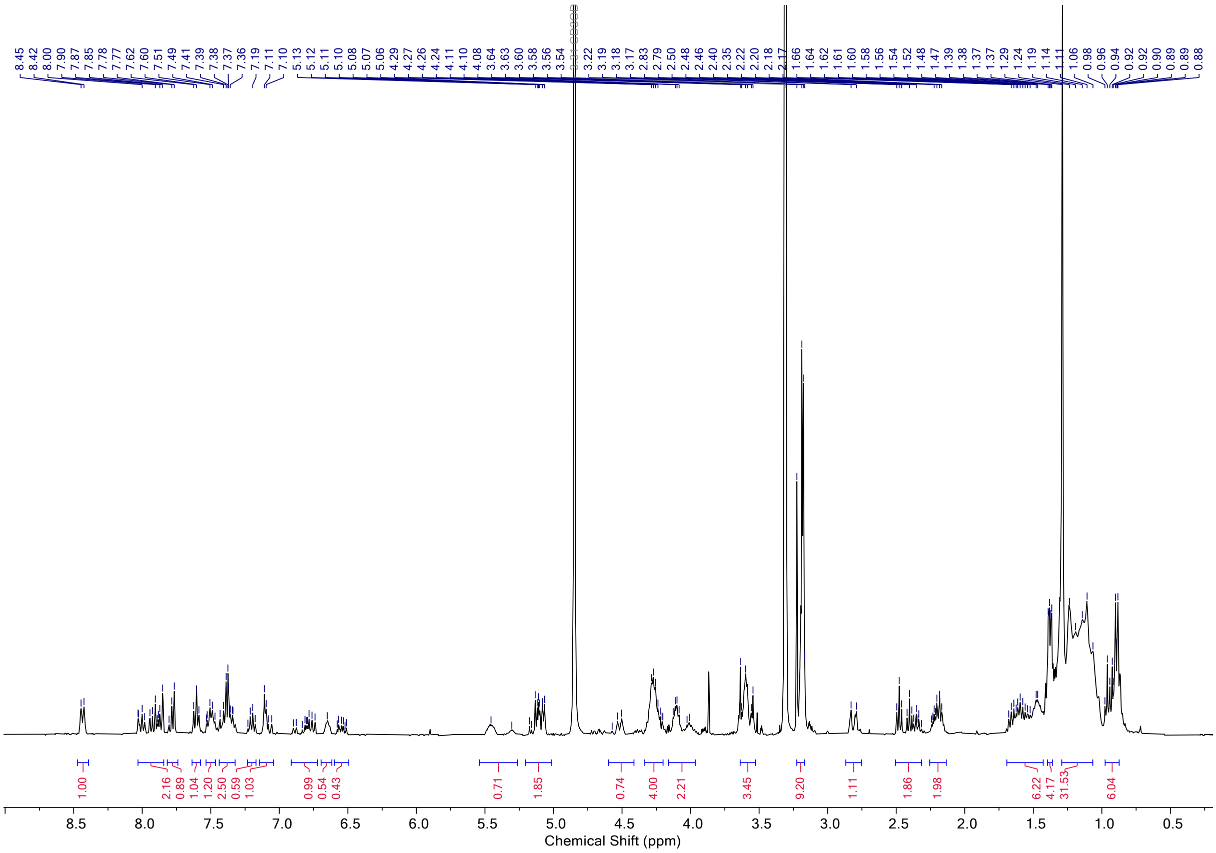

Figure S64: ^{1}**H NMR** spectrum of **MM1-PC** (CD_3OD).

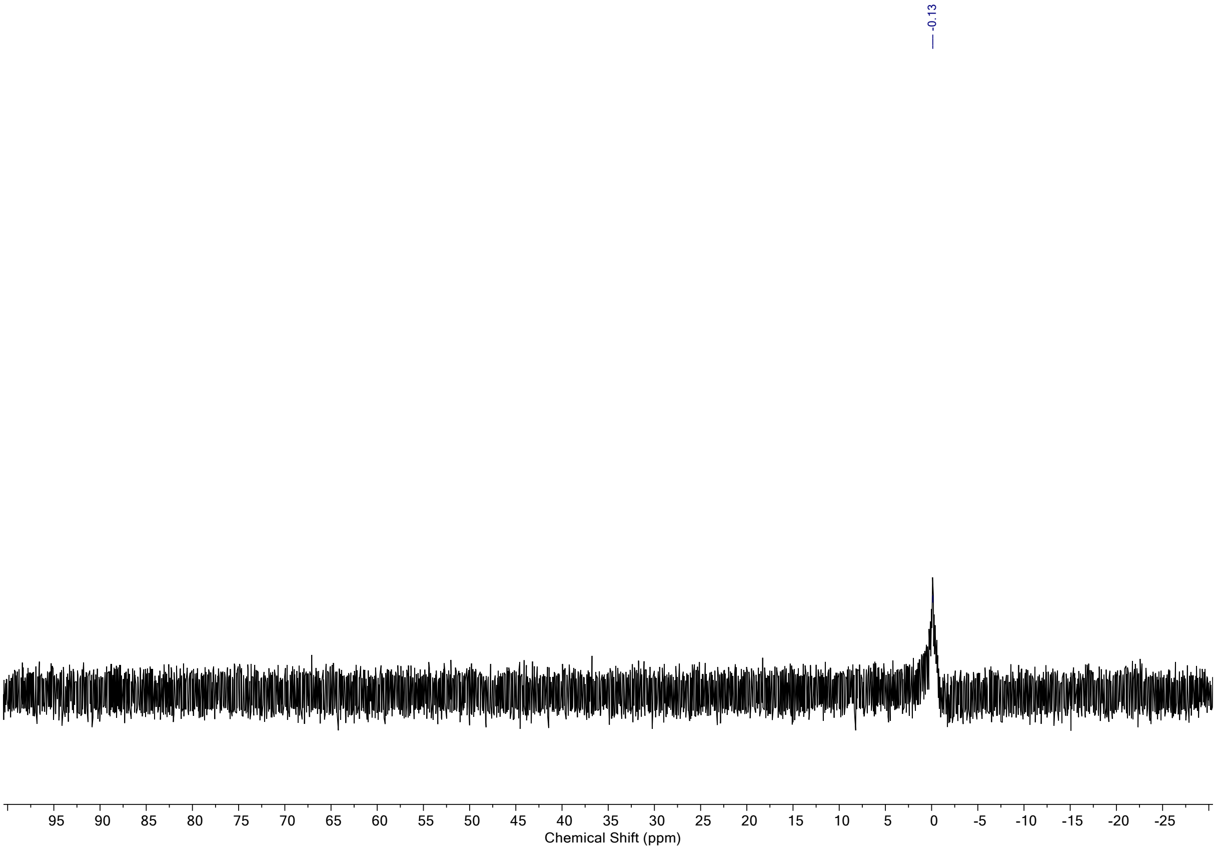

Figure S65: ^{31}**P NMR** spectrum of **MM1-PC** (CD_3OD).

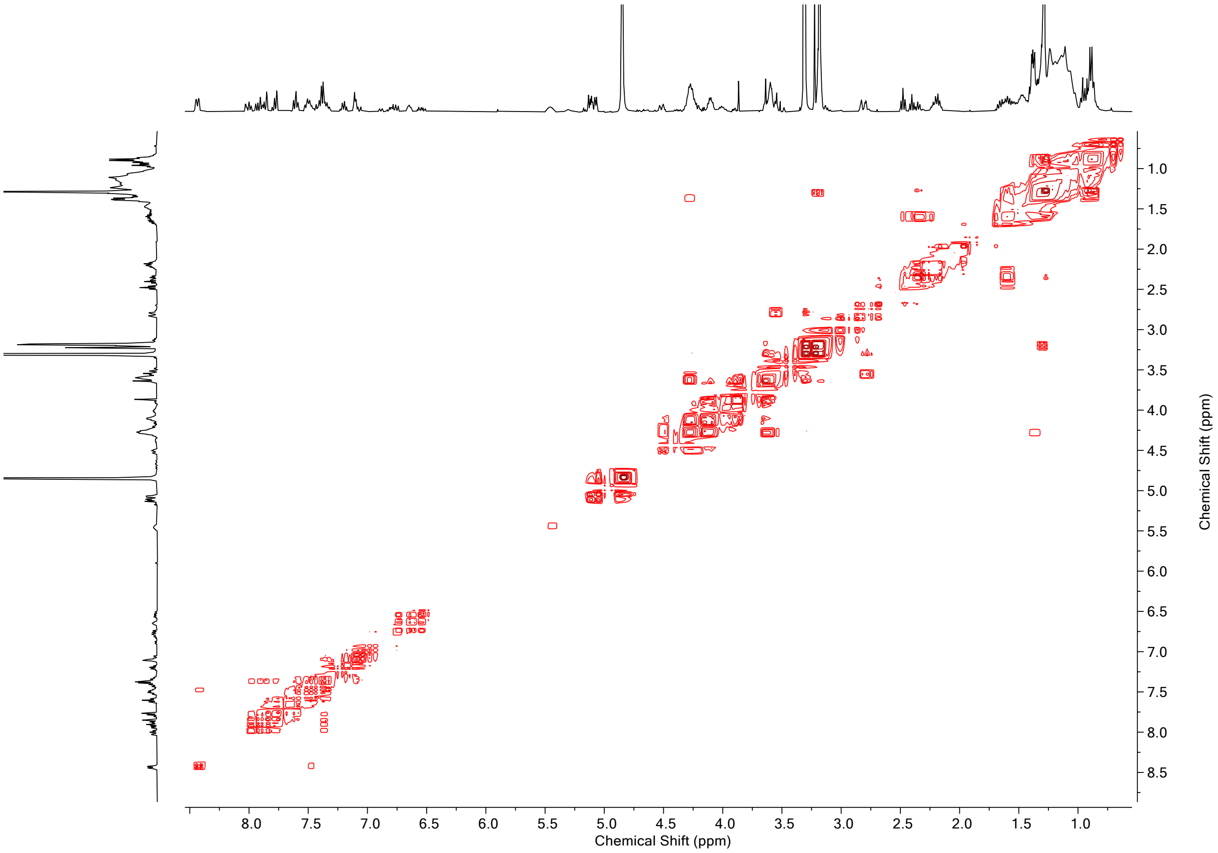

Figure S66: COSY spectrum of **MM1-PC** (CD_3OD).

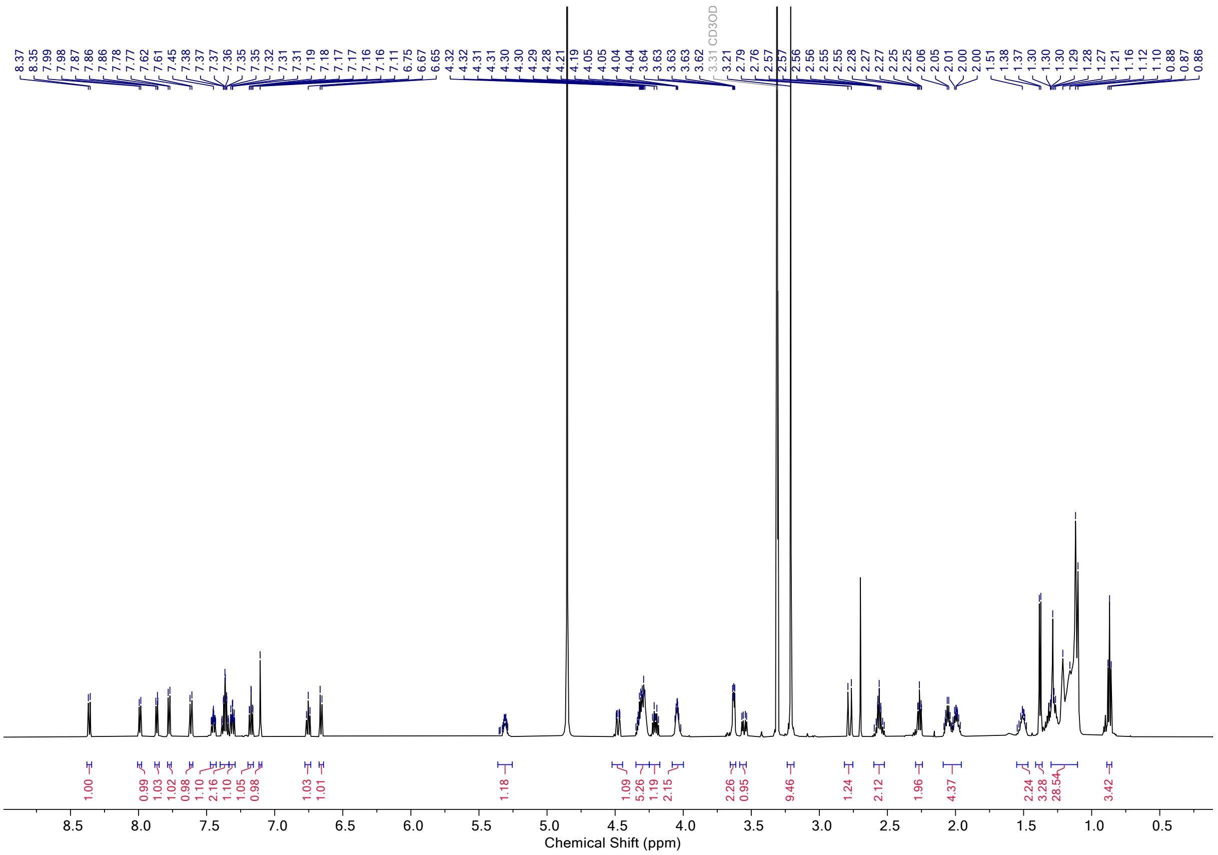

Figure S67: ^{1}**H NMR** spectrum of **MM2-PC** (CD_3OD).

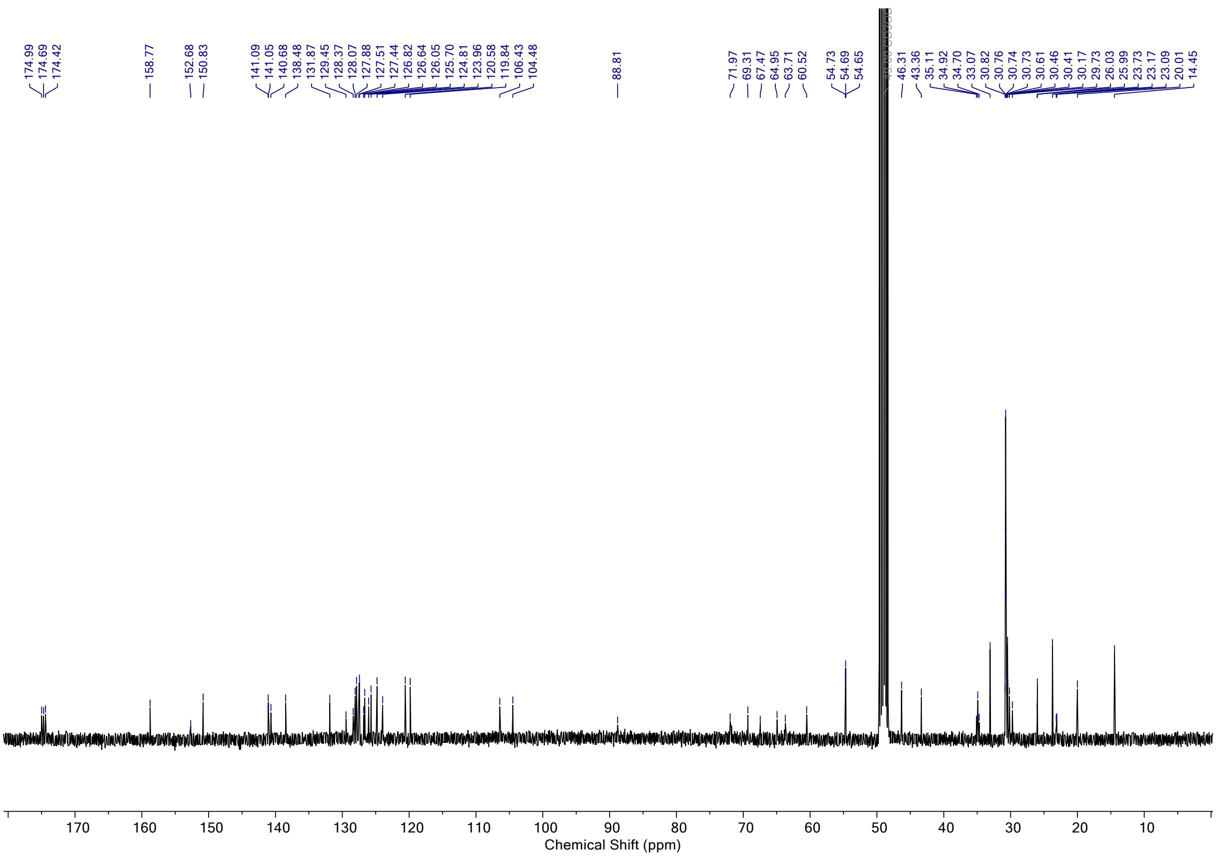

Figure S68: ^{13}C{^{1}H} **NMR** spectrum of **MM2-PC** (CD_3OD).

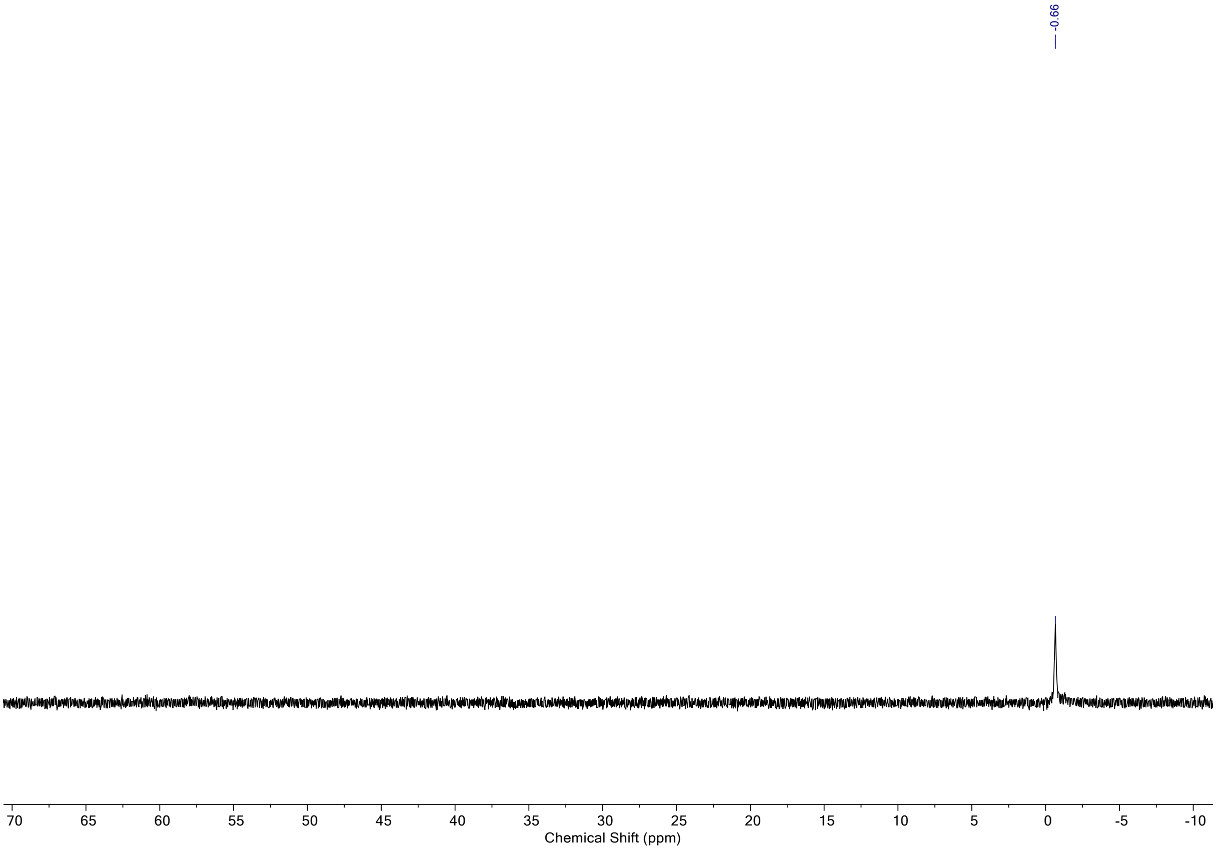

Figure S69: ^{31}P **NMR** spectrum of **MM2-PC** (CD_3OD).

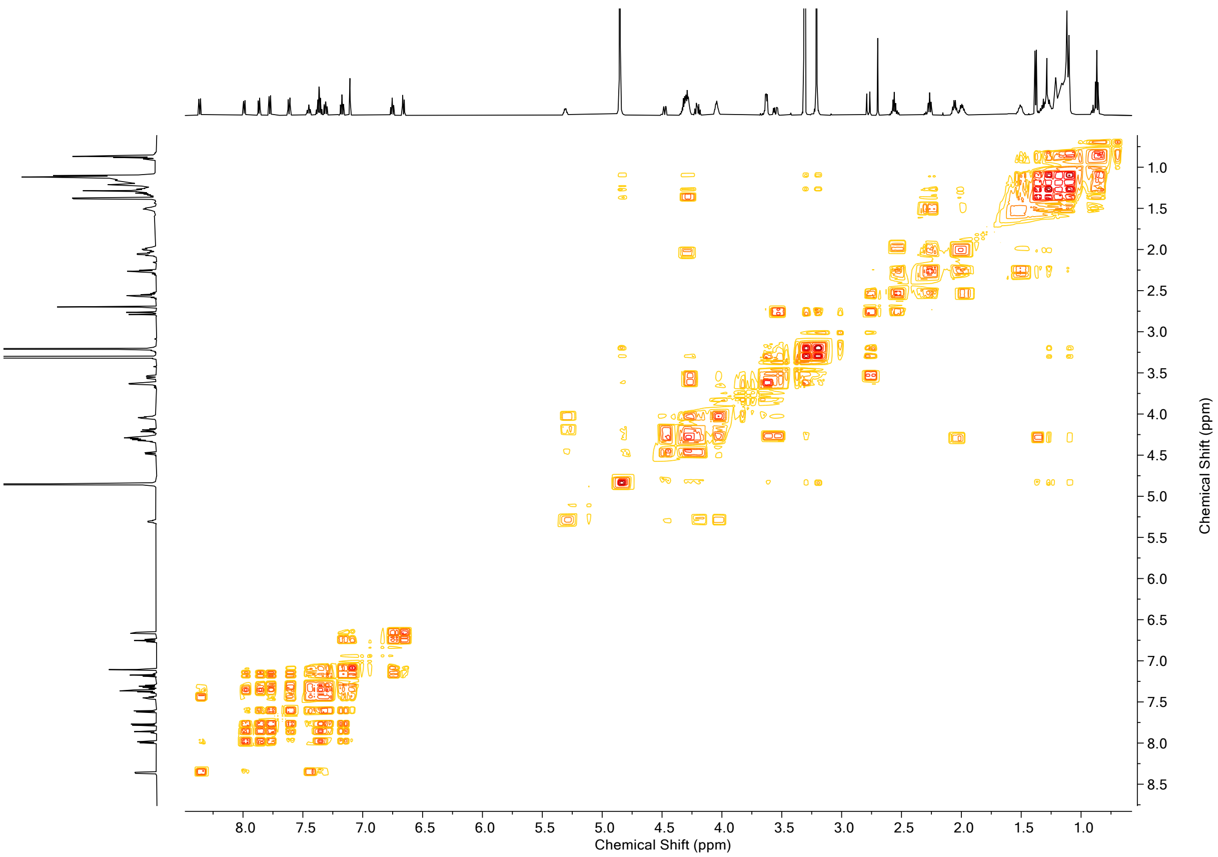

Figure S70: COSY spectrum of **MM2-PC** (CD_3OD).

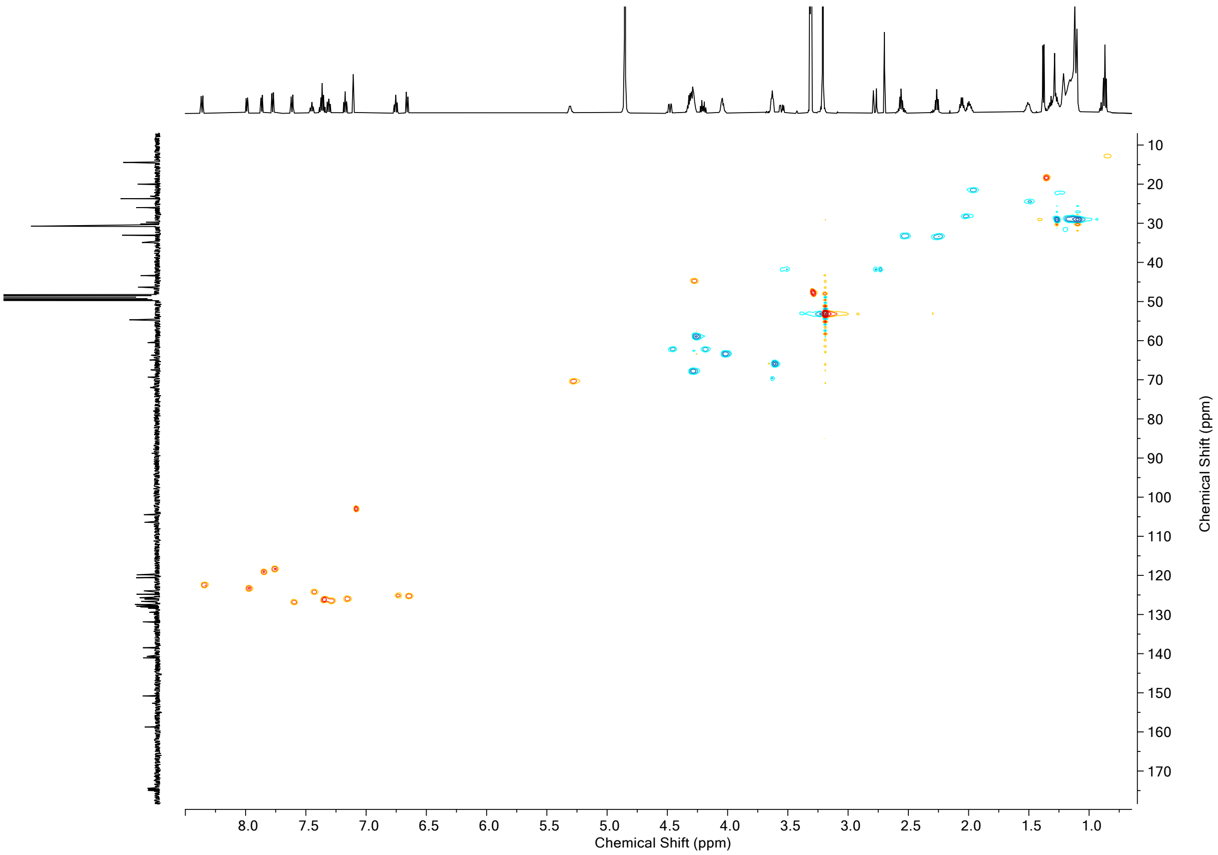

Figure S71: HSQC spectrum of **MM2-PC** (CD_3OD).

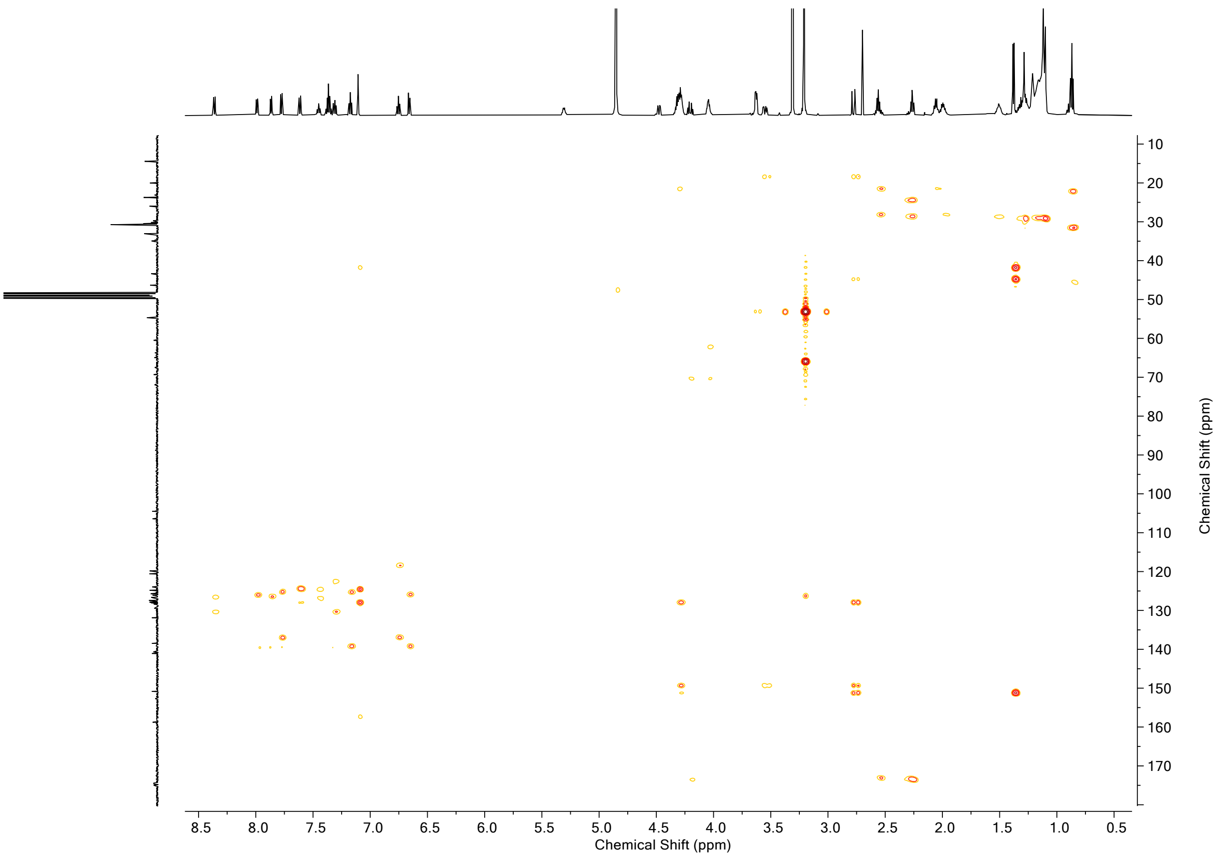

Figure S72: HMBC spectrum of **MM2-PC** (CD_3OD).
